# Supplementary material for: Synthesis and In Silico Profile Modeling of 6-O-Fluoroalkyl-6-O-desmethyl-diprenorphine Analogs
Source: Int J Mol Sci. 2025 Sep 26;26(19):9427. doi: 10.3390/ijms26199427 (PMC12524955; doi:10.3390/ijms26199427)

Supplementary Materials

Synthesis and in silico profile modeling of 6-*O*-fluoroalkyl-6-*O*-desmethyl-diprenorphine analogues

János Marton [1,*,†], Dávid Gombos [2,3,†], Paul Cumming [4,5], Tamás Fehér [2,6], Alexander Milentyev [1], Beate Bauer [1], Frode Willoch [7], Bent Wilhelm Schoultz [8,9], Sándor Benyhe [2] and Ferenc Ötvös [2,*]

[1] ABX Advanced Biochemical Compounds Biomedizinische Forschungsreagenzien GmbH, Heinrich-Glaeser-Strasse 10-14, D-01454 Radeberg, Germany; milentyev@abx.de (A.M.); beatebauer@t-online.de (B.B.)
[2] Institute of Biochemistry, HUN-REN Biological Research Center, Temesvári krt. 62, H-6726 Szeged, Hungary; gombosda@brc.hu (D.G.); feher.tamas@brc.hu (T.F.), benyhe.sandor@brc.hu (S.B.)
[3] Doctoral School of Theoretical Medicine, Faculty of Medicine, University of Szeged, Dugonics tér 13, H-6720 Szeged, Hungary
[4] School of Psychology and Counselling, Queensland University of Technology, Brisbane, QLD 4059, Australia; paul.k.cumming@gmail.com
[5] Department of Nuclear Medicine, Bern University Hospital, Freiburgstraße 18, CH-3010 Bern, Switzerland
[6] Synthetic and Systems Biology Unit, HUN-REN Biological Research Center, 62 Temesvari krt., H-6726 Szeged, Hungary
[7] Institute of Basic Medical Sciences, University of Oslo, P. O. Box 1110, Blindern, N-0317 Oslo, Norway
[8] Department of Physics, University of Oslo, P.O. Box 1048, Blindern, N-0316 Oslo, Norway; b.w.schoultz@fys.uio.no
[9] Norwegian Medical Cyclotron Centre Ltd., Sognsvannsveien 20, N-0372 Oslo, Norway; bent.wilhelm.schoultz@syklotronsenteret.no
* Correspondence: marton@abx.de (J.M.); otvos.ferenc@brc.hu (F.Ö.)
† These authors contributed equally to this work.

CONTENTS

		Page
**1**	**Abbreviations**	4–5
**Table S1**	^{1}H and ^{13}C NMR chemical shifts and coupling constants of FP-TDDPN (**27b**), FB-TDDPN (**27c**) and FPe-TDDPN (**27d**)a in $CDCl_3$	6–7
**Table S2**	^{1}H and ^{13}C NMR chemical shifts and coupling constants of HP-DPN (**29b**), HB-DPN (**29c**) and HPe-DPN (**29d**) in $CDCl_3$	7
**Table S3**	Reaction conditions, yields, and physical constants for compounds prepared	8
**Chart S1**	Chemical structures of selected orvinol an 6-*O*-desmethyl-orvinol derivatives	8

Spectral data for the compounds prepared (Figure S1-S57, ^{1}H-NMR, ^{13}C-NMR and ^{19}F-NMR spectra)

Figure	Spectrum	Page
**Figure S1**	^{1}H-NMR spectrum of 6-*O*-(3-*tert*-butyldiphenylsilyloxypropyl)-6-*O*-desmethyl-3-*O*-trityl-diprenorphine (**24b**, TBDPS-OP-TDDPN) in $CDCl_3$	9
**Figure S2**	^{13}C-NMR spectrum of 6-*O*-(3-*tert*-butyldiphenylsilyloxypropyl)-6-*O*-desmethyl-3-*O*-trityl-diprenorphine (**24b**, TBDPS-OP-TDDPN) in $CDCl_3$	10
**Figure S3**	^{1}H-NMR spectrum of 6-*O*-(4-*tert*-butyldiphenylsilyloxybutyl)-6-*O*-desmethyl-3-*O*-trityl-diprenorphine (**24c**, TBDPS-OB-TDDPN) in $CDCl_3$	11
**Figure S4**	^{13}C-NMR spectrum of 6-*O*-(4-*tert*-butyldiphenylsilyloxybutyl)-6-*O*-desmethyl-3-*O*-trityl-diprenorphine (**24c**, TBDPS-OB-TDDPN) in $CDCl_3$	12
**Figure S5**	^{1}H-NMR spectrum of 6-*O*-(5-*tert*-butyldiphenylsilyloxypentyl)-6-*O*-desmethyl-3-*O*-trityl-diprenorphine (**24d**, TBDPS-OPe-TDDPN) in $CDCl_3$	13
**Figure S6**	^{13}C-NMR spectrum of 6-*O*-(5-*tert*-butyldiphenylsilyloxypentyl)-6-*O*-desmethyl-3-*O*-trityl-diprenorphine (**24d**, TBDPS-OPe-TDDPN) in $CDCl_3$	14
**Figure S7**	^{1}H-NMR spectrum of 6-*O*-(3-hydroxypropyl)-6-*O*-desmethyl-3-*O*-trityl-diprenorphine (**25b**, HP-TDDPN) in $CDCl_3$	15
**Figure S8**	^{13}C-NMR spectrum of 6-*O*-(3-hydroxypropyl)-6-*O*-desmethyl-3-*O*-trityl-diprenorphine (**25b**, HP-TDDPN) in $CDCl_3$	16
**Figure S9**	^{1}H-NMR spectrum of 6-*O*-(4-hydroxybutyl)-6-*O*-desmethyl-3-*O*-trityl-diprenorphine (**25c**, HB-TDDPN) in $CDCl_3$	17
**Figure S10**	^{13}C-NMR spectrum of 6-*O*-(4-hydroxybutyl)-6-*O*-desmethyl-3-*O*-trityl-diprenorphine (**25c**, HB-TDDPN) in $CDCl_3$	18
**Figure S11**	^{1}H-NMR spectrum of 6-*O*-(5-hydroxypentyl)-6-*O*-desmethyl-3-*O*-trityl-diprenorphine (**25d**, HPe-TDDPN) in $CDCl_3$	19
**Figure S12**	^{13}C-NMR spectrum of 6-*O*-(5-hydroxypentyl)-6-*O*-desmethyl-3-*O*-trityl-diprenorphine (**25d**, HPe-TDDPN) in $CDCl_3$	20
**Figure S13**	^{1}H-NMR spectrum of 6-*O*-(3-tosyloxypropyl)-6-*O*-desmethyl-3-*O*-trityl-diprenorphine (**26b**, TP-TDDPN) in $CDCl_3$	21
**Figure S14**	^{13}C-NMR spectrum of 6-*O*-(3-tosyloxypropyl)-6-*O*-desmethyl-3-*O*-trityl-diprenorphine (**26b**, TP-TDDPN) in $CDCl_3$	22
**Figure S15**	^{1}H-NMR spectrum of 6-*O*-(4-tosyloxybutyl)-6-*O*-desmethyl-3-*O*-trityl-diprenorphine (**26c**, TB-TDDPN) in $CDCl_3$	23
**Figure S16**	^{13}C-NMR spectrum of 6-*O*-(4-tosyloxybutyl)-6-*O*-desmethyl-3-*O*-trityl-diprenorphine (**26c**, TB-TDDPN) in $CDCl_3$	24
**Figure S17**	^{1}H-NMR spectrum of 6-*O*-(5-tosyloxypentyl)-6-*O*-desmethyl-3-*O*-trityl-diprenorphine (**26d**, TPe-TDDPN) in $CDCl_3$	25
**Figure S18**	^{13}C-NMR spectrum of 6-*O*-(5-tosyloxypentyl)-6-*O*-desmethyl-3-*O*-trityl-diprenorphine (**26d**, TPe-TDDPN) in $CDCl_3$	26
**Figure S19**	^{1}H-NMR spectrum of 6-*O*-(3-fluoropropyl)-6-*O*-desmethyl-3-*O*-trityl-diprenorphine (**27b**, FP-TDDPN) in $CDCl_3$	27
**Figure S20**	^{13}C-NMR spectrum of 6-*O*-(3-fluoropropyl)-6-*O*-desmethyl-3-*O*-trityl-diprenorphine (**27b**, FP-TDDPN) in $CDCl_3$	28
**Figure S21**	^{19}F-NMR spectrum of 6-*O*-(3-fluoropropyl)-6-*O*-desmethyl-3-*O*-trityl-diprenorphine (**27b**, FP-TDDPN) in $CDCl_3$	29

**Figure S22**	^{1}H-NMR spectrum of 6-*O*-(4-fluorobutyl)-6-*O*-desmethyl-3-*O*-trityl-diprenorphine (**27c**, FB-TDDPN) in $CDCl_3$	30
**Figure S23**	^{13}C-NMR spectrum of 6-*O*-(4-fluorobutyl)-6-*O*-desmethyl-3-*O*-trityl-diprenorphine (**27c**, FB-TDDPN) in $CDCl_3$	31
**Figure S24**	^{19}F-NMR spectrum of 6-*O*-(4-fluorobutyl)-6-*O*-desmethyl-3-*O*-trityl-diprenorphine (**27c**, FB-TDDPN) in $CDCl_3$	32
**Figure S25**	^{1}H-NMR spectrum of 6-*O*-(5-fluoropentyl)-6-*O*-desmethyl-3-*O*-trityl-diprenorphine (**27d**, FPe-TDDPN) in $CDCl_3$	33
**Figure S26**	^{13}C-NMR spectrum of 6-*O*-(5-fluoropentyl)-6-*O*-desmethyl-3-*O*-trityl-diprenorphine (**27d**, FPe-TDDPN) in $CDCl_3$	34
**Figure S27**	^{19}F-NMR spectrum of 6-*O*-(5-fluoropentyl)-6-*O*-desmethyl-3-*O*-trityl-diprenorphine (**27d**, FPe-TDDPN) in $CDCl_3$	35
**Figure S28**	^{1}H-NMR spectrum of 6-*O*-(3-fluoropropyl)-6-*O*-desmethyl-diprenorphine (**28b**, FP-DPN) in $CDCl_3$	36
**Figure S29**	^{13}C-NMR spectrum of 6-*O*-(3-fluoropropyl)-6-*O*-desmethyl-diprenorphine (**28b**, FP-DPN) in $CDCl_3$	37
**Figure S30**	^{19}F-NMR spectrum of 6-*O*-(3-fluoropropyl)-6-*O*-desmethyl-diprenorphine (**28b**, FP-DPN) in $CDCl_3$	38
**Figure S31**	^{1}H-NMR spectrum of 6-*O*-(4-fluorobutyl)-6-*O*-desmethyl-diprenorphine (**28c**, FB-DPN) in $CDCl_3$	39
**Figure S32**	^{13}C-NMR spectrum of 6-*O*-(4-fluorobutyl)-6-*O*-desmethyl-diprenorphine (**28c**, FB-DPN) in $CDCl_3$	40
**Figure S33**	^{19}F-NMR spectrum of 6-*O*-(4-fluorobutyl)-6-*O*-desmethyl-diprenorphine (**28c**, FB-DPN) in $CDCl_3$	41
**Figure S34**	^{1}H-NMR spectrum of 6-*O*-(5-fluoropentyl)-6-*O*-desmethyl-diprenorphine (**28d**, FPe-DPN) in $CDCl_3$	42
**Figure S35**	^{13}C-NMR spectrum of 6-*O*-(5-fluoropentyl)-6-*O*-desmethyl-diprenorphine (**28d**, FPe-DPN) in $CDCl_3$	43
**Figure S36**	^{19}F-NMR spectrum of 6-*O*-(5-fluoropentyl)-6-*O*-desmethyl-diprenorphine (**28d**, FPe-DPN) in $CDCl_3$	44
**Figure S37**	^{1}H-NMR spectrum of 6-*O*-(3-hydroxypropyl)-6-*O*-desmethyl-diprenorphine (**29b**, HP-DPN) in $CDCl_3$	45
**Figure S38**	^{13}C-NMR spectrum of 6-*O*-(3-hydroxypropyl)-6-*O*-desmethyl-diprenorphine (**29b**, HP-DPN) in $CDCl_3$	46
**Figure S39**	^{1}H-NMR spectrum of 6-*O*-(4-hydroxybutyl)-6-*O*-desmethyl-diprenorphine (**29c**, HB-DPN) in $CDCl_3$	47
**Figure S40**	^{13}C-NMR spectrum of 6-*O*-(4-hydroxybutyl)-6-*O*-desmethyl-diprenorphine (**29c**, HB-DPN) in $CDCl_3$	48
**Figure S41**	^{1}H-NMR spectrum of 6-*O*-(5-hydroxypentyl)-6-*O*-desmethyl-diprenorphine (**29d**, HPe-DPN) in $CDCl_3$	49
**Figure S42**	^{13}C-NMR spectrum of 6-*O*-(5-hydroxypentyl)-6-*O*-desmethyl-diprenorphine (**29d**, HPe-DPN) in $CDCl_3$	50
**Figure S43**	^{1}H-NMR spectrum of 3-fluoropropyl tosylate (**33a**, FPOTos) in $CDCl_3$	51
**Figure S44**	^{13}C-NMR spectrum of 3-fluoropropyl tosylate (**33a**, FPOTos) in $CDCl_3$	52
**Figure S45**	^{19}F-NMR spectrum of 3-fluoropropyl tosylate (**33a**, FPOTos) in $CDCl_3$	53
**Figure S46**	^{1}H-NMR spectrum of 4-fluorobutyl tosylate (**33b**, FBOTos) in $CDCl_3$	54
**Figure S47**	^{13}C-NMR spectrum of 4-fluorobutyl tosylate (**33b**, FBOTos) in $CDCl_3$	55
**Figure S48**	^{19}F-NMR spectrum of 4-fluorobutyl tosylate (**33b**, FBOTos) in $CDCl_3$	56
**Figure S49**	^{1}H-NMR spectrum of 5-fluoropentyl tosylate (**33c**, FPeOTos) in $CDCl_3$	57
**Figure S50**	^{13}C-NMR spectrum of 5-fluoropentyl tosylate (**33c**, FPeOTos) in $CDCl_3$	58
**Figure S51**	^{19}F-NMR spectrum of 5-fluoropentyl tosylate (**33c**, FPeOTos) in $CDCl_3$	59
**Figure S52**	^{1}H-NMR spectrum of (3-Bromo-propoxy)-*tert*-butyl-diphenyl-silane (**34a**, $Br(CH_2)_3OTBDPS$)	60
**Figure S53**	^{13}C-NMR spectrum of (3-Bromo-propoxy)-*tert*-butyl-diphenyl-silane (**34a**, $Br(CH_2)_3OTBDPS$)	61
**Figure S54**	^{1}H-NMR spectrum of (4-Bromo-butoxy)-*tert*-butyl-diphenyl-silane (**34b**, $Br(CH_2)_4OTBDPS$)	62
**Figure S55**	^{13}C-NMR spectrum of (4-Bromo-butoxy)-*tert*-butyl-diphenyl-silane (**34b**, $Br(CH_2)_4OTBDPS$)	63
**Figure S56**	^{1}H-NMR spectrum of (5-Bromo-pentyloxy)-*tert*-butyl-diphenyl-silane (**34c**, $Br(CH_2)_5OTBDPS$)	64
**Figure S57**	^{13}C-NMR spectrum of (5-Bromo-pentyloxy)-*tert*-butyl-diphenyl-silane (**34c**, $Br(CH_2)_5OTBDPS$)	65

High Resolution Mass Spectra for compunds 26b–d, 28b–d, and 29b–d

**Table S3**	High resolution mass spectra measurements	66
**Figure S58**	High Resolution Mass Spectrum of 6-*O*-(3-tosyloxypropyl)-6-*O*-desmethyl-3-*O*-trityl-diprenorphine (**26b**, TP-TDDPN)	67
**Figure S59**	High Resolution Mass Spectrum of 6-*O*-(4-tosyloxybutyl)-6-*O*-desmethyl-3-*O*-trityl-diprenorphine (**26c**, TB-TDDPN)	68
**Figure S60**		69
**Figure S61**	High Resolution Mass Spectrum of 6-*O*-(3-fluoropropyl)-6-*O*-desmethyl-diprenorphine (**28b**, FP-DPN)	70
**Figure S62**	High Resolution Mass Spectrum of 6-*O*-(4-fluorobutyl)-6-*O*-desmethyl-diprenorphine (**28c**, FB-DPN)	71
**Figure S63**	High Resolution Mass Spectrum of 6-*O*-(5-fluoropentyl)-6-*O*-desmethyl-diprenorphine (**28d**, FPe-DPN)	72
**Figure S64**	High Resolution Mass Spectrum of 6-*O*-(3-hydroxypropyl)-6-*O*-desmethyl-diprenorphine (**29b**, HP-DPN)	73
**Figure S65**	High Resolution Mass Spectrum of 6-*O*-(4-hydroxybutyl)-6-*O*-desmethyl-diprenorphine (**29c**, HB-DPN)	74
**Figure S66**	High Resolution Mass Spectrum of 6-*O*-(5-hydroxypentyl)-6-*O*-desmethyl-diprenorphine (**29d**, HPe-DPN)	75

ABBREVIATIONS

Compound, Term, Acronym	Comp.	Name, Synonyms
**AD**	-	Alzheimer's disease
**BPN**	-	buprenorphine, CAS RN: [52485-79-7]
**[^{11}C]BPN**	8	[^{11}C]buprenorphine
**BrCN**	-	cyanogen bromide, CAS RN: [506-68-3]
**[^{11}C]Caf**	1	[^{11}C]carfentanil
**CPM**	-	cyclopropylmethyl group
**cProp**	-	cyclopropyl group
**cyF**	-	cyclofoxy; 6-deoxy-6β-fluoro-noroxymorphone; 17-cyclopropylmethyl-4,5α-epoxy-6β-fluoro-morphinan-3,14-diol; CAS-RN: [103233-57-0]
**DBPN**	-	6-*O*-desmethyl-buprenorphine
**DDPN**	22	6-*O*-desmethyl-diprenorphine
**DDHE**	-	6-*O*-desmethyl-dihydroetorphine
**DHE**	-	dihydroetorphine
**DPEO**	-	6-*O*-desmethyl-phenethylorvinol
**DEAD**	-	diethyl azodicarboxylate, CAS RN: [1972-28-7]
**dihydrothevinone**	15	4,5α-epoxy-18,19-dihydro-17-methyl-3,6-dimethoxy-7α-acetyl-6,14-ethenomorphinan, CAS RN: [16196-82-0]
**DIPEA**	-	ethyldiisopropylamine; *N*,*N*-diisopropylethylamine; Hünig's base
**DMAP**	-	
**DMF**	-	*N*,*N*-dimethylformamide; CAS RN: [68-12-2]
**δ-OR**	-	δ-opioid receptor
**DPN**	-	diprenorphine, Revivon, M5050, CAS RN: [14357-78-9]
**[^{11}C]DPN**	-	[^{11}C]diprenorphine
**EOPs**	-	endogenous opioid peptides
**[^{18}F]FcyF**	-	[^{18}F]cyclofoxy, 6-deoxy-6*β*-[^{18}F]fluoro-naltrexone, CAS RN: [103223-58-1]
**FE-DPN**	28a	6-*O*-(2-fluoroethyl)-6-*O*-desmethyl-diprenorphine
**FP-DPN**	28b	6-*O*-(3-fluoropropyl)-6-*O*-desmethyl-diprenorphine
**FB-DPN**	28c	6-*O*-(4-fluorobutyl)-6-*O*-desmethyl-diprenorphine
**FPe-DPN**	28d	6-*O*-(5-fluoropentyl)-6-*O*-desmethyl-diprenorphine
**FE-TDDPN**	27a	6-*O*-(2-fluoroethyl)-6-*O*-desmethyl-3-*O*-trityl-diprenorphine
**FP-TDDPN**	27b	6-*O*-(3-fluoropropyl)-6-*O*-desmethyl-3-*O*-trityl-diprenorphine
**FB-TDDPN**	27c	6-*O*-(4-fluorobutyl)-6-*O*-desmethyl-3-*O*-trityl-diprenorphine
**FPe-TDDPN**	27d	6-*O*-(5-fluoropentyl)-6-*O*-desmethyl-3-*O*-trityl-diprenorphine
**FPOTos**	33a	3-fluoro-1-(toluene-*p*-sulfonyloxy)propane; CAS RN: [312-68-5]
**FBOTos**	33b	4-fluoro-1-(toluene-*p*-sulfonyloxy)butane; CAS RN: [433-10-3]
**FFeOTos**	33c	5-fluoro-1-(toluene-p-sulfonyloxy)pentane; CAS RN: [565-44-6]
**[^{18}F]FE-BPN**	11	6-*O*-(2-[^{18}F]fluoroethyl)-6-*O*-desmethyl-buprenorphine
**[^{18}F]FE-DPN**	10	6-*O*-(2-[^{18}F]fluoroethyl)-6-*O*-desmethyl-diprenorphine
**[^{18}F]FE-PEO**	12	6-*O*-(2-[^{18}F]fluoroethyl)-6-*O*-desmethyl-phenethylorvinol
**[^{18}F]FE-NTI**	4	*N*1'-(2-[^{18}F]fluoroethyl)naltrindole; BU97001
**GABA**	-	γ-aminobutyric acid; CAS-RN: [56-12-2]
**GPCR**	-	G-protein coupled receptor system
**[^{11}C]GR103545**	5	4-[(3,4-dichlorophenyl)acetyl]-3-(1-pyrrolidinylmethyl)-1-piperazinecarboxylic acid methyl-^{11}C ester
**HE-TDDPN**	25a	6-*O*-(2-hydroxyethyl)-6-*O*-desmethyl-3-*O*-trityl-diprenorphine
**HP-TDDPN**	25b	6-*O*-(3-hydroxypropyl)-6-*O*-desmethyl-3-*O*-trityl-diprenorphine
**HB-TDDPN**	25c	6-*O*-(4-hydroxybutyl)-6-*O*-desmethyl-3-*O*-trityl-diprenorphine
**HPe-TDDPN**	25d	6-*O*-(5-hydroxypentyl)-6-*O*-desmethyl-3-*O*-trityl-diprenorphine
**HE-DPN**	29a	6-*O*-(2-hydroxyethyl)-6-*O*-desmethyl-diprenorphine
**HP-DPN**	29b	6-*O*-(3-hydroxypropyl)-6-*O*-desmethyl-diprenorphine
**HB-DPN**	29c	6-*O*-(4-hydroxybutyl)-6-*O*-desmethyl-diprenorphine
**HPe-DPN**	29d	6-*O*-(5-hydroxypentyl)-6-*O*-desmethyl-diprenorphine

Compound, Term, Acronym	Comp.	Name, Synonyms
**HRMS**	-	high resolution mass spectrometry
**[^{125}I]-*O*-IA-DPN**	-	6-*O*-([^{125}I]iodoallyl)-6-*O*-desmethyl-diprenorphine
**κ-OR**	-	κ-opioid receptor
**K_i**	-	inhibition constant
**MeMgI**	-	methylmagnesium iodide
**[^{11}C]MeNTI**	**3**	*N*1′-[^{11}C]methyl-naltrindole
**μ-OR**	**-**	μ-opioid receptor
**NOP**	**-**	nociceptin/orphanin receptor
**[^{11}C]NOP-1A**	**6**	(2*S*)-2-[(2-fluorophenyl)methyl]-3-(2-fluorospiro[4,4-dihydro-thieno[2,3-c]pyran-7,4′-piperidine[1′-yl)-*N*-[^{11}C]methyl-propanamide
**ORs**	**-**	opioid receptors
**[^{11}C]PEO**	**9**	[^{11}C]phenethyl orvinol
**PET**	**-**	positron emission tomography
**PEO**	**-**	phenethylorvinol
**SPECT**	**-**	single photon emission computer tomography
**TBDPS**	**-**	*tert*-butyldiphenylsilyl group
**TBDPSCl**	**-**	*tert*-butyldiphenylsilyl chloride
**TE-TDDPN**	**26a**	6-*O*-(2-tosyloxyethyl)-6-*O*-desmethyl-diprenorphine; «Henriksen precursor»
**TP-TDDPN**	**26b**	6-*O*-(3-tosyloxypropyl)-6-*O*-desmethyl-3-*O*-trityl-diprenorphine
**TB-TDDPN**	**26c**	6-*O*-(4-tosyloxybutyl)-6-*O*-desmethyl-3-*O*-trityl-diprenorphine
**TPe-TDDPN**	**26d**	6-*O*-(5-tosyloxypentyl)-6-*O*-desmethyl-3-*O*-trityl-diprenorphine
**TDDPN**	**23**	3-*O*-trityl-6-*O*-desmethyl-diprenorphine; «Luthra precursor»
**thevinone**	**14**	4,5*α*-epoxy-17-methyl-3,6-dimethoxy-7*α*-acetyl-6,14-ethenomorphinan, CAS RN: [15358-22-2]
**Tos**	**-**	tosyl group
**Tr**	**-**	trityl group, triphenylmethy group

TABLE S1 ^{1}H and ^{13}C NMR chemical shifts and coupling constants of FP-TDDPN (**27b**), FB-TDDPN (**27c**) and FPe-TDDPN (**27d**)[a] in $CDCl_3$

**Position**	**FP-TDDPN (27b)**		**FB-TDDPN (27c)**		**FPe-TDDPN (27d)**	
	**δ ^{13}C**	**^{1}H** (m, *J* in Hz)	**δ ^{13}C**	**^{1}H** (m, *J* in Hz)	**δ ^{13}C**	**^{1}H** (m, *J* in Hz)
1	123.0	6.20 (d, 8.4)	122.9	6.19 (d, 8.0)	122.9	6.18 (d, 8.0)
2	117.9	6.49 (d, 8.1)	117.9	6.47 (d, 8.0)	117.8	6.47 (d, 8.0)
3	137.2	-	137.2	-	137.2	-
4	151.3	-	151.2	-	151.3	-
5β	96.5	4.10 (d, 1.9)	96.6	4.11 (d, 2.0)	96.7	4.11 (d, 1.7)
6	80.6	-	80.4	-	80.3	-
7β	48.1	1.84 (m)	48.0	1.83 (m)	48.0	1.83 (app t, 10.0)
8α	32.1	0.98 (dd, 13.3, 9.7)	32.1	0.98 (dd, 13.6, 9.7)	32.1	0.98 (dd, 13.1, 9.6)
8β		2.77 (ddd, 13.4, 11.7, 3.7)		2.77 (ddd, 13.6, 9.9, 3.8)		2.77 (ddd, 13.1, 12.1, 3.7)
9α	57.9	2.90 (d, 6.3)	58.0	2.90 (d, 6.3)	58.0	2.90 (d, 6.4)
10α	22.5	2.05 (dd, 18.3, 6.3)	22.5	2.05 (dd, 18.5, 6.3)	22.5	2.05 (dd, 18.4, 6.4)
10β		2.86 (d, 18.3)		2.86 (d, 18.5)		2.85 (d, 18.4)
11	130.7	-	130.6	-	130.7	-
12	131.9	-	132.0	-	132.0	-
13	46.8	-	46.7	-	46.7	-
14	35.7	-	35.7	-	35.7	-
15_{ax}	35.4	1.55–1.60 (m)	35.4	1.90 (td, 12.8, 5.5)	35.4	1.90 (td, 12.7, 5.5)
15_{eq}		1.43 (dd, 13.0, 3.7)		1.44 (dd, 13.2, 2.6)		1.37–1.70 (m[n])
16_{ax}	43.6	2.15 (td, 11.7, 3.7)	43.6	2.15 (td, 10.4, 3.7)	43.6	2.15 (td, 13.6, 3.5)
16_{eq}		2.55 (dd, 11.7, 4.9)		2.55 (dd, 12.0, 4.7)		2.55 (dd, 11.9, 5.0)
18_{syn}	17.5	1.38 (td, 13.9, 5.3)	17.7	1.40 (td, 13.2, 5.4)	17.7	1.37–1.70 (m[n])
18_{anti}		1.85–1.92 (m[e])		1.57–1.72 (m[j])		1.37–1.70 (m[n])
19_{syn}	29.8	0.42 (m)	29.7	0.43 (m)	29.8	0.41 (m)
19_{anti}		0.90 (td, 12.6, 5.8)		0.90 (td, 12.6, 5.7)		0.90 (td, 12.5, 5.8)
20	74.2	-	74.2	-	74.2-	
**Others**						
20-CH_3	24.8	1.16 (s)	24.9	1.16 (s)	24.8	1.16 (s)
20-CH_3	29.7	1.35 (s)	29.8	1.35 (s)	29.7	1.35 (s)
cPropCH_{2syn}	3.2	0.06 (m)	3.2	0.06 (m)	3.2	0.06 (m)
cPropCH_{2anti}	4.1	0.46 (m)	4.1	0.46 (m)	4.1	0.46 (m)
cProp*CH*	9.3	0.76 (m)	9.3	0.76 (m)	9.3	0.76 (m)
NCH_2 (a)	59.8	2.19 (dd, 12.6, 6.8)	59.8	2.18 (dd, 12.9, 6.7)	59.8	2.18 (dd, 12.7, 6.7)
NCH_2 (b)		2.32 (dd, 12.7, 5.7)		2.33 (dd, 12.9, 5.8)		2.32 (dd, 12.7, 5.7)
Trityl (Tr)						
Tr (*m,p*)		7.20–7.26 (m)	-	7.21–7.24 (m)		7.20–7.25 (m)
Tr (*o*)		7.40–7.45 (m)	-	7.41–7.44 (m)		7.41–7.45 (m)
Ph_3CO	91.4	-	91.4	-	91.4	
*p*CTr	127.2	-	127.2	-	127.2	
*m*CTr	127.3	-	127.3	-	127.3	
*o*CTr	129.4	-	129.3	-	129.4	
TrC1	144.2	-	144.2	-	144.2	
20-OH	-	5.08 (br s)	-	5.21 (br s)	-	5.27 (br s)
6-*O*-CH_2*CH_2*CH_2F (b,b’)	31.7d[b]	1.85–1.92 (m)[e]	-	-	-	-
6-*O*-*CH_2*CH_2CH_2F (a)	61.0d[c]	3.60 (dt, 9.5, 6.5)	-	-	-	-
6-*O*-*CH_2*CH_2CH_2F (a’)		3.89 (dt, 9.6, 5.9)	-	-	-	-
6-*O*-CH_2CH_2*CH_2*F (c,c’)	81.2d[d]	4.42–4.51 (m)	-	-	-	-
6-*O*-*CH_2*$CH_2CH_2CH_2$F (a,a’)	-	-	64.2 d[f]	3.48 (dt, 9.5, 6.5)	-	-
	-	-		3.83 (dt, 9.6, 5.9)	-	-
6-*O*-CH_2*CH_2*CH_2CH_2F (b,b’)	-	-	26.4 d[g]	1.57–1.72 (m[j])	-	-
6-*O*-CH_2CH_2*CH_2*CH_2F (c,c’)	-	-	27.2 d[h]	1.57–1.72 (m[j])	-	-
6-*O*-$CH_2CH_2CH_2$*CH_2*F (d,d’)	-	-	83.7 d[i]	4.35–4.48 (m)	-	-
6-*O*-*CH_2*$CH_2CH_2CH_2CH_2$F (a)	-	-	-	-	64.5	3.45 (dt, 9.1, 6.3)
6-*O*-*CH_2*$CH_2CH_2CH_2CH_2$F (a’)	-	-	-	-		3.81 (dt, 9.1, 6.3)
6-*O*-CH_2*CH_2*$CH_2CH_2CH_2$F (b,b’)	-	-	-	-	21.8 d[k]	1.37–1.70 (m)
6-*O*-CH_2CH_2*CH_2*CH_2CH_2F (c,c’)	-	-	-	-	30.3	1.37–1.70 (m)
6-*O*-$CH_2CH_2CH_2$*CH_2*CH_2F (d,d’)	-	-	-	-	30.1 d[l]	1.37–1.70 (m)
6-*O*-$CH_2CH_2CH_2CH_2$*CH_2*F (e)	-	-	-	-	83.9 d[m]	4.35 (t, 6.1)
6-*O*-$CH_2CH_2CH_2CH_2$*CH_2*F (e’)	-	-	-	-		4.45 (t, 6.1)

a: observation frequency: 500.130 MHz (for ^{1}H-NMR), 125.758 MHz (for ^{13}C-NMR), in $CDCl_3$, δ in ppm; b: $^{2}J_{C,F}$ = 20.2 Hz; c: $^{3}J_{C,F}$ = 5.5 Hz; d: : $^{1}J_{C,F}$ = 165.9 Hz; e: overlapping multiplets of H-18_{anti} and 6-*O*-CH_2*CH_2*CH_2F (b,b'); f: $^{4}J_{C,F}$ = 1.9 Hz; g: $^{3}J_{C,F}$ = 4.6 Hz, h: $^{2}J_{C,F}$ = 20.2 Hz; i: $^{1}J_{C,F}$ = 165.1 Hz; j: overlapping signals of H-18_{anti}, 6-*O*-CH_2*CH_2*CH_2CH_2F (b,b') and 6-O-CH_2*CH_2*CH_2CH_2F (c,c'); k: $^{3}J_{C,F}$ = 5.5 Hz; l: $^{2}J_{C,F}$ = 20.2 Hz; m: $^{1}J_{C,F}$ = 164.8 Hz; n: overlapping signals of H-15_{eq}, H-18_{syn} and H-18_{anti}

TABLE S2 ^{1}H and ^{13}C NMR chemical shifts and coupling constants of HP-DPN (**29b**), HB-DPN (**29c**) and HPe-DPN (**29d**)[a] in $CDCl_3$

Position	**HP-DPN (29b)**		**HB-DPN (29c)**		**HPe-DPN (29d)**	
	**δ ^{13}C**	**^{1}H** (m, *J* in Hz)	**δ ^{13}C**	**^{1}H** (m, *J* in Hz)	**δ ^{13}C**	**^{1}H** (m, *J* in Hz)
1	119.5	6.49 (d, 8.0)	119.6	6.50 (d, 8.1)	119.5	6.50 (d, 8.0)
2	116.8	6.69 (d, 8.0)	116.6	6.69 (d, 8.1)	116.7	6.68 (d, 8.0)
3	137.6	-	137.5	-	137.5	-
4	145.5	-	145.2	-	145.4	-
5β	97.4	4.39 (d, 1.4)	97.3	4.39 (d, 1.0)	97.5	4.38 (d, 1.7)
6	80.6	-	80.6	-	80.5	-
7β	48.0	1.93 (app t, 10.1)	48.0	1.93 (app t, 10.0)	48.0	1.94 (app t, 10.0)
8α	32.3	1.06 (dd, 13.4, 9.3)	32.3	1.07 (dd, 12.4, 9.3)	32.3	1.07 (dd, 13.7, 9.7)
8β		2.85 (ddd, 13.5, 12.3, 3.7)		2.85 (ddd, 13.5, 11.9, 3.7)		2.85 (ddd, 13.5, 12.2, 3.7)
9α	58.2	3.00 (d, 6.4)	58.2	3.00 (d, 6.7)	58.2	3.00 (d, 6.3)
10α	22.6	2.17–2.29 (m[b])	22.6	2.17–2.30 (m[e])	22.6	2.20 (dd, 18.4, 6.3)
10β		2.96 (d, 18.4)		2.97 (d, 18.4)		2.97 (d, 18.4)
11	127.7	-	127.9	-	127.9	-
12	132.2	-	132.3	-	132.3	-
13	47.0	-	47.1	-	47.0	-
14	35.9	-	35.9	-	35.9	-
15_{ax}	35.4	2.01 (td, 13.6, 5.7)	35.5	2.02 (td, 13.6, 5.7)	35.5	2.03 (td, 13.6, 5.3)
15_{eq}		1.63 (dd, 13.1, 2.3)		1.58–1.66 (m[f])		1.62–1.87 (m[j])
16_{ax}	43.7	2.17–2.29 (m[b])	43.7	2.17–2.30 (m[e])	43.7	2.23–2.30 (m[k])
16_{eq}		2.61 (dd, 12.1, 5.0)		2.62 (dd, 11.9, 5.0)		2.62 (dd, 12.0, 5.0)
18_{syn}	18.0	1.75–1.85 (m[c])	18.1	1.77–1.82 (m[f])	17.9	1.42–1.59 (m[l])
18_{anti}		1.75–1.85 (m[c])		1.69–1.79 (m[g])		1.62–1.87 (m[j])
19_{syn}	29.9	0.73 (m)	29.8	0.72–0.81 (m[h])	29.7	0.75 (m)
19_{anti}		1.01 (m)		1.02 (m)		1.02 (m)
20	74.7	-	74.5	-	74.6	-
**Others**						
20-*CH_3*	24.9	1.20 (s)	24.9	1.20 (s)	24.9	1.20 (s)
20-*CH_3*	29.7	1.39 (s)	29.9	1.39 (s)	29.9	1.39 (s)
cProp*CH_{2syn}*	3.3	0.09 (m)	3.3	0.09 (m)	3.3	0.09 (m)
cProp*CH_{2anti}*	4.1	0.49 (m)	4.2	0.49 (m)	4.1	0.49 (m)
cProp*CH*	9.4	0.79 (m)	9.4	0.72–0.81 (m[h])	9.4	0.78 (m)
N*CH_2* (a)	59.2	2.17–2.29 (m[b])	59.8	2.17–2.30 (m[e])	59.8	2.23–2.30 (m[k])
N*CH_2* (b)		2.36 (dd, 12.9, 5.7)		2.37 (dd, 12.6, 5.7)		2.37 (dd, 12.7, 5.8)
20-OH	-	5.34 (br s)	-	5.31 (br s)	-	5.38 (br s)
3-OH	-	6.76 (br s)		6.40 (br s)	-	6.16 (br s)
6-*O*-*CH_2*CH_2CH_2OH	59.8	3.69–4.17 (m[d])	-	-	-	-
6-*O*-CH_2*CH_2*CH_2OH	33.1	1.75–1.85 (m[c])	-	-	-	-
6-*O*-CH_2CH_2*CH_2*OH	61.4	3.69–4.17 (m[d])	-	-	-	-
6-*O*-*CH_2*$CH_2CH_2CH_2OH$	-	-	62.1	3.60–4.06 (m[i])	-	-
6-*O*-CH_2*CH_2*CH_2CH_2OH	-	-	26.9	1.69–1.79 (m[g])	-	-
6-*O*-CH_2CH_2*CH_2*CH_2OH	-	-	28.7	1.69–1.79 (m[g])	-	-
6-*O*-$CH_2CH_2CH_2$*CH_2*OH	-	-	63.9	3.60–4.06 (m[i])	-	-
6-*O*-*CH_2*$CH_2CH_2CH_2CH_2OH$	-	-	-	-	62.4	3.60–3.97 (m[m])
6-*O*-CH_2*CH_2*$CH_2CH_2CH_2OH$	-	-	-	-	30.0	1.62–1.87 (m[j])
6-*O*-CH_2CH_2*CH_2*CH_2CH_2OH	-	-	-	-	21.8	1.42–1.59 (m[l])
6-*O*-$CH_2CH_2CH_2$*CH_2*CH_2OH	-	-	-	-	32.3	1.62–1.87 (m[j])
6-*O*-$CH_2CH_2CH_2CH_2$*CH_2*OH	-	-	-	-	64.2	3.60–3.97 (m[m])

a: observation frequency: 500.130 MHz (for ^{1}H-NMR), 125.758 MHz (for ^{13}C-NMR), in $CDCl_3$, δ in ppm; b: overlapping multiplets of H-10α, H-16_{ax} and N*CH_2* (a); c: overlapping signals of H-18_{syn}, H-18_{anti} and 6-*O*-CH_2*CH_2*CH_2OH; d: overlapping multiplets of 6-*O*-*CH_2*CH_2CH_2OH and 6-*O*-CH_2CH_2*CH_2*OH; e: overlapping sinals of H-10a, H-16_{ax} and N*CH_2* (a); f: overlapping multiplets of H-10α 15_{eq}; g: overlapping signals of H-19_{anti}, 6-*O*-CH_2*CH_2*CH_2CH_2OH and 6-*O*-CH_2CH_2*CH_2*CH_2OH; h: overlapping multiplets of H-19_{syn} and cProp*CH*; i: overlapping multiplets of 6-*O*-*CH_2*$CH_2CH_2CH_2OH$ and 6-*O*-$CH_2CH_2CH_2$*CH_2*OH j: overlapping signals of H-15_{eq}, 18_{anti}, 6-*O*-CH_2*CH_2*$CH_2CH_2CH_2OH$ and 6-*O*-$CH_2CH_2CH_2$*CH_2*CH_2OH; k: overlapping signals of H-16_{ax} and N*CH_2* (a); l: overlapping signals of H-18_{syn} and 6-*O*-CH_2CH_2*CH_2*CH_2CH_2OH; m: overlapping multiplets of 6-*O*-*CH_2*$CH_2CH_2CH_2CH_2OH$ and 6-*O*-$CH_2CH_2CH_2CH_2$*CH_2*OH.

Table S3. Reaction conditions, yields, and physical constants for compounds prepared

Method	Educt	Product	Reagent	T [°C]	τ	m [mg]	Yield [°C]	mp [°C]
**3.2.1**								
	**23**	**24b**	**34a**	RT	20 h	470	40	98–102
	**23**	**24c**	**34b**	RT	20 h	419	71	77–88
	**23**	**24d**	**34c**	RT	20 h	573	61	70–81
**3.2.2**								
	**24b**	**25b**	1M Bu_4NF	RT	4 h	254	84	110–121
	**24c**	**25c**	1M Bu_4NF	RT	4 h	340	93	105–116
	**24d**	**25d**	1M Bu_4NF	RT	4 h	385	90	101–109
**3.2.3**								
	**25b**	**26b**	Tos_2O	RT	4 h	349	60	87–102
	**25c**	**26c**	Tos_2O	RT	4 h	337	87	80–98
	**25d**	**26d**	Tos_2O	RT	4 h	311	67	77–88
**3.2.4**								
	**23**	**27b**	**33a**	RT	20 h	310	43	80–105
	**23**	**27c**	**33b**	RT	20 h	440	60	93–102
	**23**	**27d**	**33c**	RT	20 h	487	66	82–92
**3.2.5**								
	**27b**	**28b**	AcOH[a]	RT	5 min	129	78	98–115
	**27c**	**28c**	AcOH[a]	RT	5 min	190	71	81–96
	**27d**	**28d**	AcOH[a]	RT	5 min	220	76	81–90
**3.2.6**								
	**25b**	**29b**	AcOH[b]	100	10 min	120	77	208–210
	**25c**	**29c**	AcOH[b]	100	10 min	145	77	209–210
	**25d**	**29d**	AcOH[b]	100	10 min	105	72	86–116

[a] 27:7 (v/v) acetic acid-water mixture, 100 °C; [b] 4:1 (v/v) acteic acid-water mixture

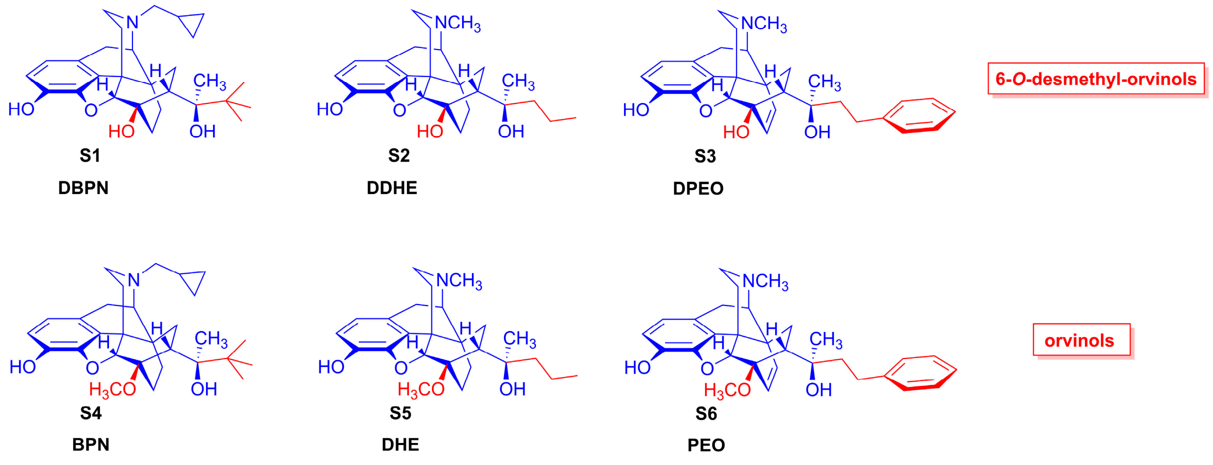

Chart S1. Chemical structures of selected orvinol an 6-*O*-desmethyl-orvinol derivatives

Figure S1 ^{1}H NMR spectrum of 6-*O*-(3-*tert*-butyldiphenylsilyloxypropyl)-6-*O*-desmethyl-3-*O*-trityl-diprenorphine (**24b**, TBDPS-OP-TDDPN) in $CDCl_3$

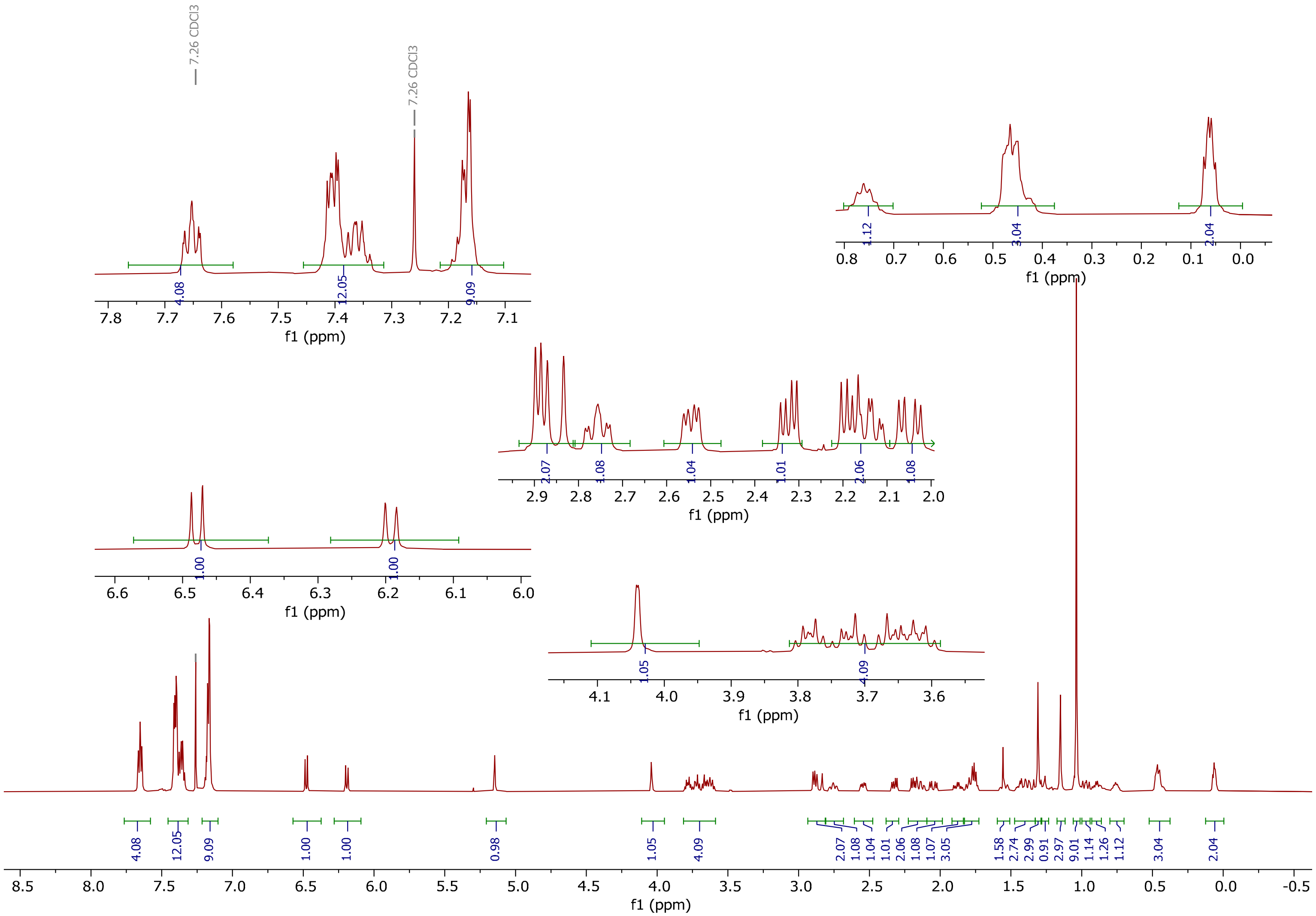

Figure S2 ^{13}C NMR spectrum of 6-*O*-(3-*tert*-butyldiphenylsilyloxypropyl)-6-*O*-desmethyl-3-*O*-trityl-diprenorphine (**24b**, TBDPS-OP-TDDPN) in $CDCl_3$

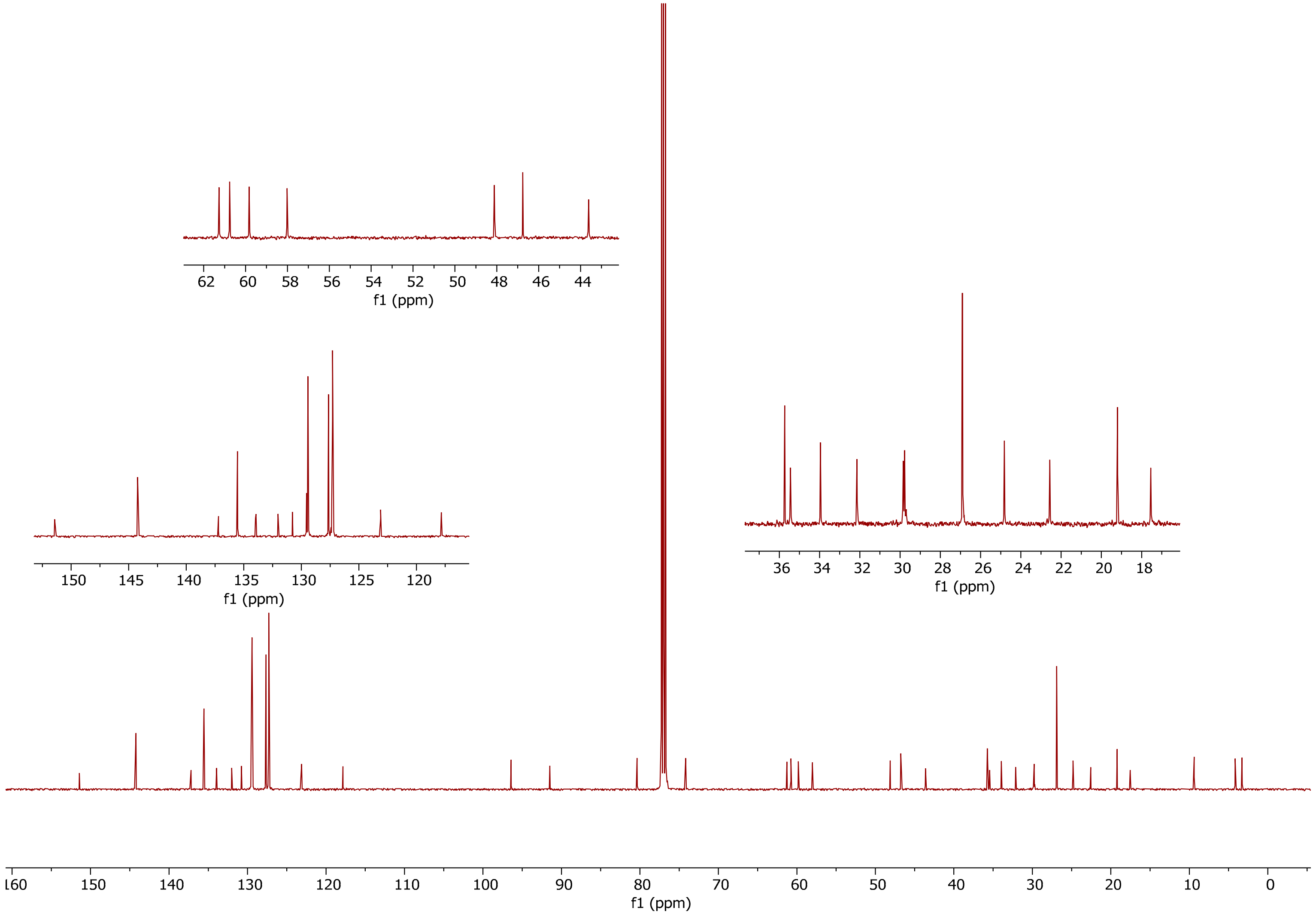

Figure S3 ^{1}H NMR spectrum of 6-*O*-(4-*tert*-butyldiphenylsilyloxybutyl)-6-*O*-desmethyl-3-*O*-trityl-diprenorphine (**24c**, TBDPS-OB-TDDPN) in $CDCl_3$

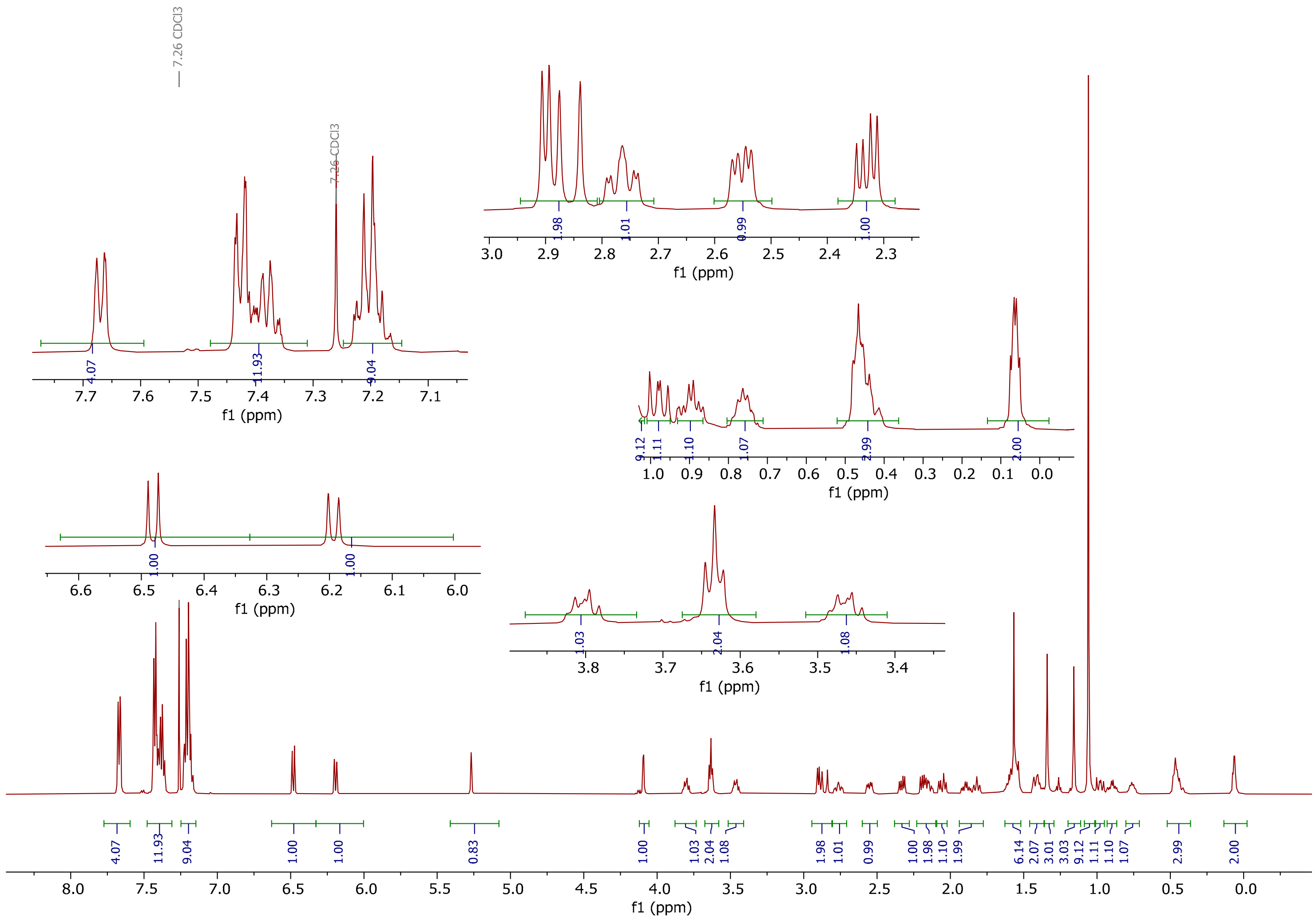

Figure S4 ^{13}C NMR spectrum of 6-*O*-(4-*tert*-butyldiphenylsilyloxybutyl)-6-*O*-desmethyl-3-*O*-trityl-diprenorphine (**24c**, TBDPS-OB-TDDPN) in $CDCl_3$

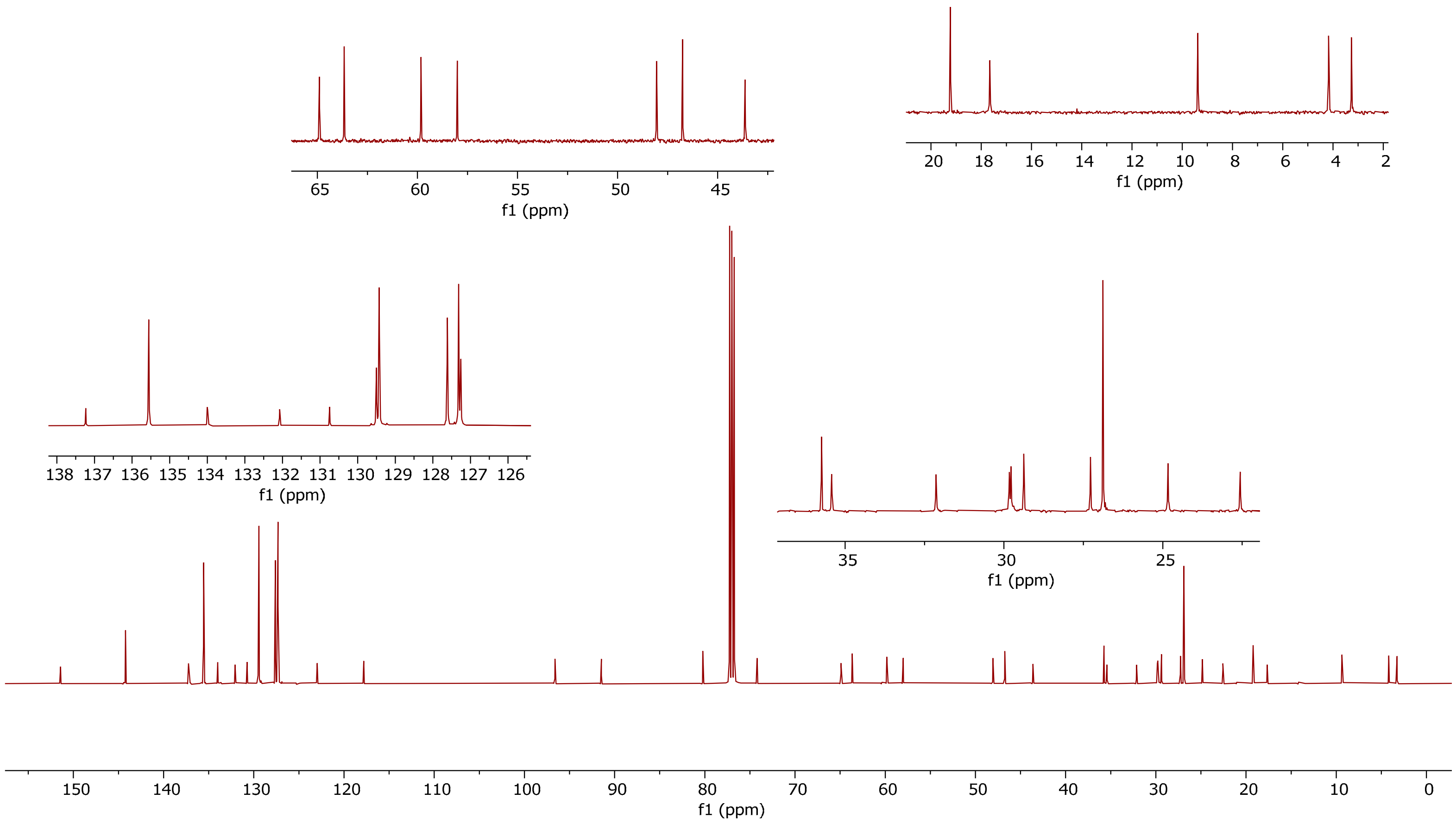

Figure S5 ^{1}H NMR spectrum of 6-*O*-(5-*tert*-butyldiphenylsilyloxypentyl)-6-*O*-desmethyl-3-*O*-trityl-diprenorphine (**24d**, TBDPS-OPe-TDDPN) in $CDCl_3$

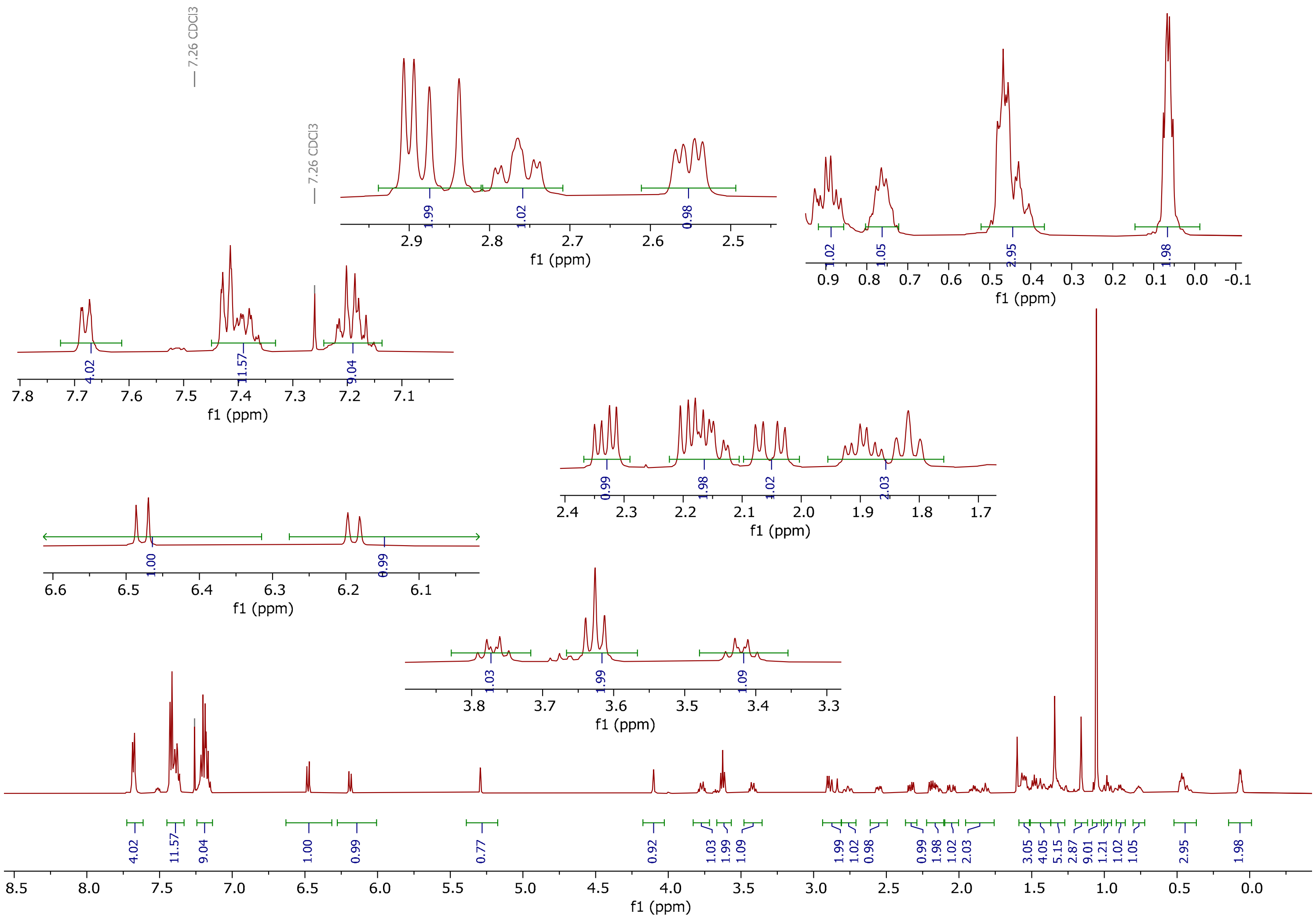

Figure S6 ^{13}C NMR spectrum of 6-*O*-(5-*tert*-butyldiphenylsilyloxypentyl)-6-*O*-desmethyl-3-*O*-trityl-diprenorphine (**24d**, TBDPS-OPe-TDDPN) in $CDCl_3$

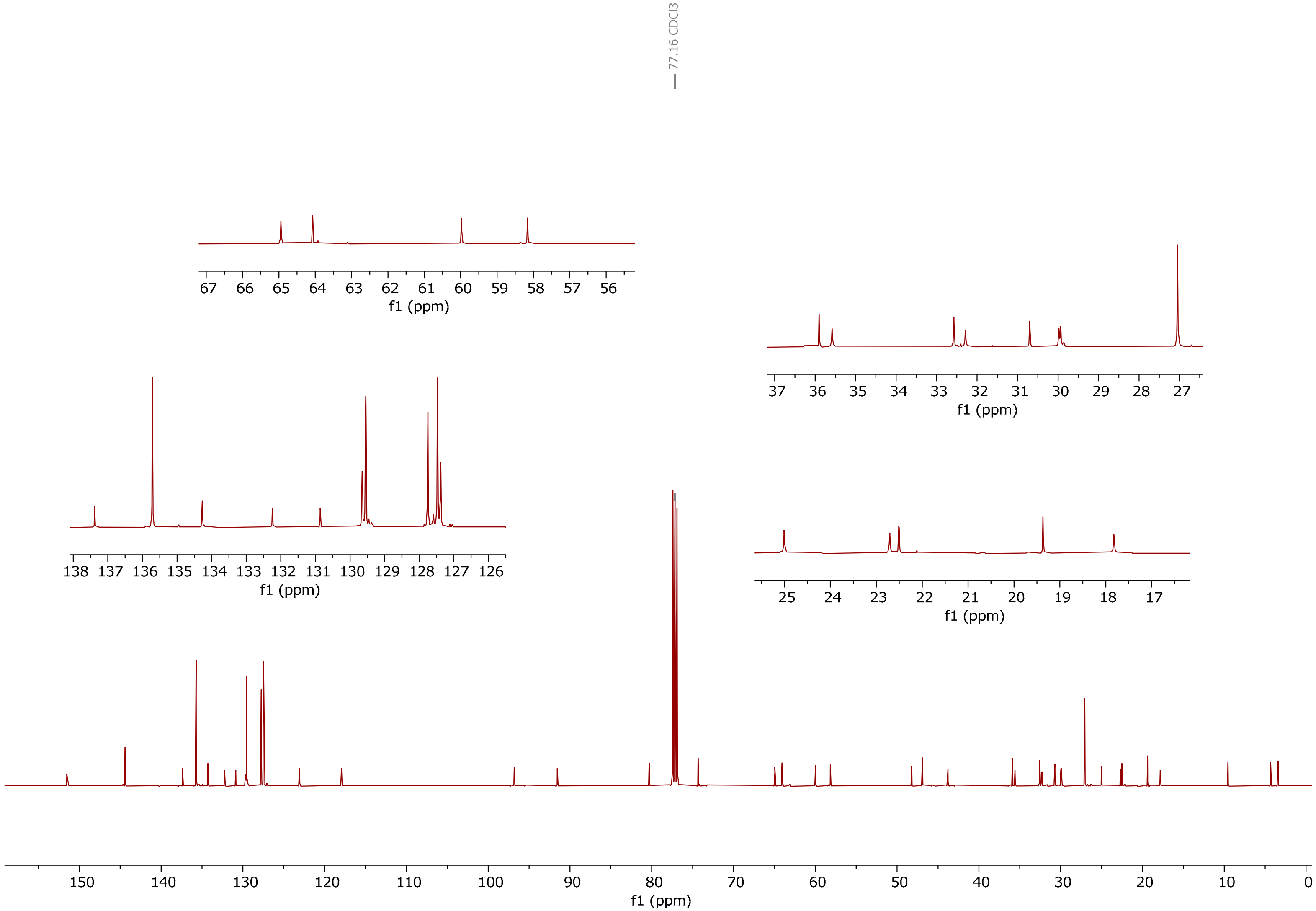

Figure S7 ^{1}H NMR spectrum of 6-*O*-(3-hydroxypropyl)-6-*O*-desmethyl-3-*O*-trityl-diprenorphine (**25b**, HP-TDDPN) in $CDCl_3$

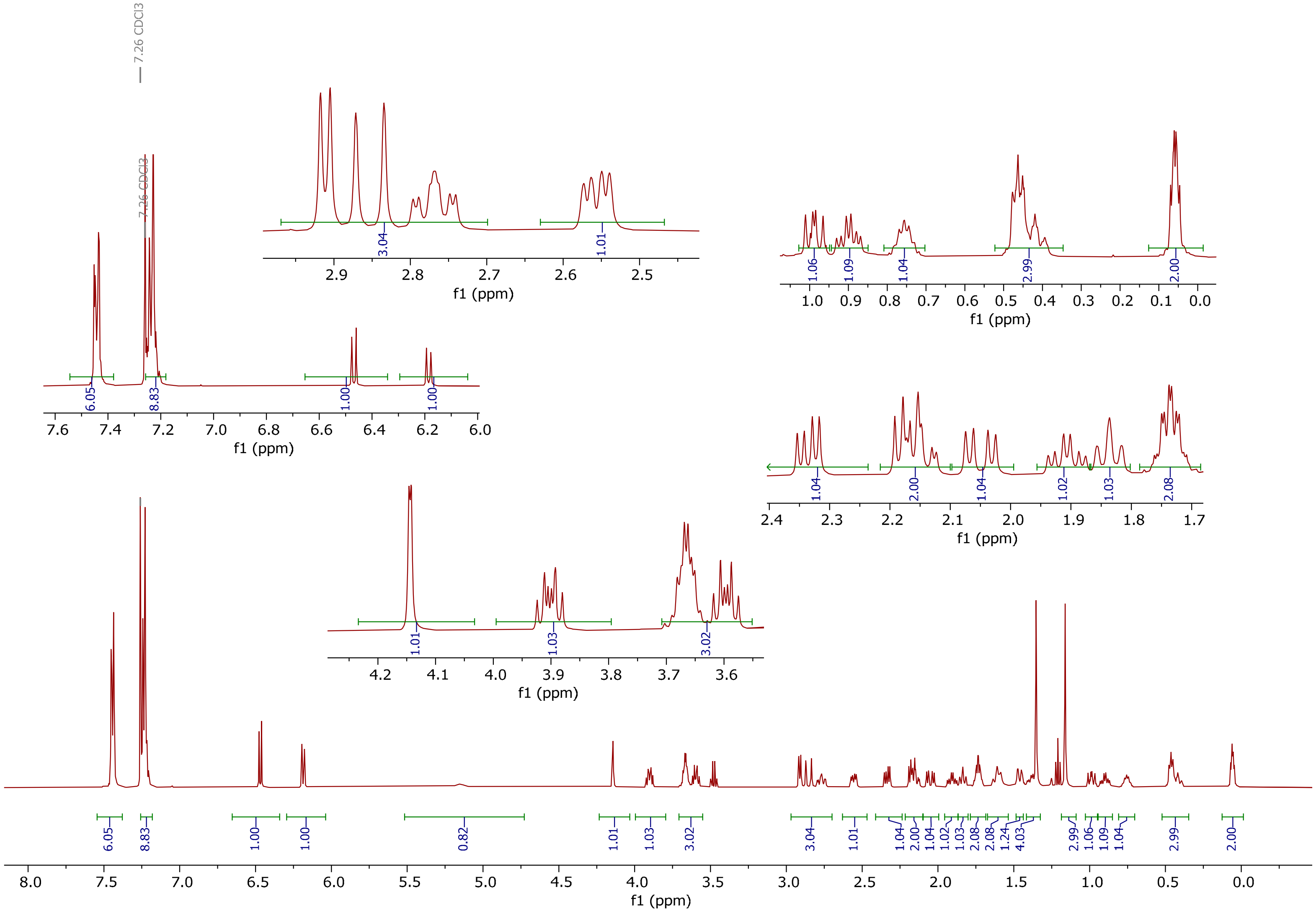

Figure S8 ^{13}C NMR spectrum of 6-*O*-(3-hydroxypropyl)-6-*O*-desmethyl-3-*O*-trityl-diprenorphine (**25b**, HP-TDDPN) in CDl_3

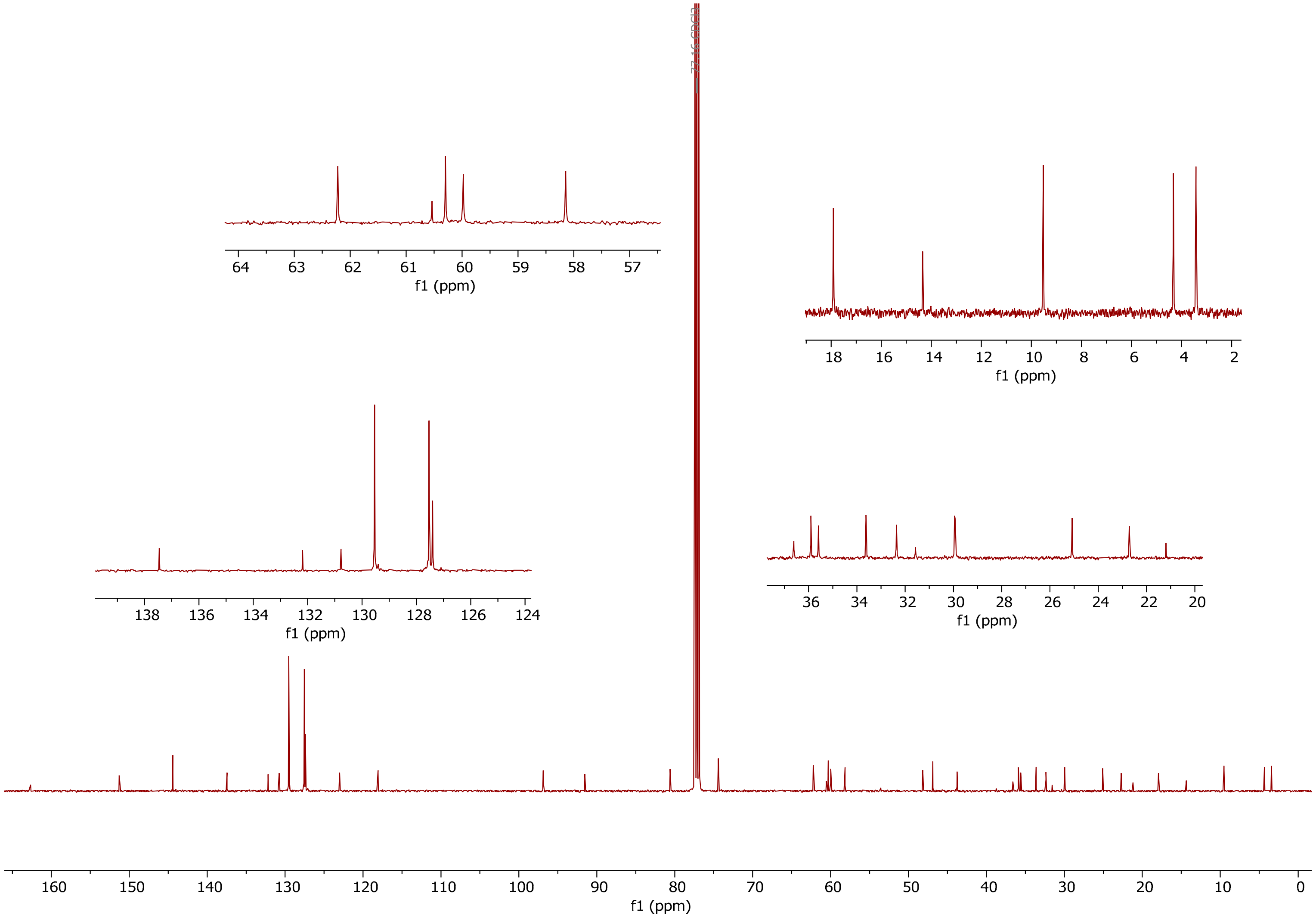

Figure S9 ^{1}H NMR spectrum of 6-*O*-(4-hydroxybutyl)-6-*O*-desmethyl-3-*O*-trityl-diprenorphine (**25c**, HB-TDDPN) in CDCl$_3$

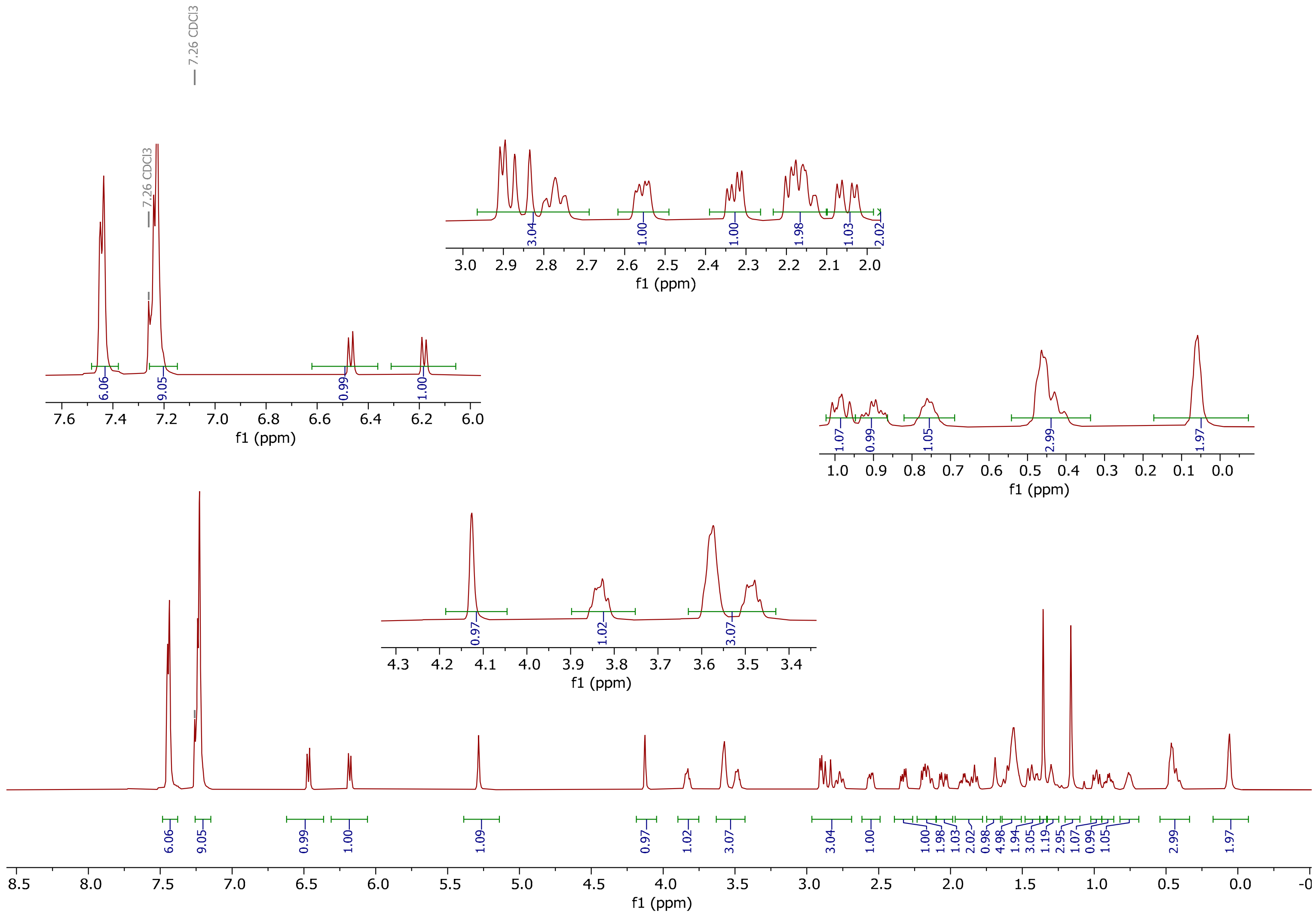

Figure S10 ^{13}C NMR spectrum of 6-*O*-(4-hydroxybutyl)-6-*O*-desmethyl-3-*O*-trityl-diprenorphine (**25c**, HB-TDDPN) in $CDCl_3$

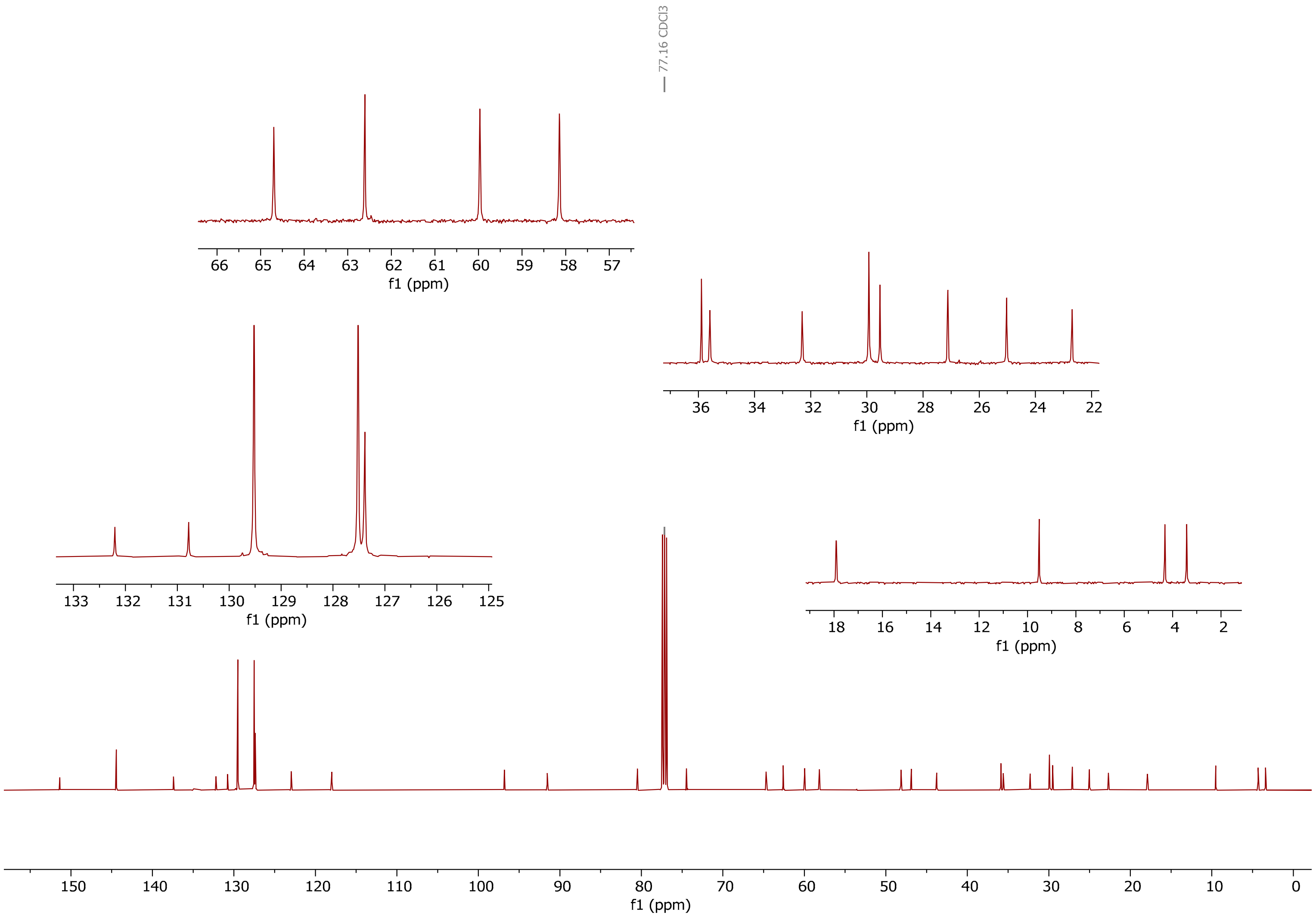

Figure S11 ^{1}H NMR spectrum of 6-*O*-(5-hydroxypentyl)-6-*O*-desmethyl-3-*O*-trityl-diprenorphine (**25d**, HPe-TDDPN)in $CDCl_3$

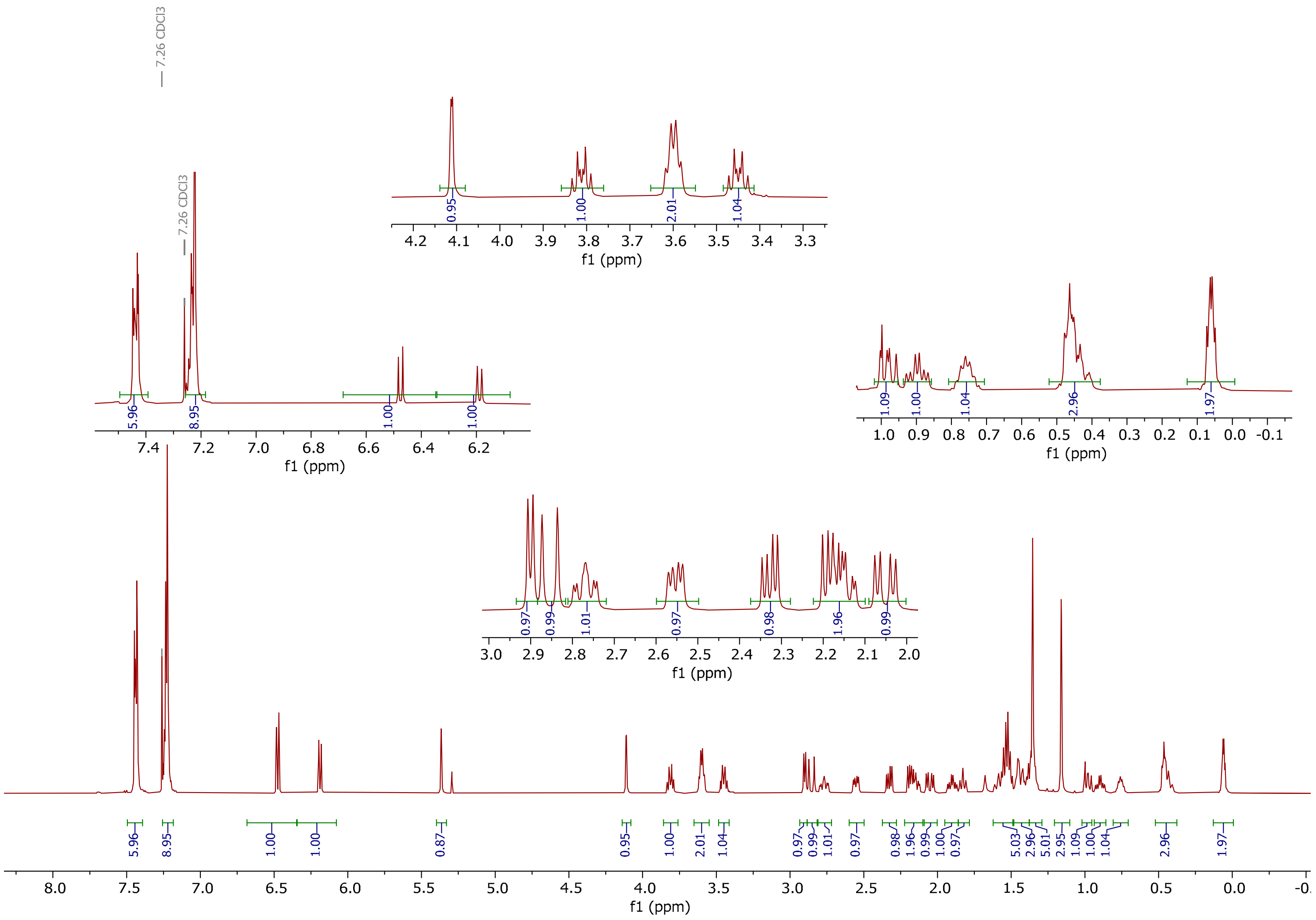

Figure S12 ^{13}C NMR spectrum of 6-*O*-(5-hydroxypentyl)-6-*O*-desmethyl-3-*O*-trityl-diprenorphine (**25d**, HPe-TDDPN) in $CDCl_3$

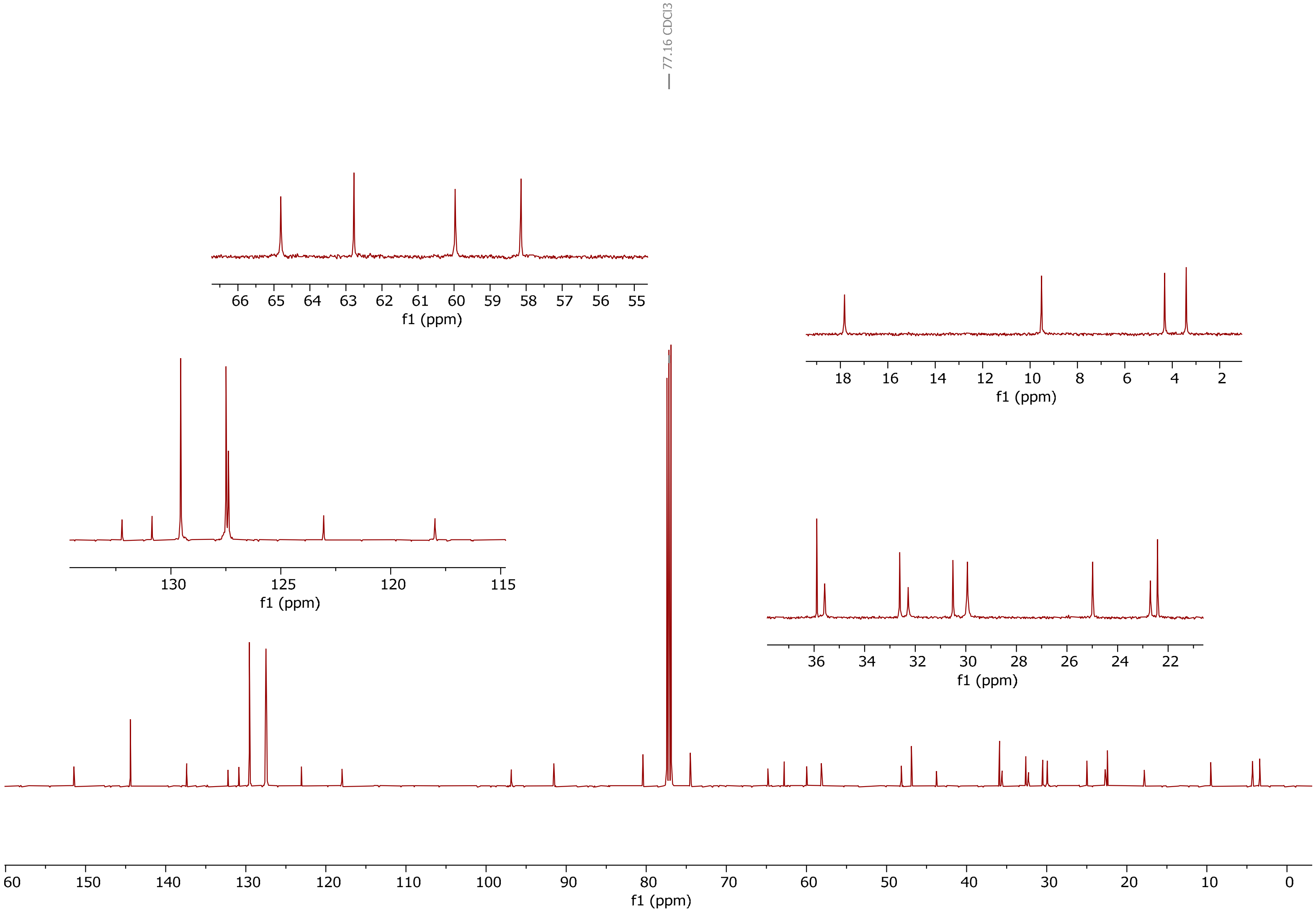

Figure S13 ^{1}H NMR spectrum of 6-*O*-(3-tosyloxypropyl)-6-*O*-desmethyl-3-*O*-trityl-diprenorphine (**26b**, TP-TDDPN) in $CDCl_3$

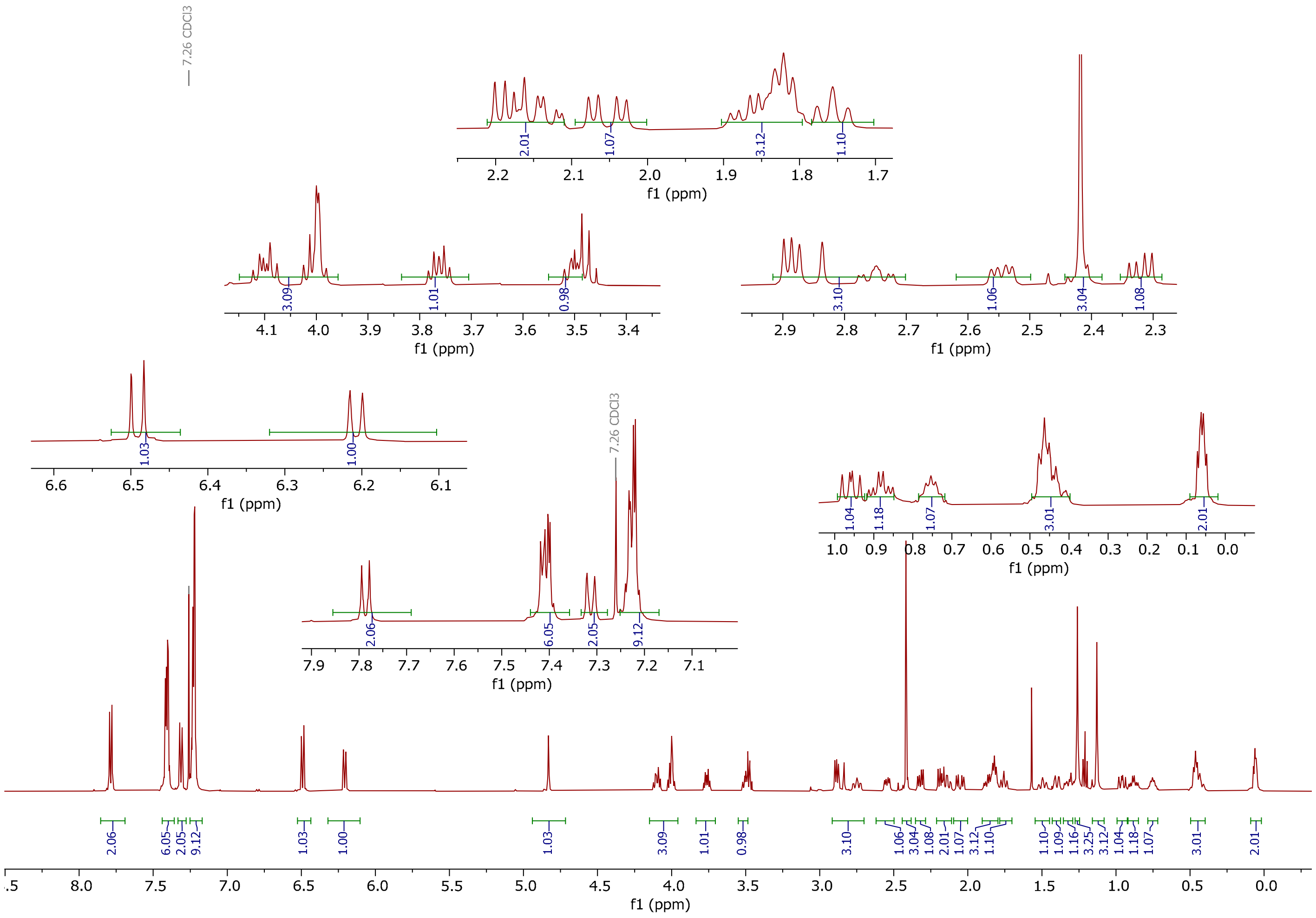

Figure S14 ^{1}H NMR spectrum of 6-*O*-(3-tosyloxypropyl)-6-*O*-desmethyl-3-*O*-trityl-diprenorphine (**26b**, TP-TDDPN) in $CDCl_3$

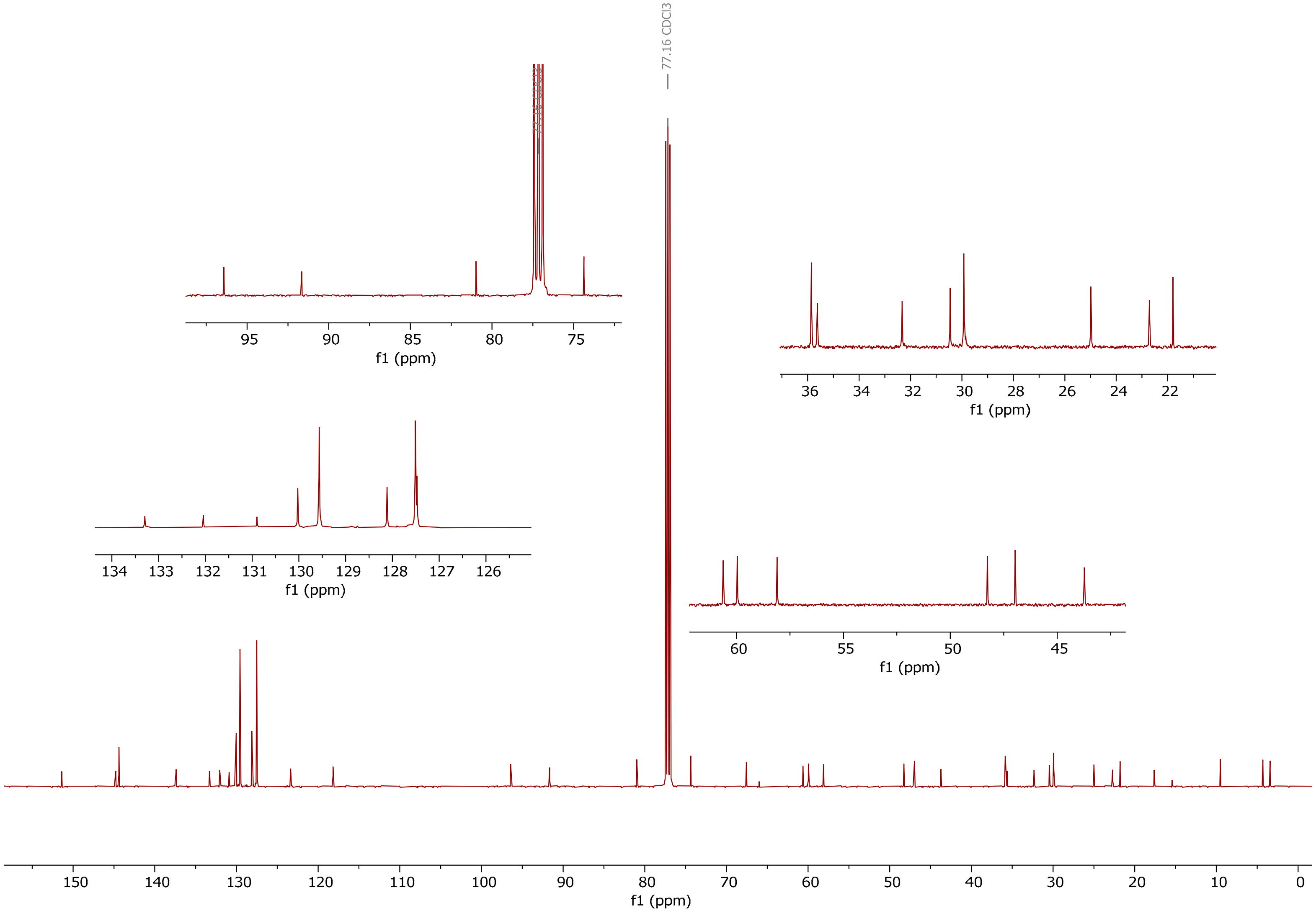

Figure S15 ^{1}H NMR spectrum of 6-*O*-(4-tosyloxybutyl)-6-*O*-desmethyl-3-*O*-trityl-diprenorphine (**26c**, TB-TDDPN) in $CDCl_3$

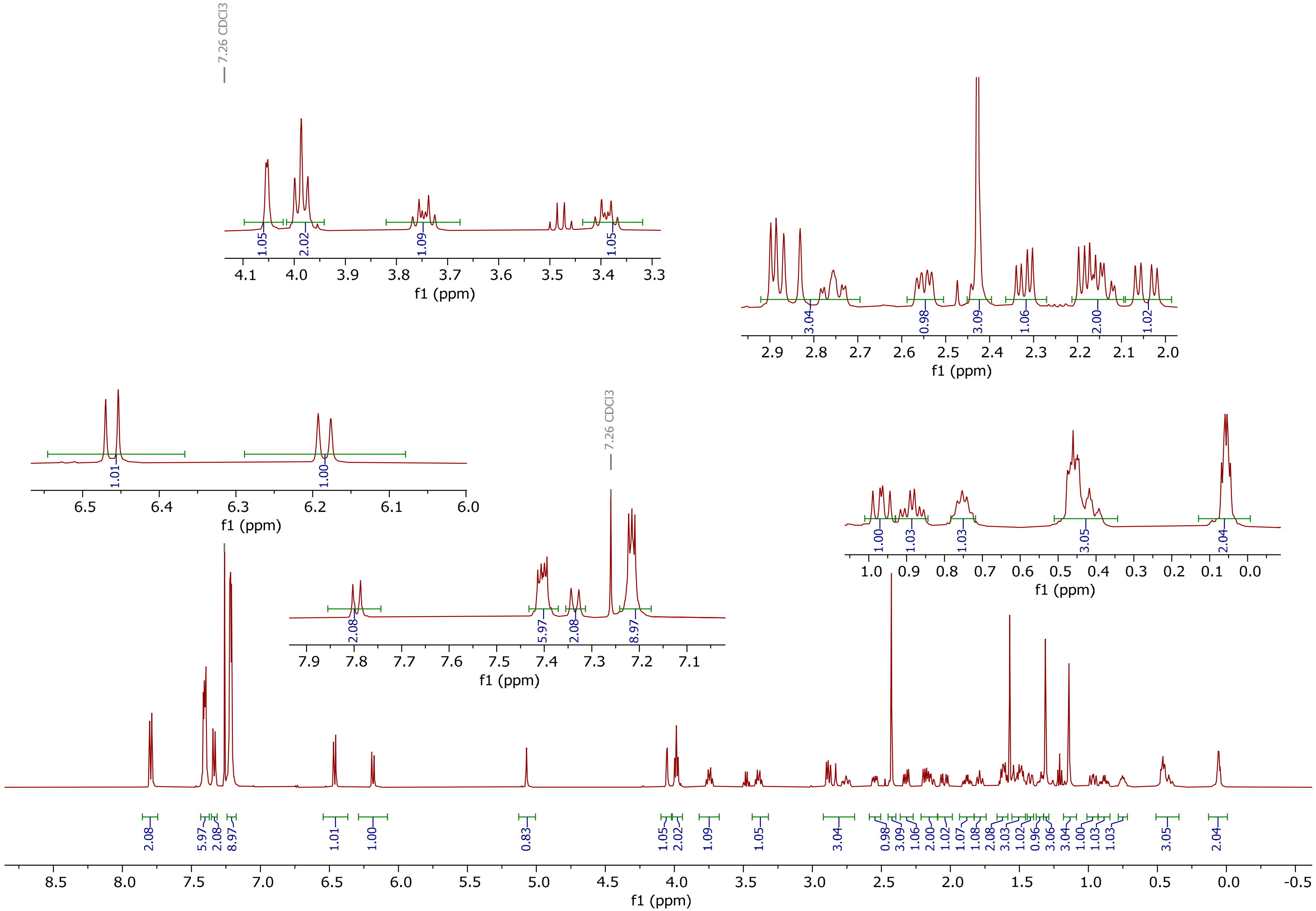

Figure S16 ^{13}C NMR spectrum of 6-*O*-(4-tosyloxybutyl)-6-*O*-desmethyl-3-*O*-trityl-diprenorphine (**26c**, TB-TDDPN) in $CDCl_3$

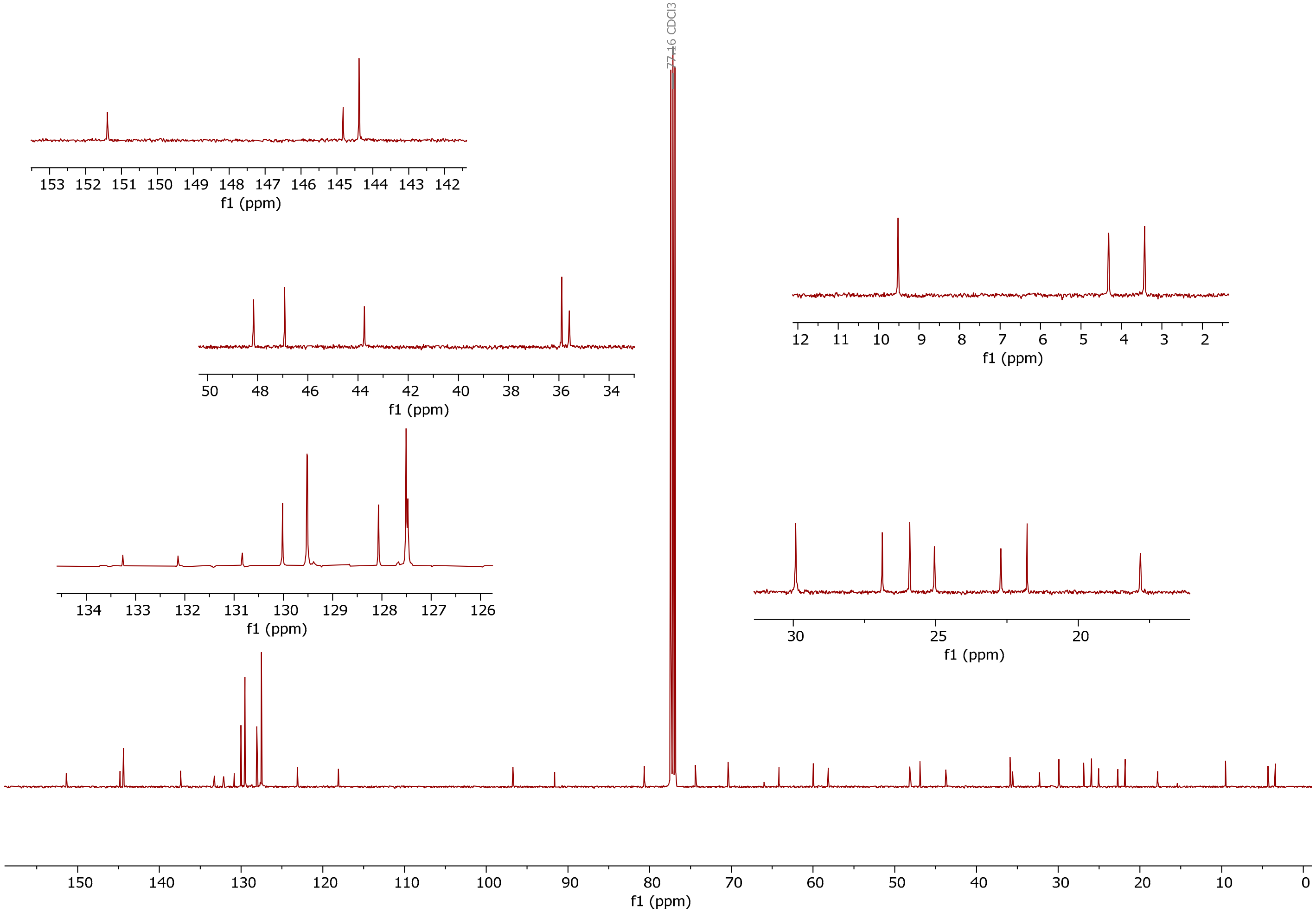

Figure S17 ^{1}H NMR spectrum of 6-*O*-(5-tosyloxypentyl)-6-*O*-desmethyl-3-*O*-trityl-diprenorphine (**26d**, TPe-TDDPN) in $CDCl_3$

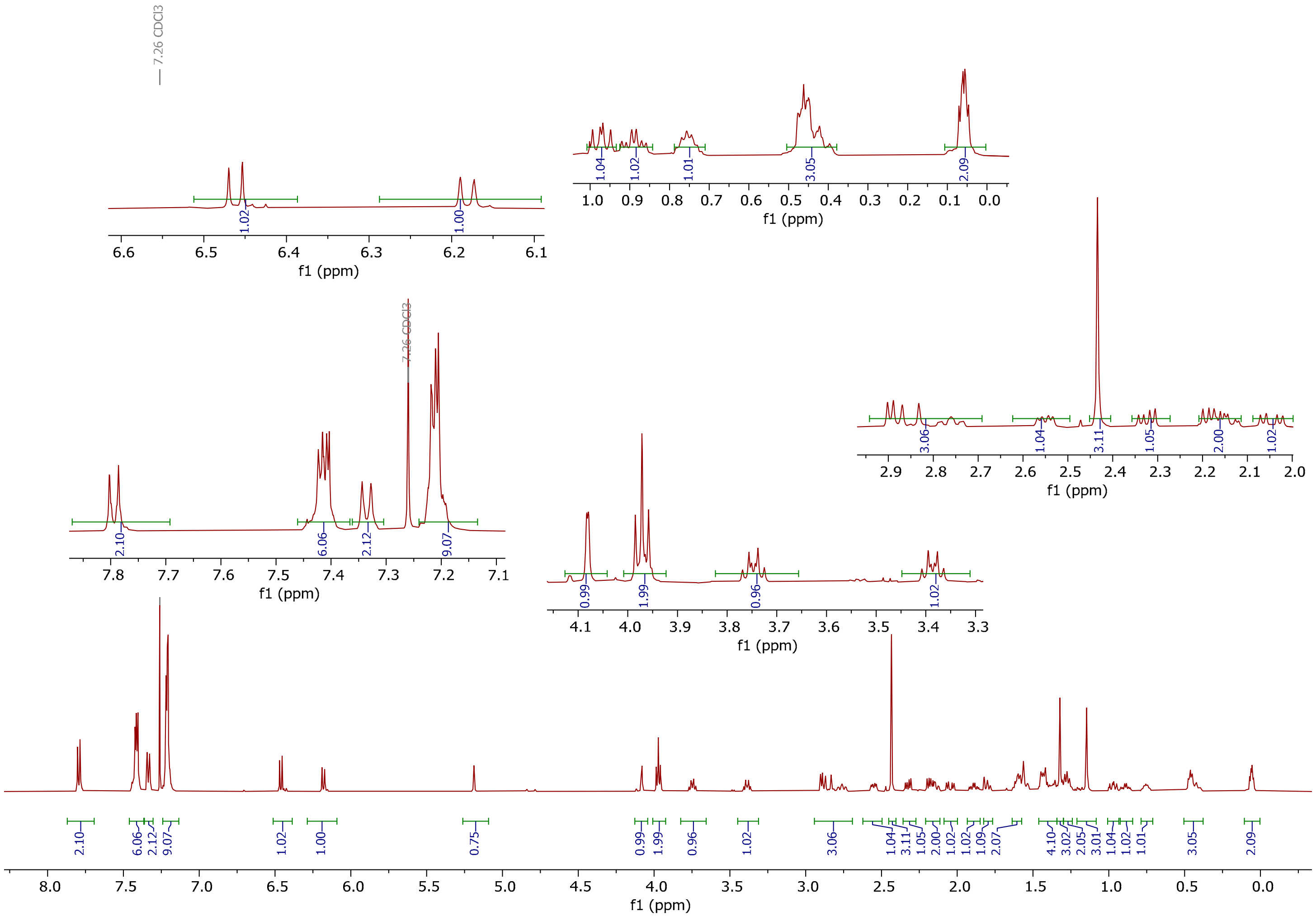

Figure S18 ^{1}H NMR spectrum of 6-*O*-(5-tosyloxypentyl)-6-*O*-desmethyl-3-*O*-trityl-diprenorphine (**26d**, TPe-TDDPN) in $CDCl_3$

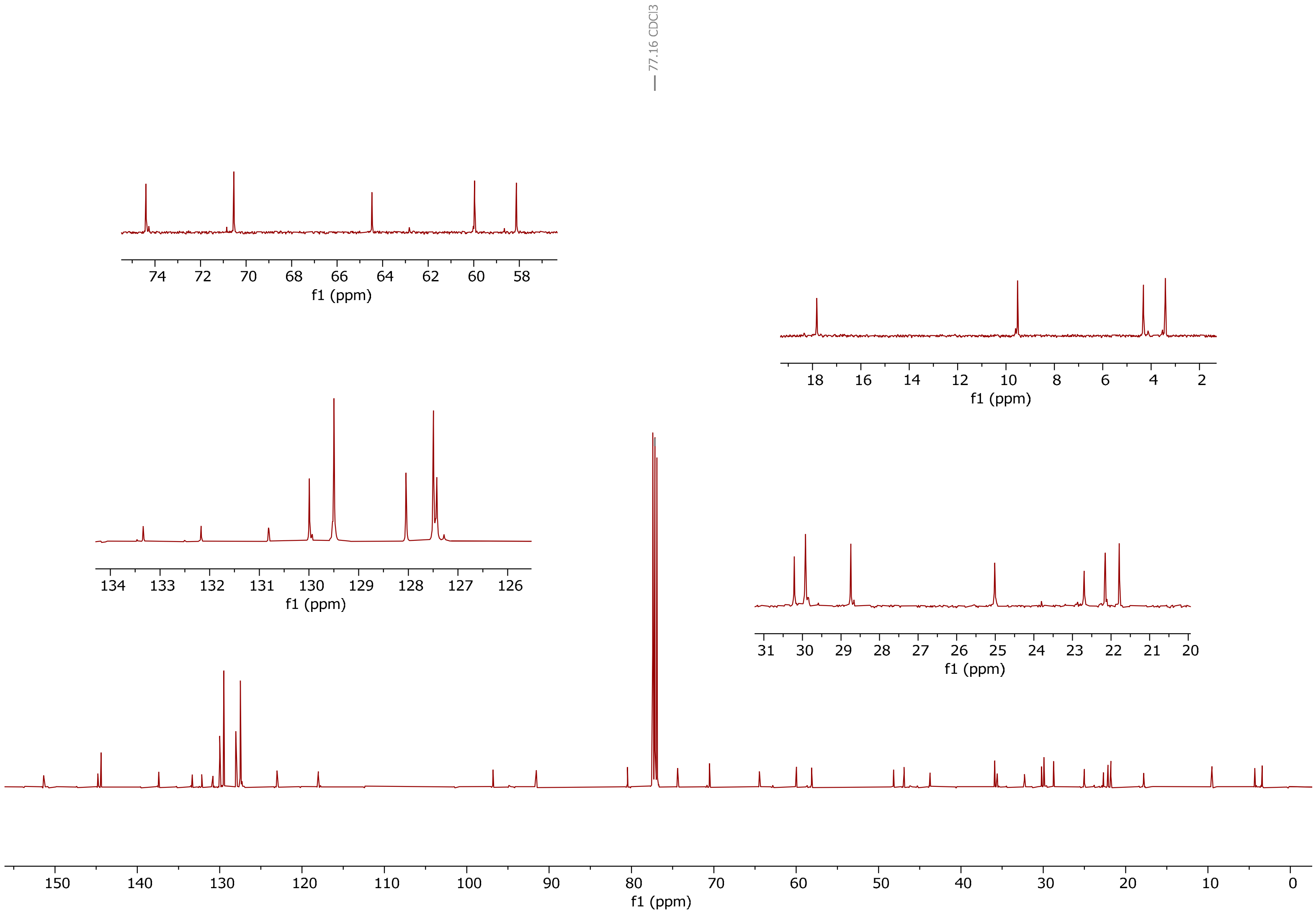

Figure S19 ^{1}H NMR spectrum of 6-*O*-(3-tosyloxypropyl)-6-*O*-desmethyl-3-*O*-trityl-diprenorphine (**27b**, FP-TDDPN) in $CDCl_3$

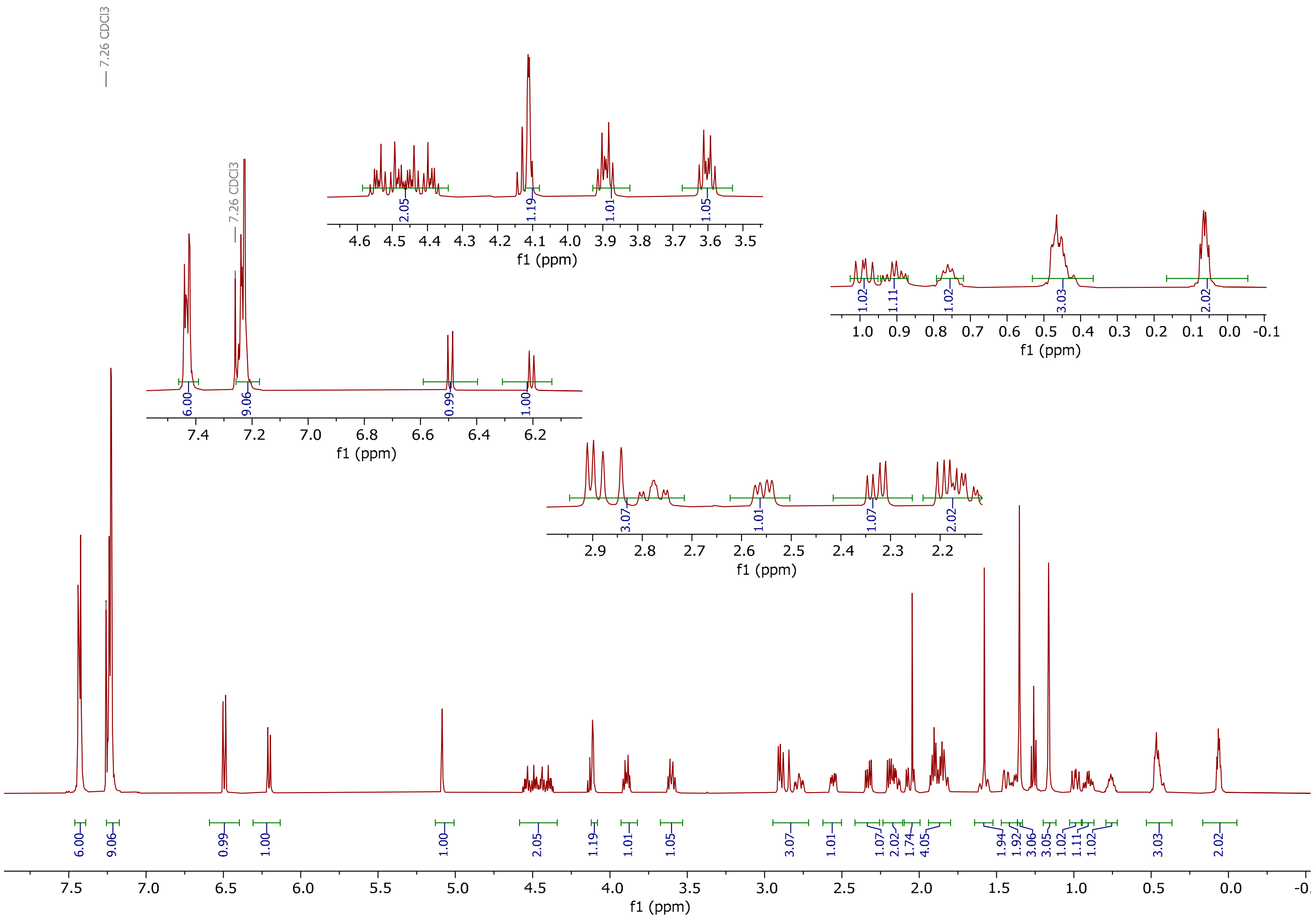

Figure S20 ^{13}C NMR spectrum of 6-*O*-(3-tosyloxypropyl)-6-*O*-desmethyl-3-*O*-trityl-diprenorphine (**27b**, FP-TDDPN) in $CDCl_3$

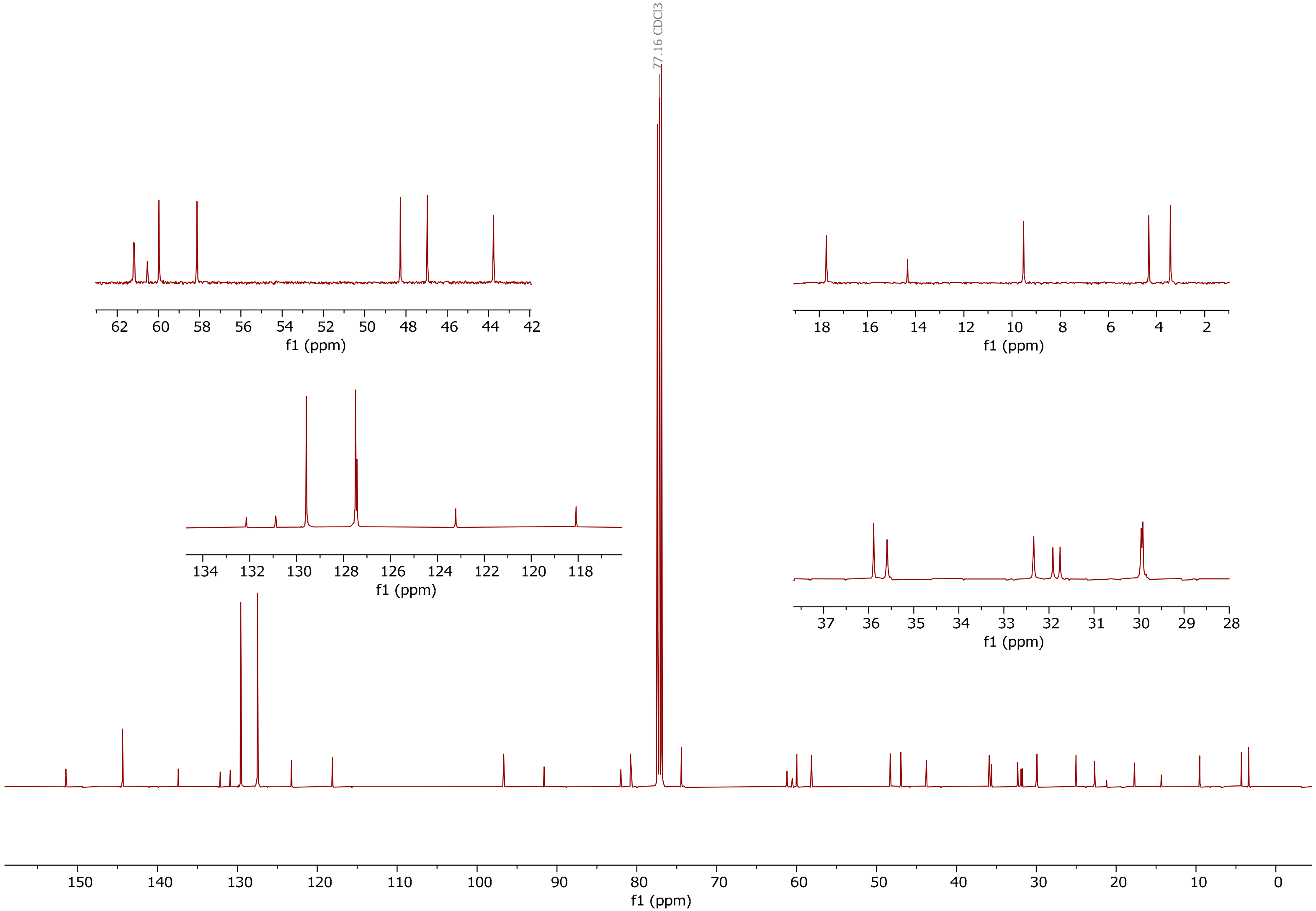

Figure S21 ^{19}F NMR spectrum of 6-*O*-(3-tosyloxypropyl)-6-*O*-desmethyl-3-*O*-trityl-diprenorphine (**27b**, FP-TDDPN) in $CDCl_3$

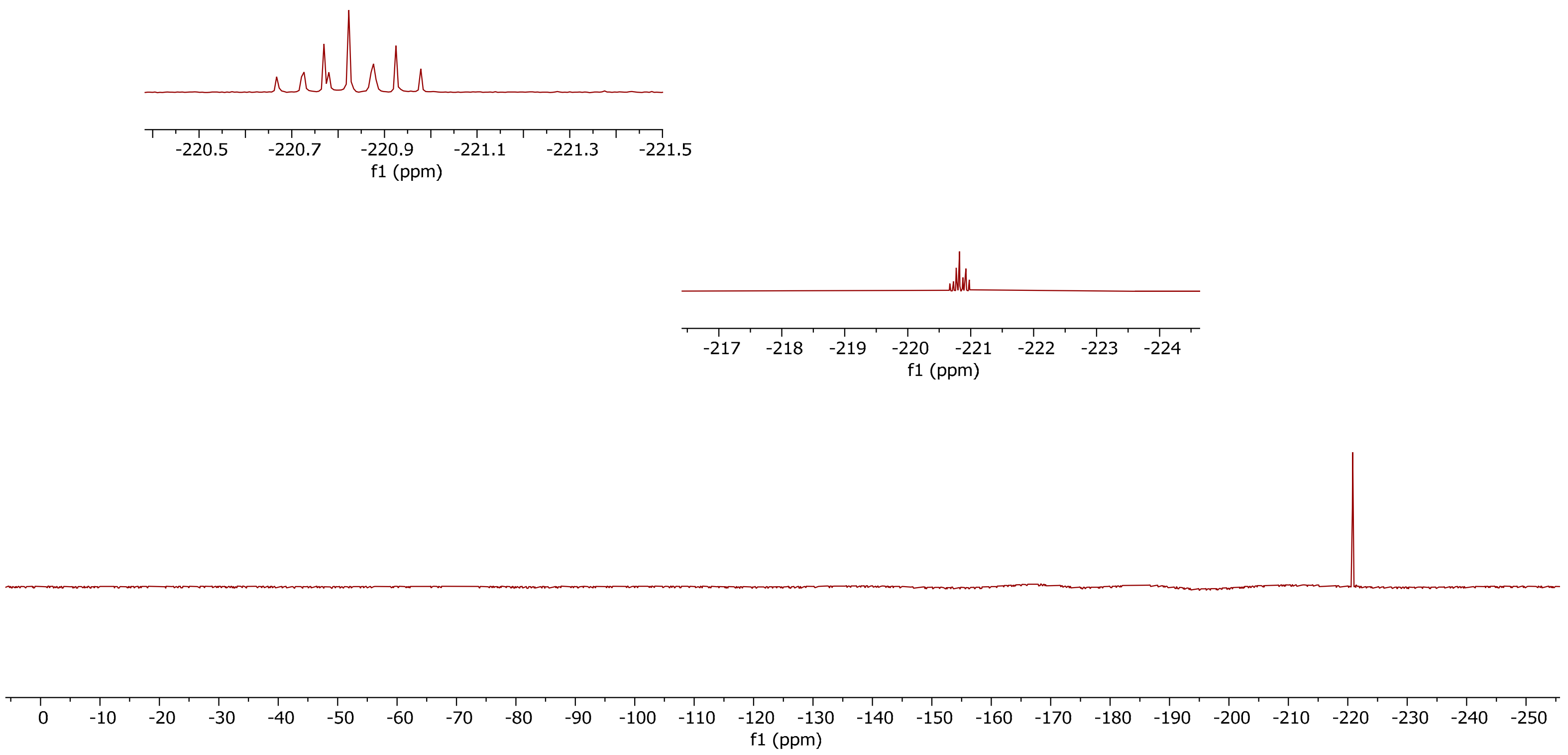

Figure S22 ^{1}H NMR spectrum of 6-*O*-(4-fluorobutyl)-6-*O*-desmethyl-3-*O*-trityl-diprenorphine (**27c**, FB-TDDPN) in $CDCl_3$

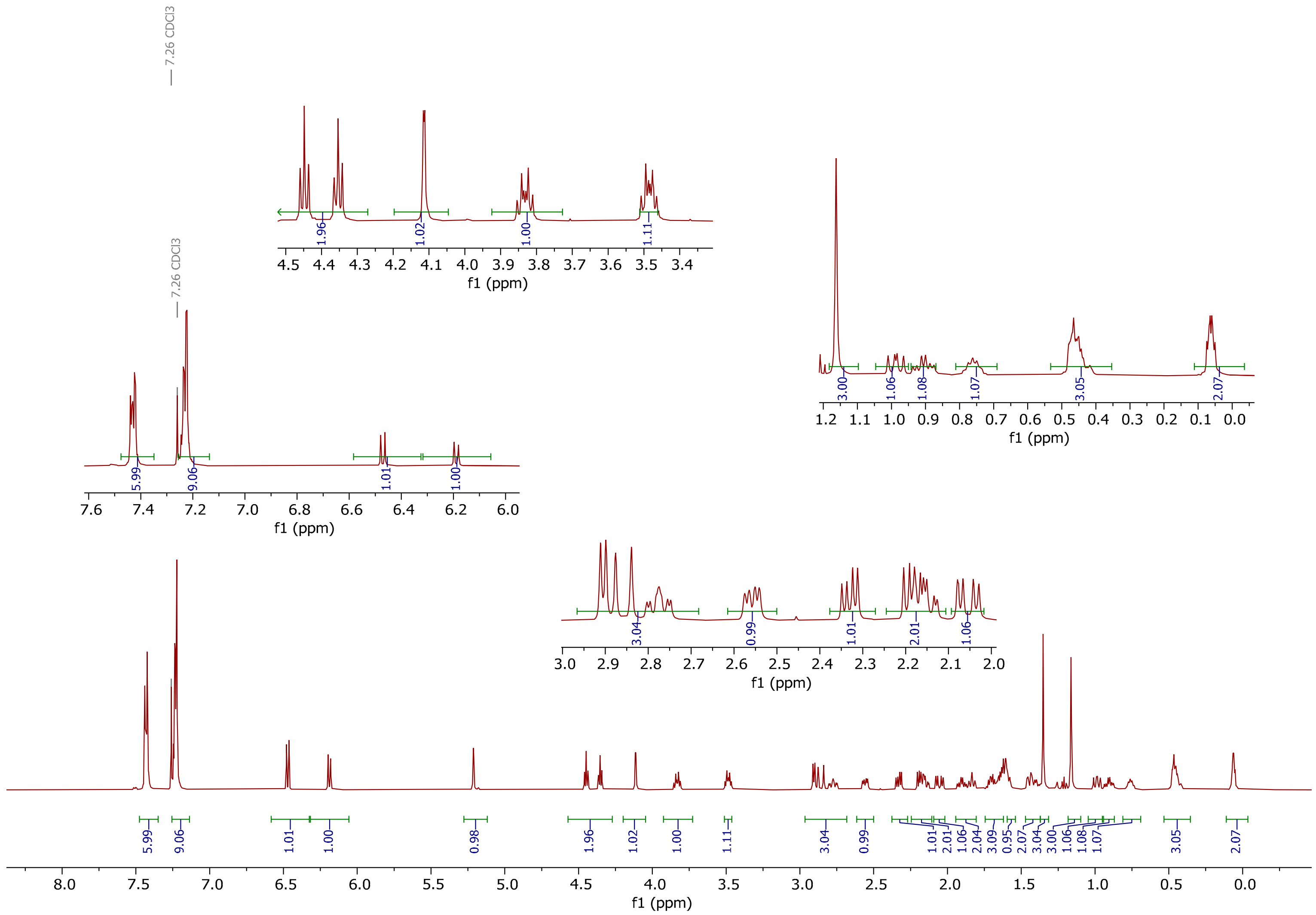

Figure S23 ^{13}C NMR spectrum of 6-*O*-(4-fluorobutyl)-6-*O*-desmethyl-3-*O*-trityl-diprenorphine (**27c**, FB-TDDPN) in $CDCl_3$

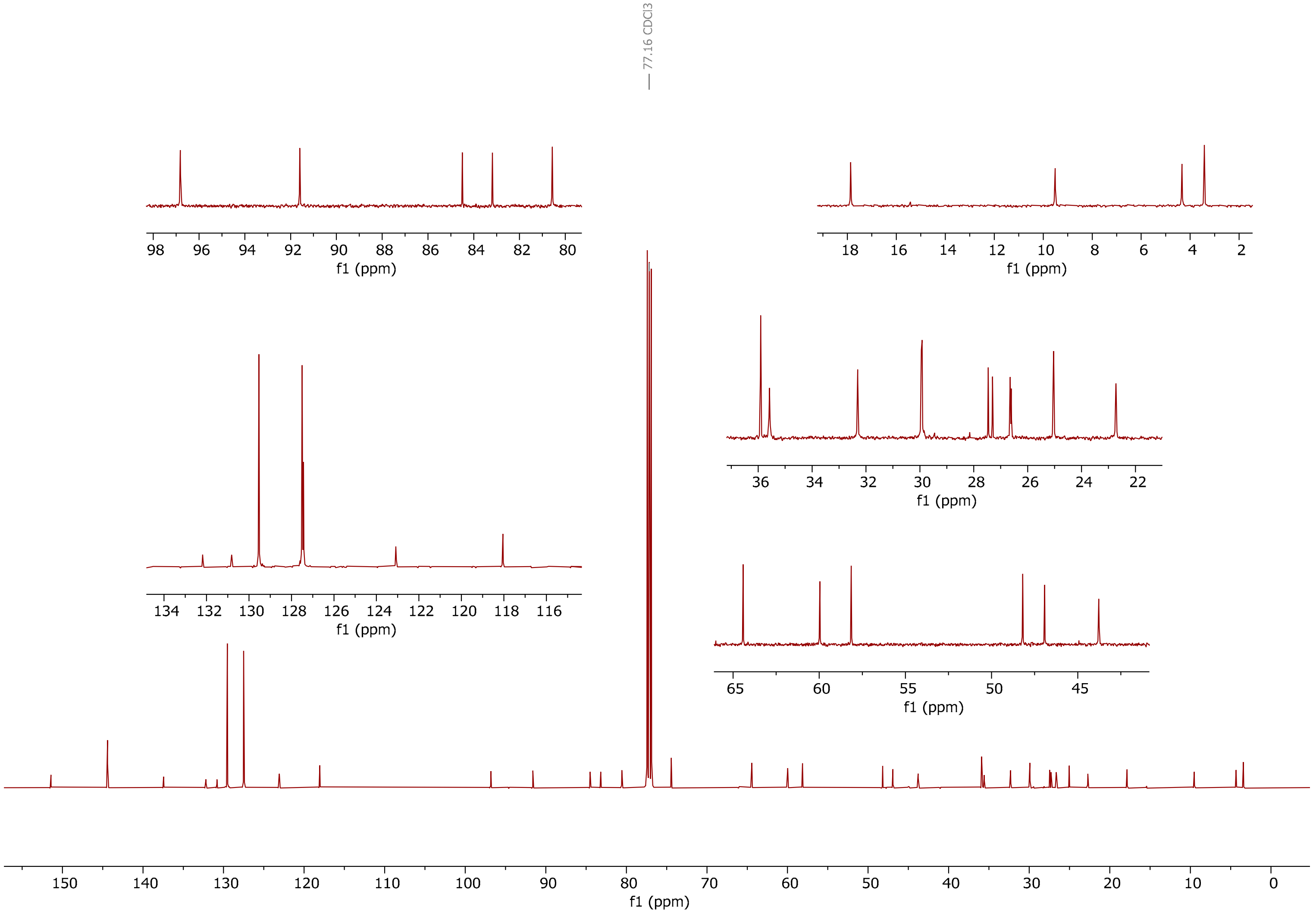

Figure S24 ^{19}F NMR spectrum of 6-*O*-(4-fluorobutyl)-6-*O*-desmethyl-3-*O*-trityl-diprenorphine (**27c**, FB-TDDPN) in $CDCl_3$

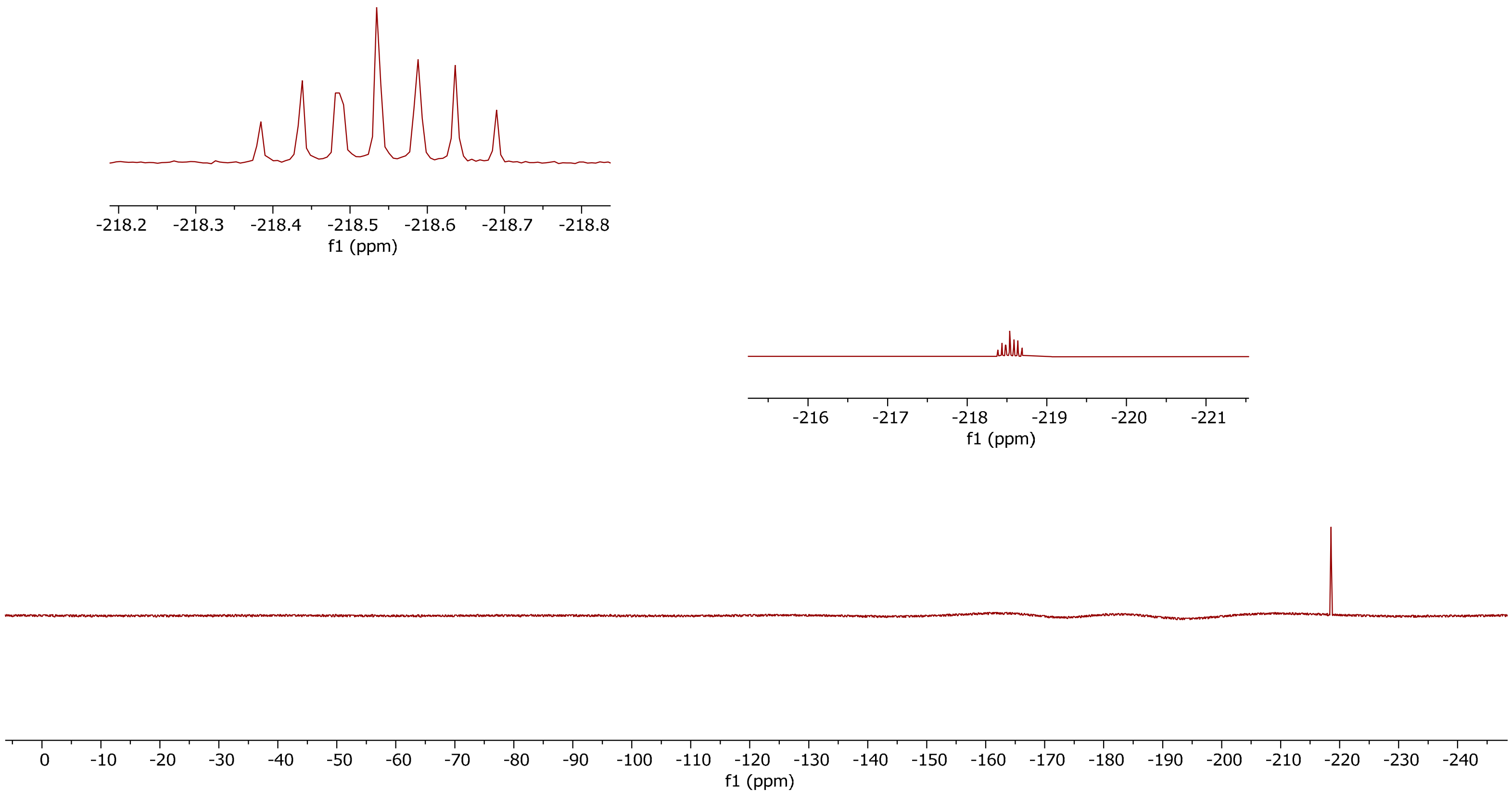

Figure S25 ^{1}H NMR spectrum of 6-*O*-(4-fluoropentyl)-6-*O*-desmethyl-3-*O*-trityl-diprenorphine (**27d**, FPe-TDDPN) in $CDCl_3$

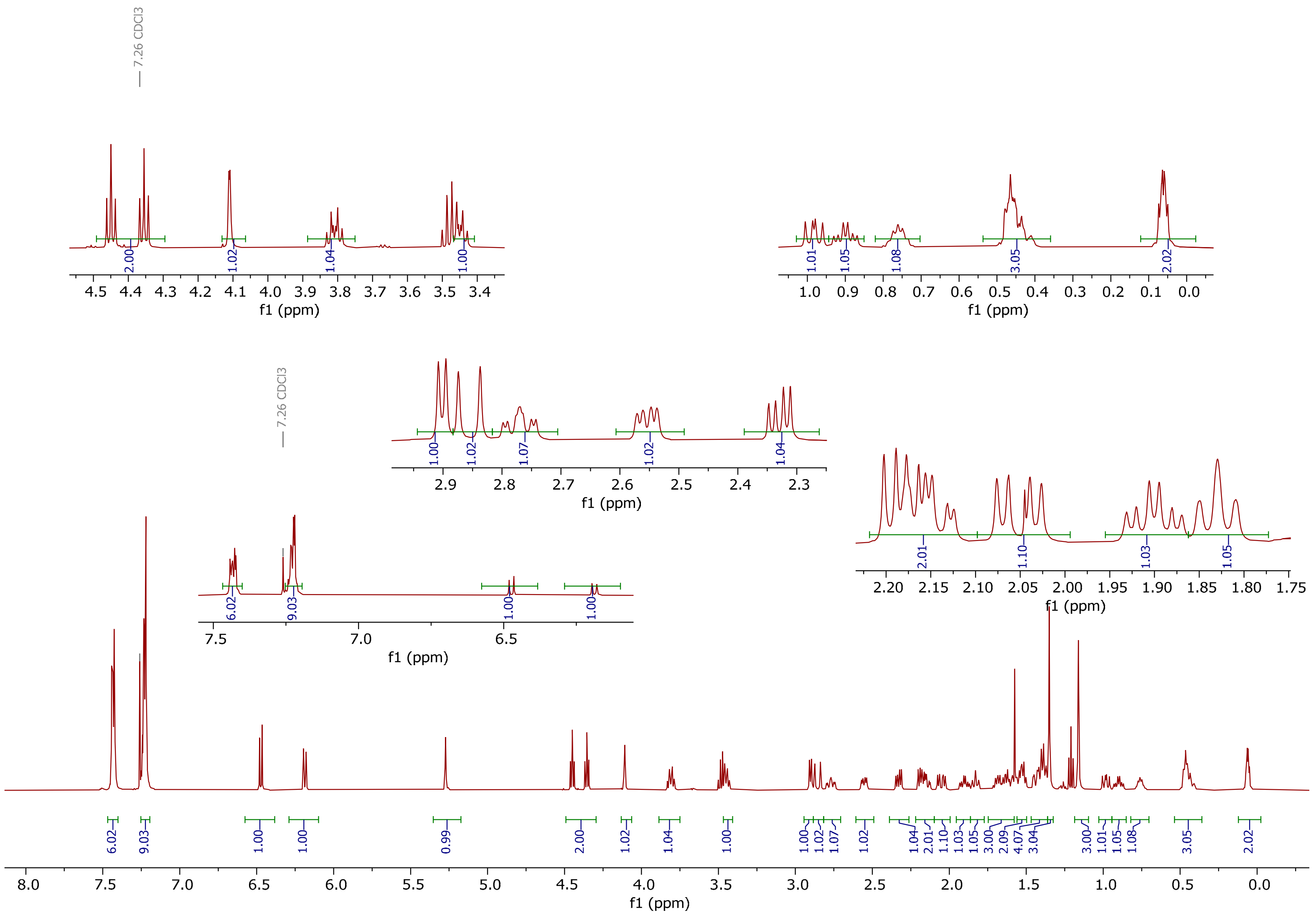

Figure S26 ^{13}C NMR spectrum of 6-*O*-(4-fluoropentyl)-6-*O*-desmethyl-3-*O*-trityl-diprenorphine (**27d**, FPe-TDDPN) in $CDCl_3$

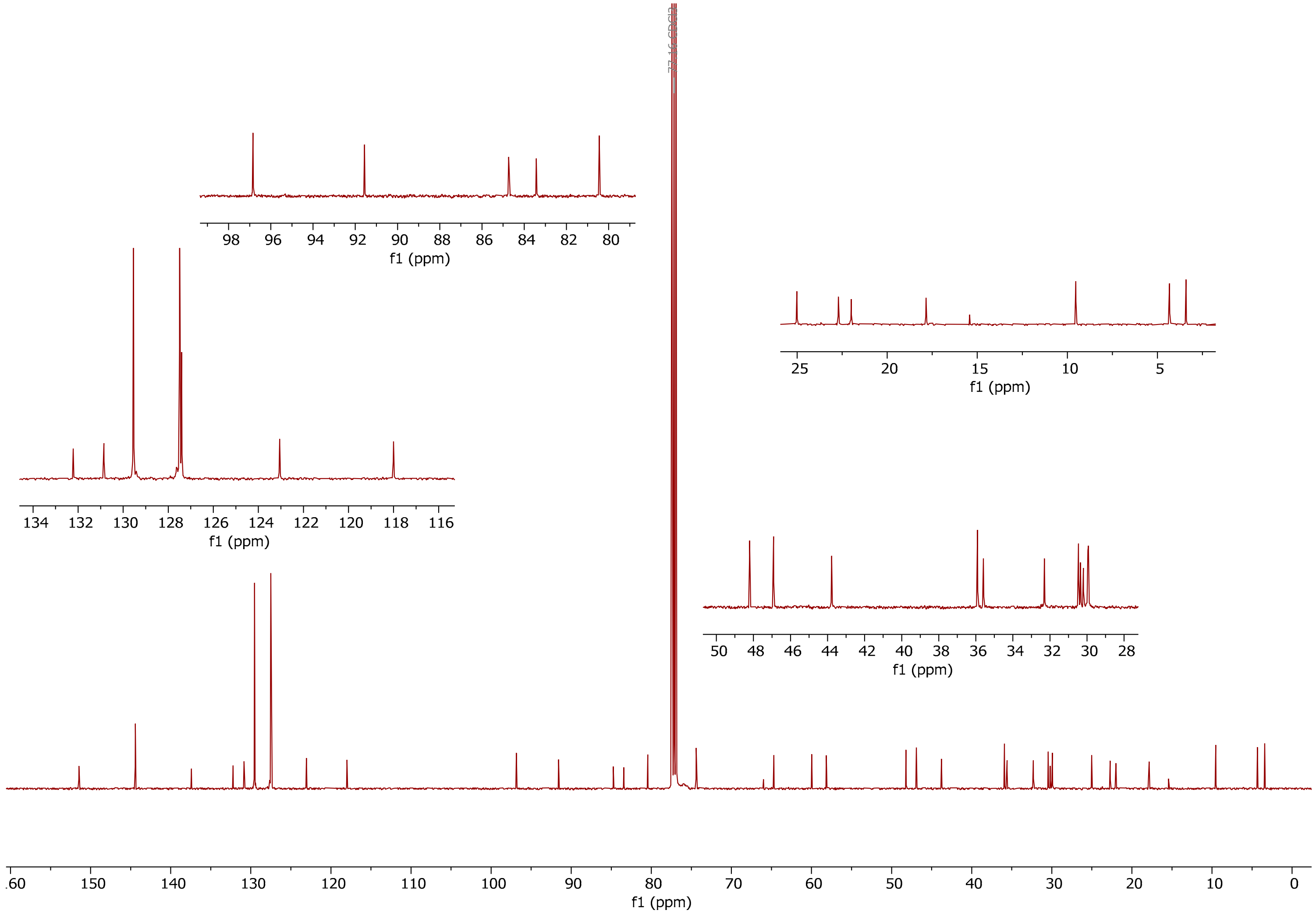

Figure S27 ^{19}F NMR spectrum of 6-*O*-(5-fluoropentyl)-6-*O*-desmethyl-3-*O*-trityl-diprenorphine (**27d**, FPe-TDDPN) in $CDCl_3$

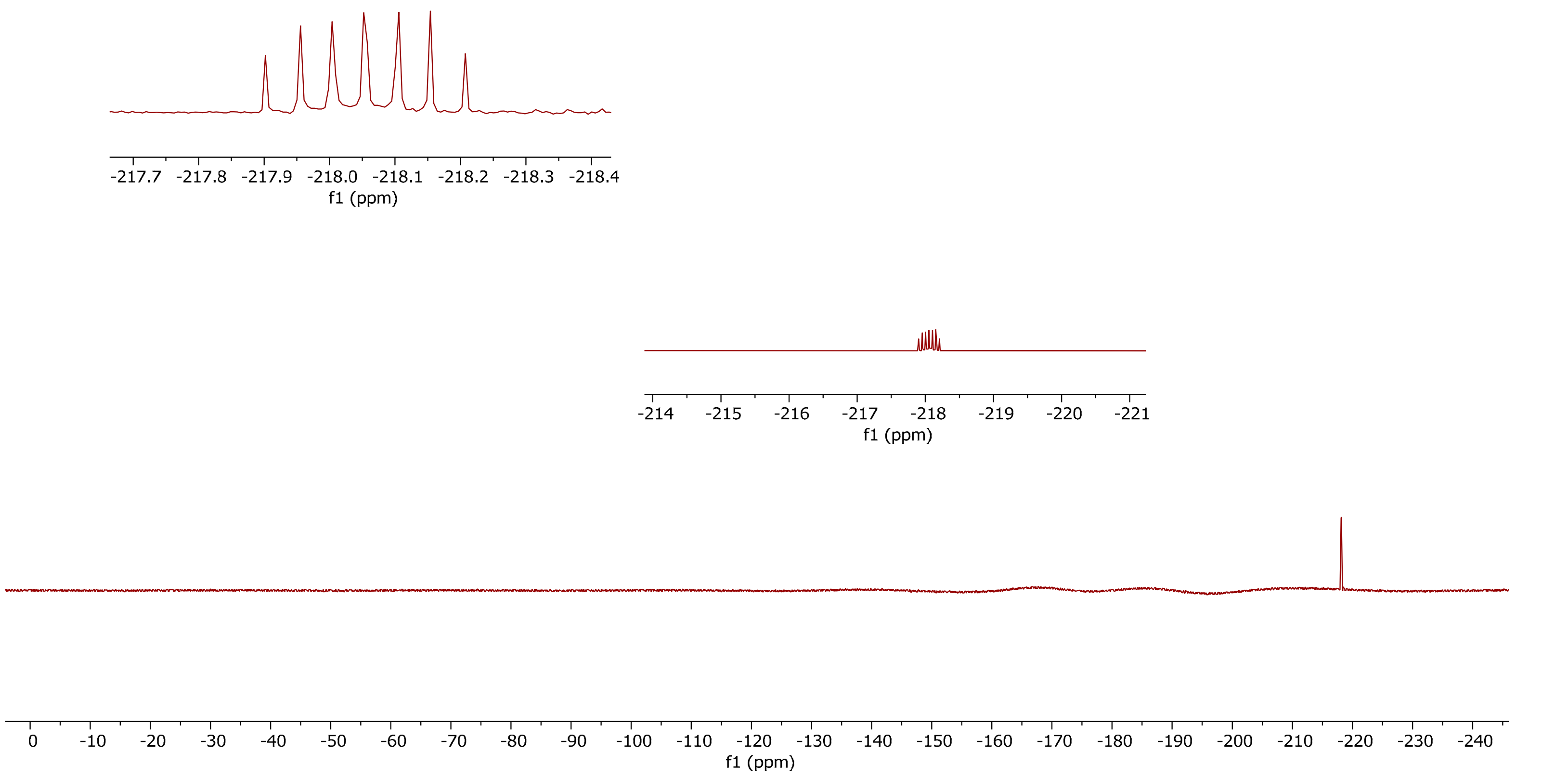

Figure S28 ^{1}H NMR spectrum of 6-*O*-(3-fluoropropyl)-6-*O*-desmethyl-diprenorphine (**28b**, FP-DPN) in $CDCl_3$

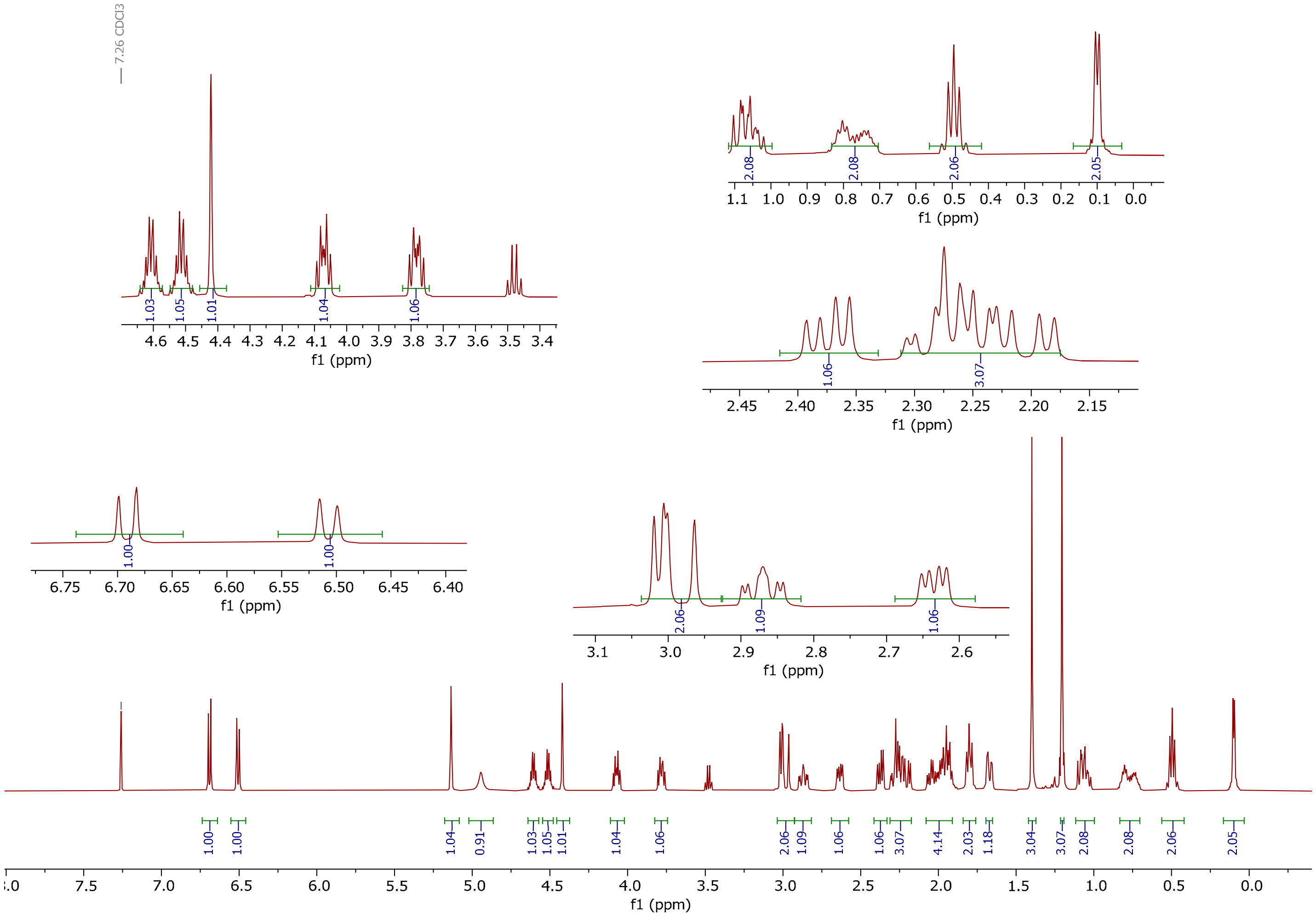

Figure S29 ^{13}C NMR spectrum of 6-*O*-(3-fluoropropyl)-6-*O*-desmethyl-diprenorphine (**28b**, FP-DPN) in $CDCl_3$

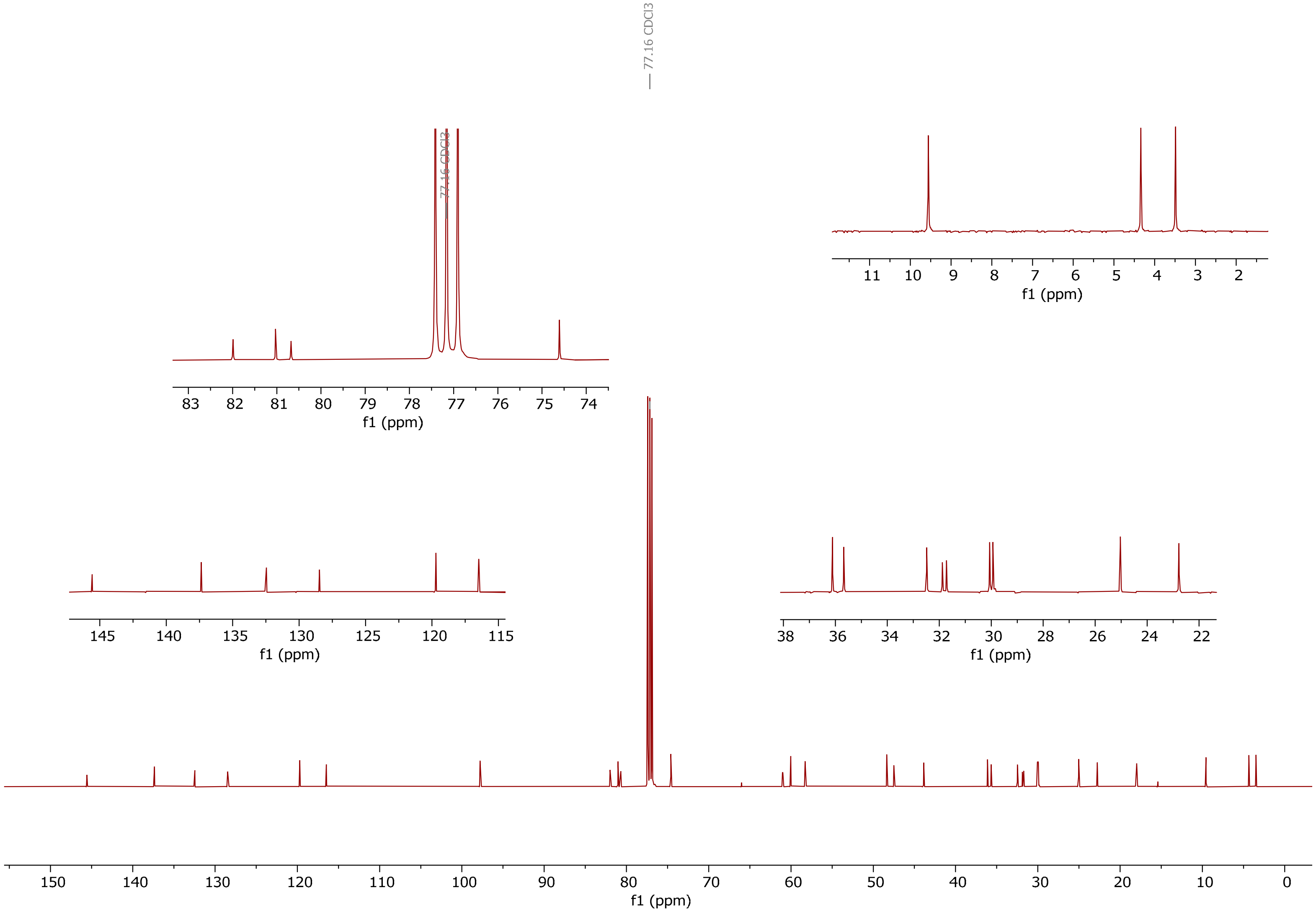

Figure S30 ^{19}F NMR spectrum of 6-*O*-(3-fluoropropyl)-6-*O*-desmethyl-diprenorphine (**28b**, FP-DPN) in $CDCl_3$

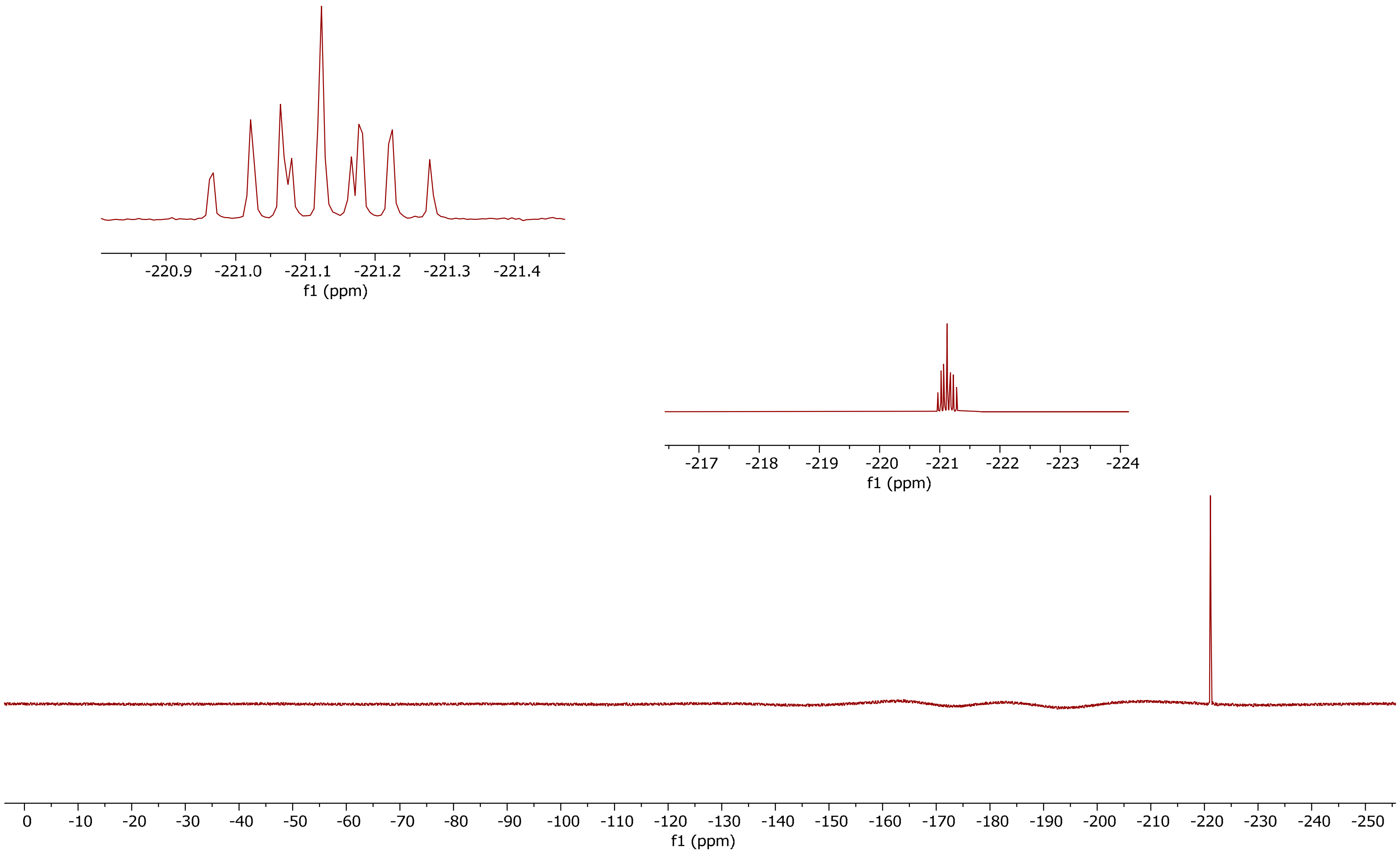

Figure S31 ^{1}H NMR spectrum of 6-*O*-(4-fluorobutyl)-6-*O*-desmethyl-diprenorphine (**28c**, FB-DPN) in $CDCl_3$

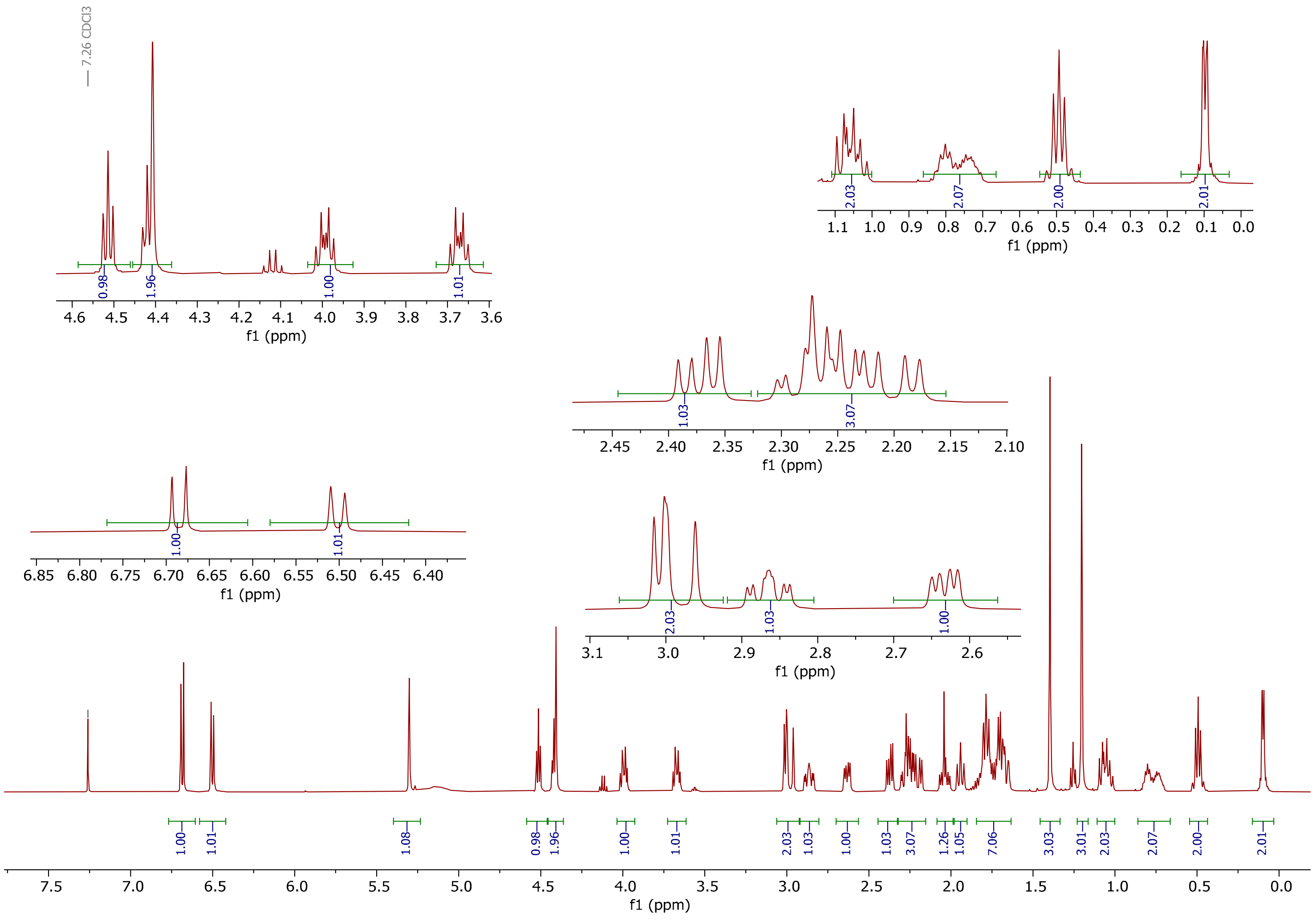

Figure S32 ^{13}C NMR spectrum of 6-*O*-(4-fluorobutyl)-6-*O*-desmethyl-diprenorphine (**28c**, FB-DPN) in $CDCl_3$

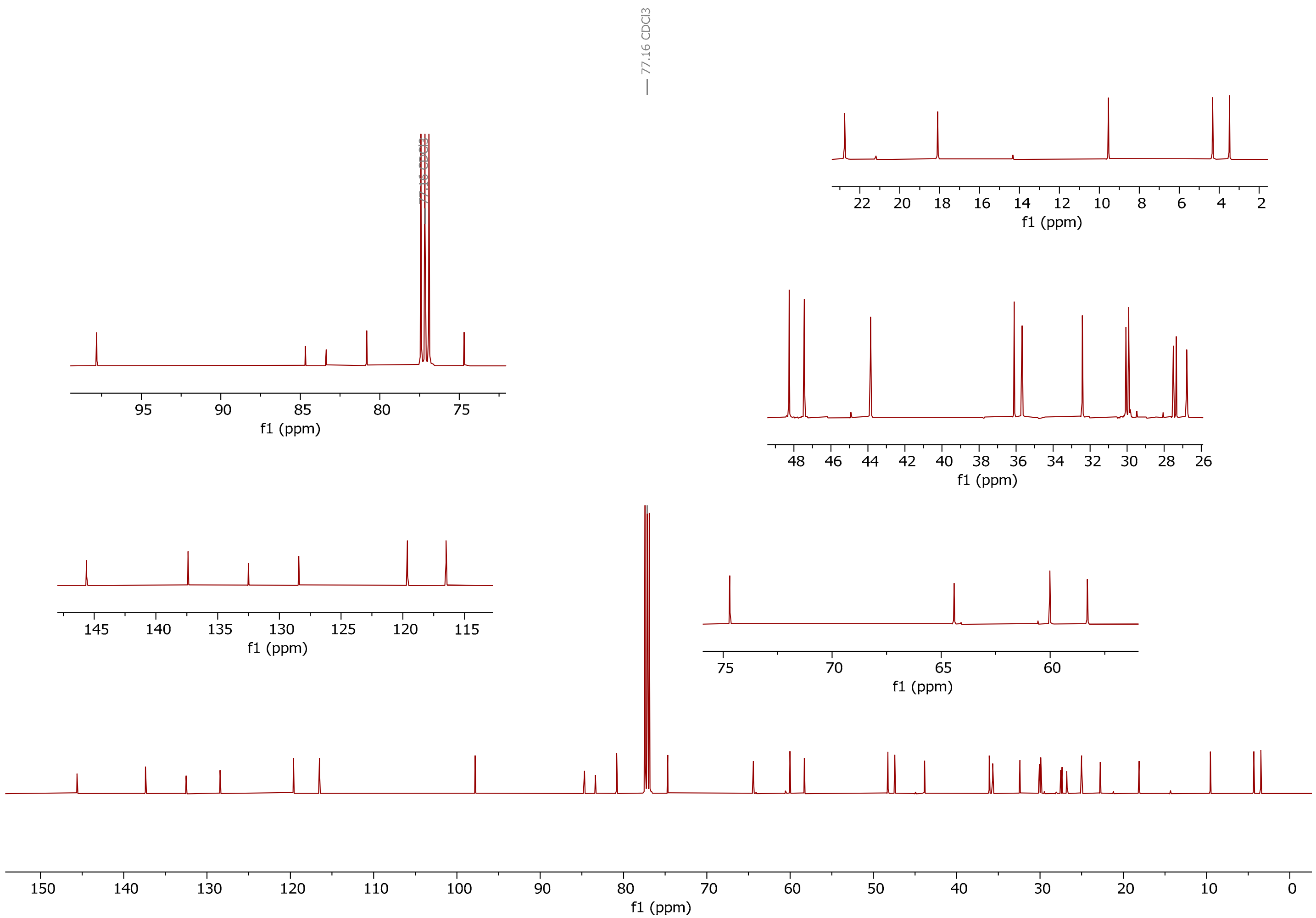

Figure S33 ^{19}F NMR spectrum of 6-*O*-(4-fluorobutyl)-6-*O*-desmethyl-diprenorphine (**28c**, FB-DPN) in $CDCl_3$

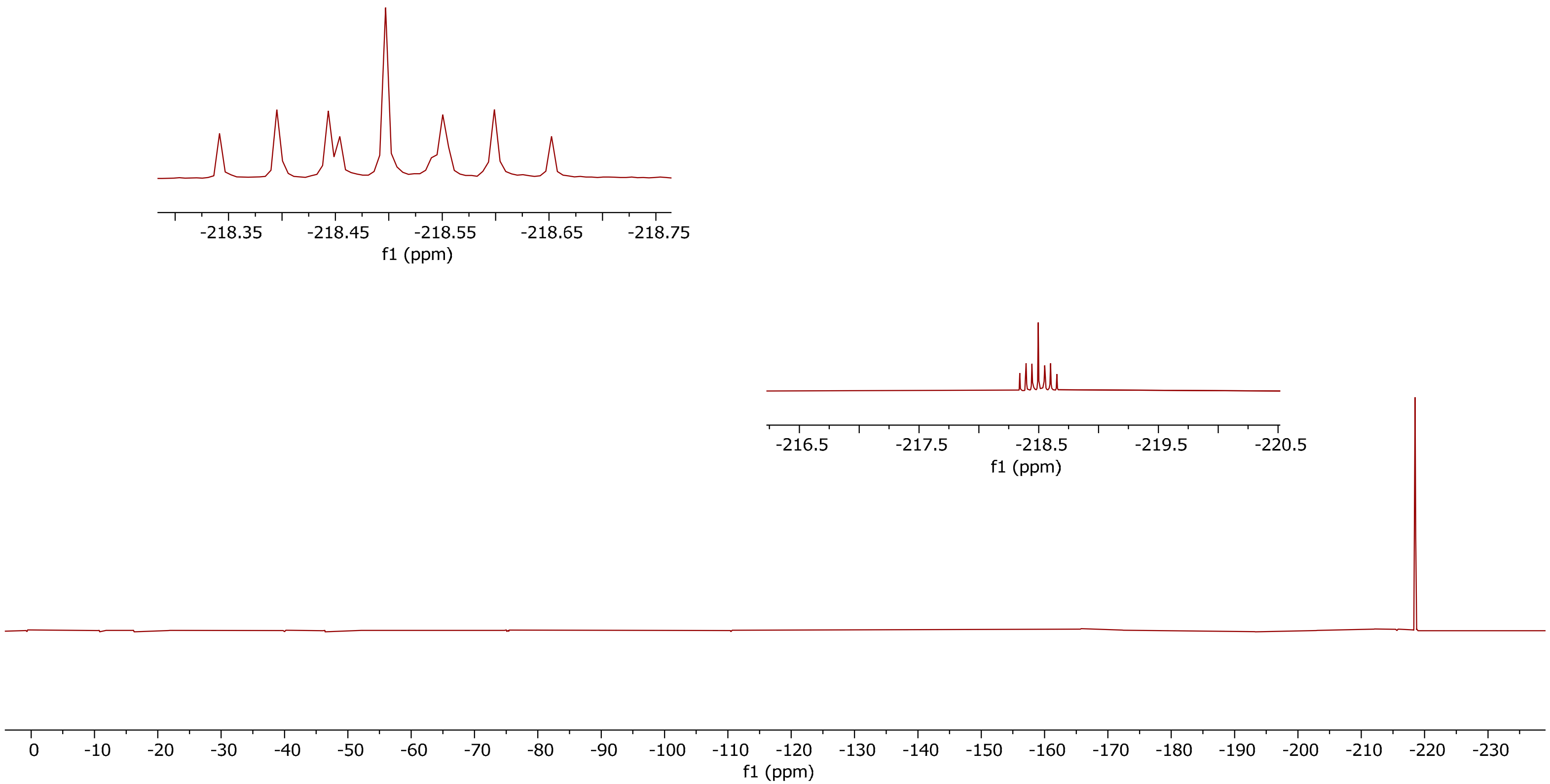

Figure S34 ^{1}H NMR spectrum of 6-*O*-(5-fluoropentyl)-6-*O*-desmethyl-diprenorphine (**28d**, FPe-DPN) in $CDCl_3$

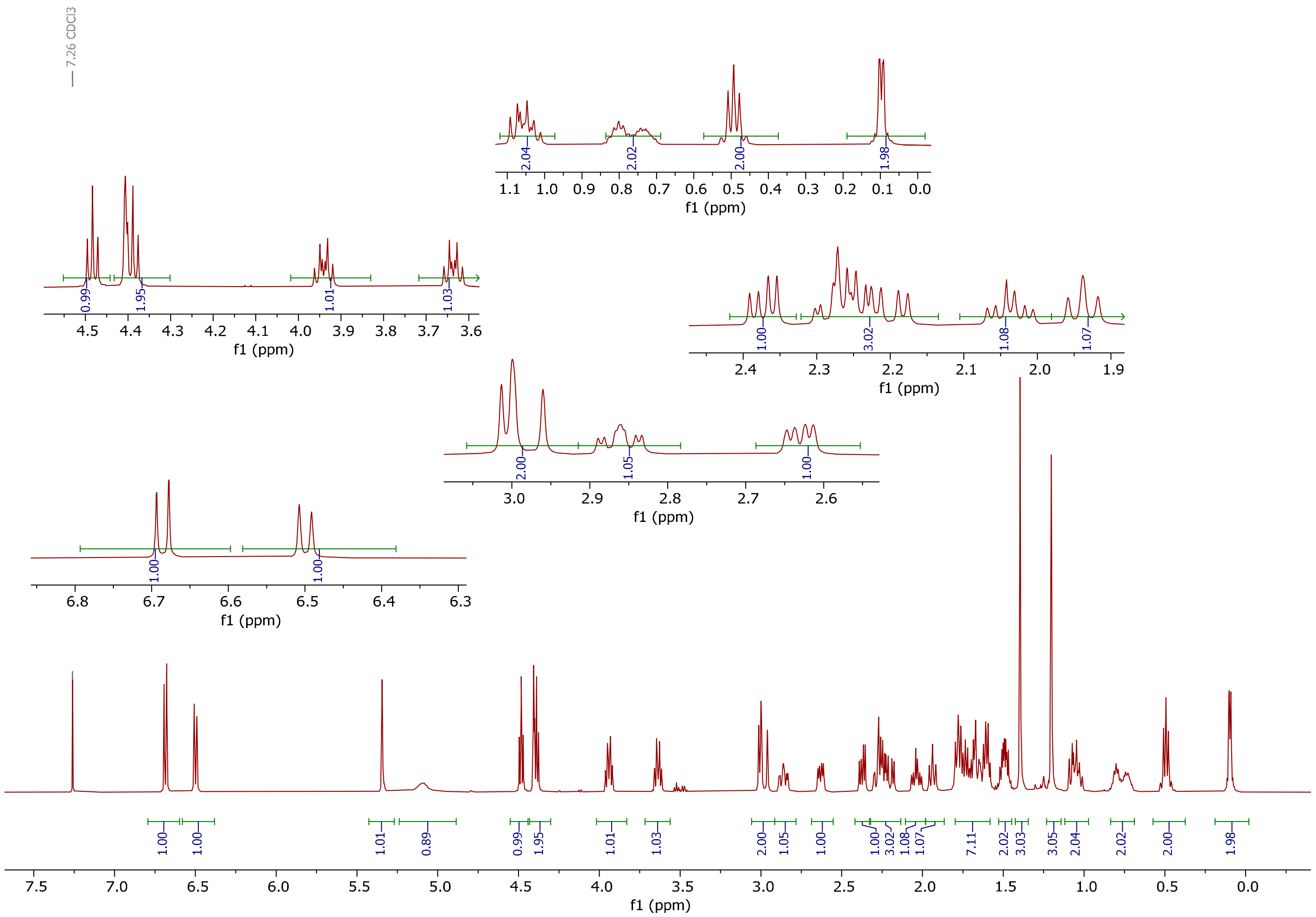

Figure S35 ^{13}C NMR spectrum of 6-*O*-(5-fluoropentyl)-6-*O*-desmethyl-diprenorphine (**28d**, FPe-DPN) in $CDCl_3$

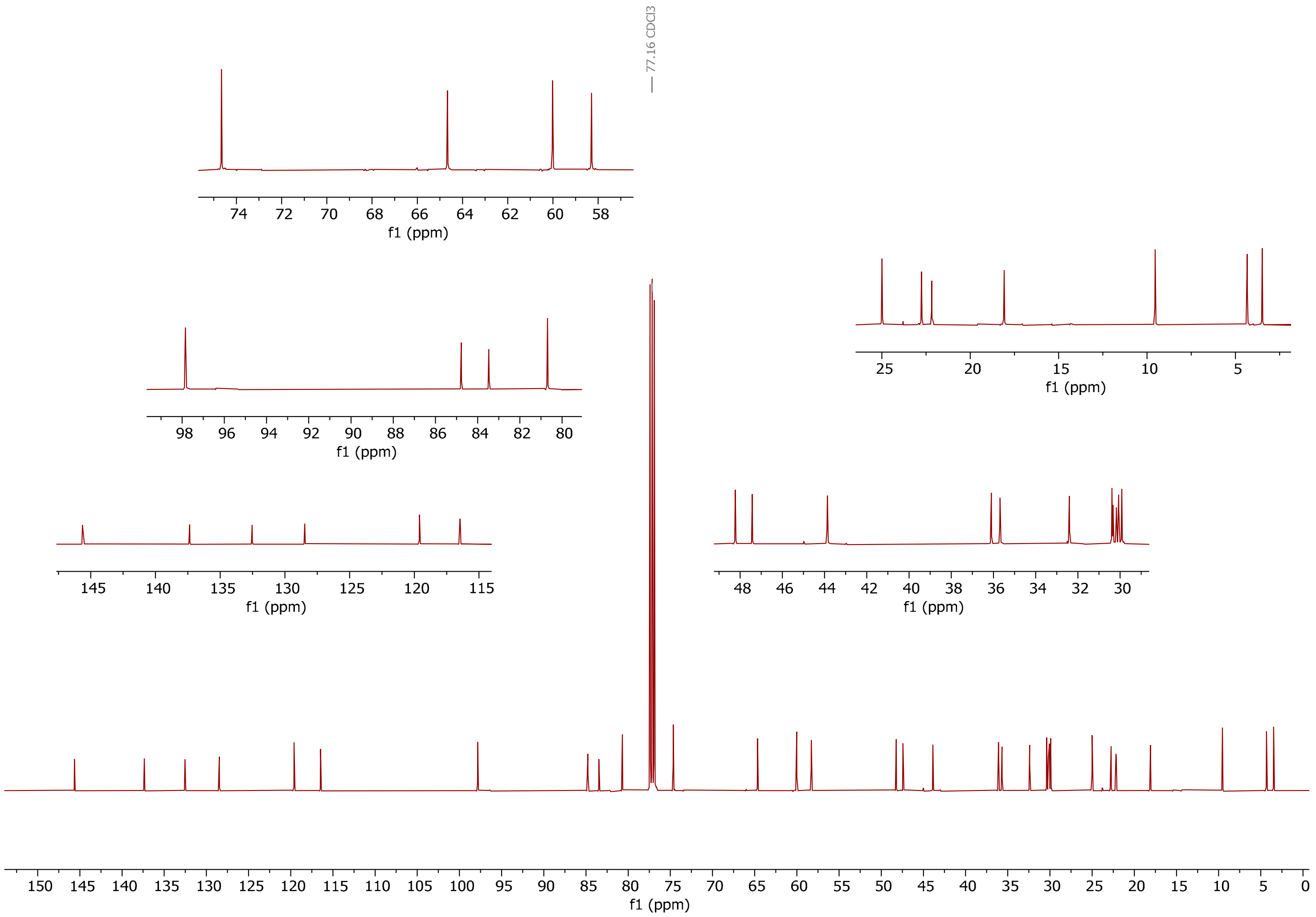

Figure S36 ^{19}F NMR spectrum of 6-*O*-(5-fluoropentyl)-6-*O*-desmethyl-diprenorphine (**28d**, FPe-DPN) in $CDCl_3$

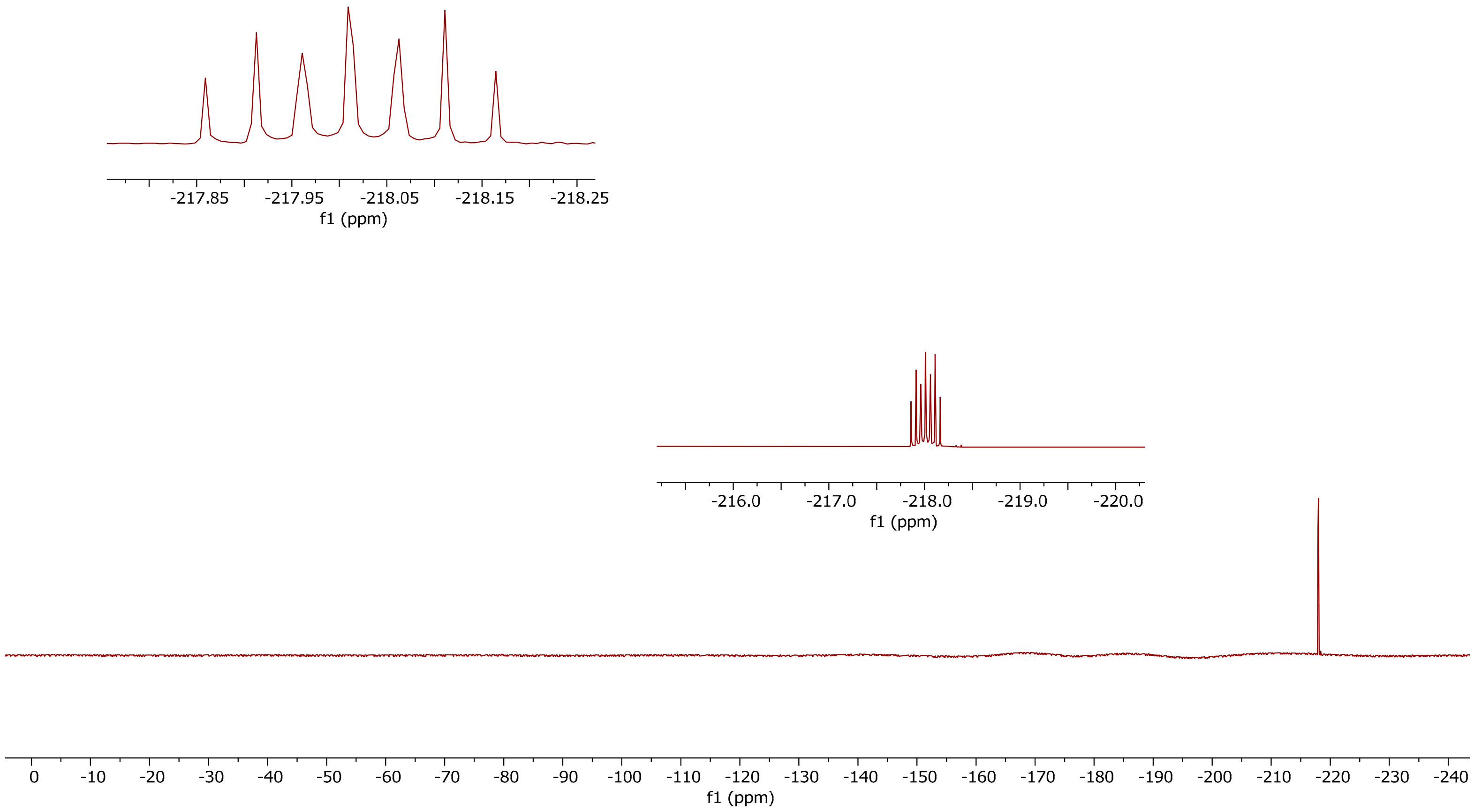

Figure S37 ^{1}H NMR spectrum of 6-*O*-(3-hydroxypropyl)-6-*O*-desmethyl-diprenorphine (**29b**, HP-DPN) in $CDCl_3$

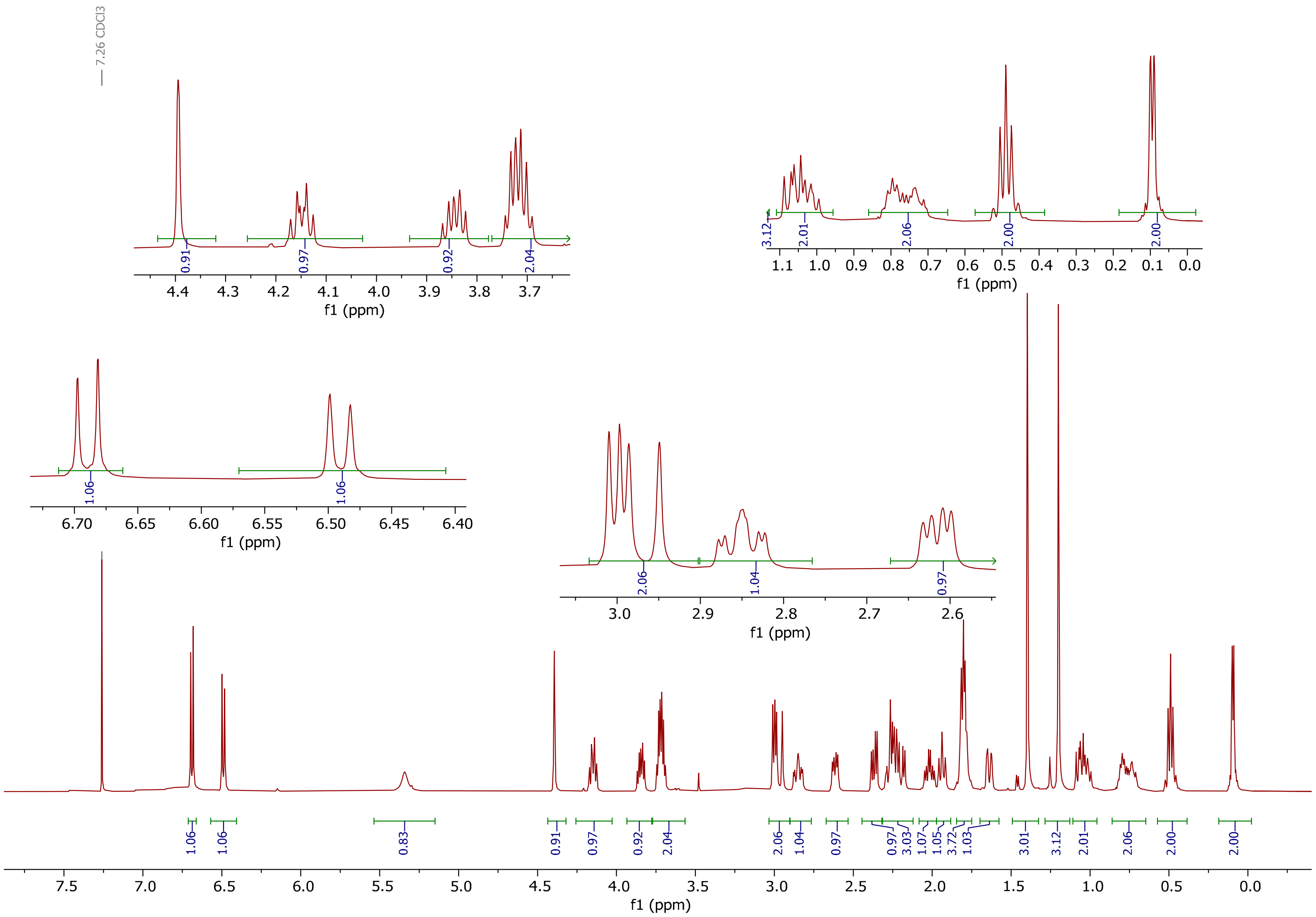

Figure S38 ^{13}C NMR spectrum of 6-*O*-(3-hydroxypropyl)-6-*O*-desmethyl-diprenorphine (**29b**, HP-DPN) in $CDCl_3$

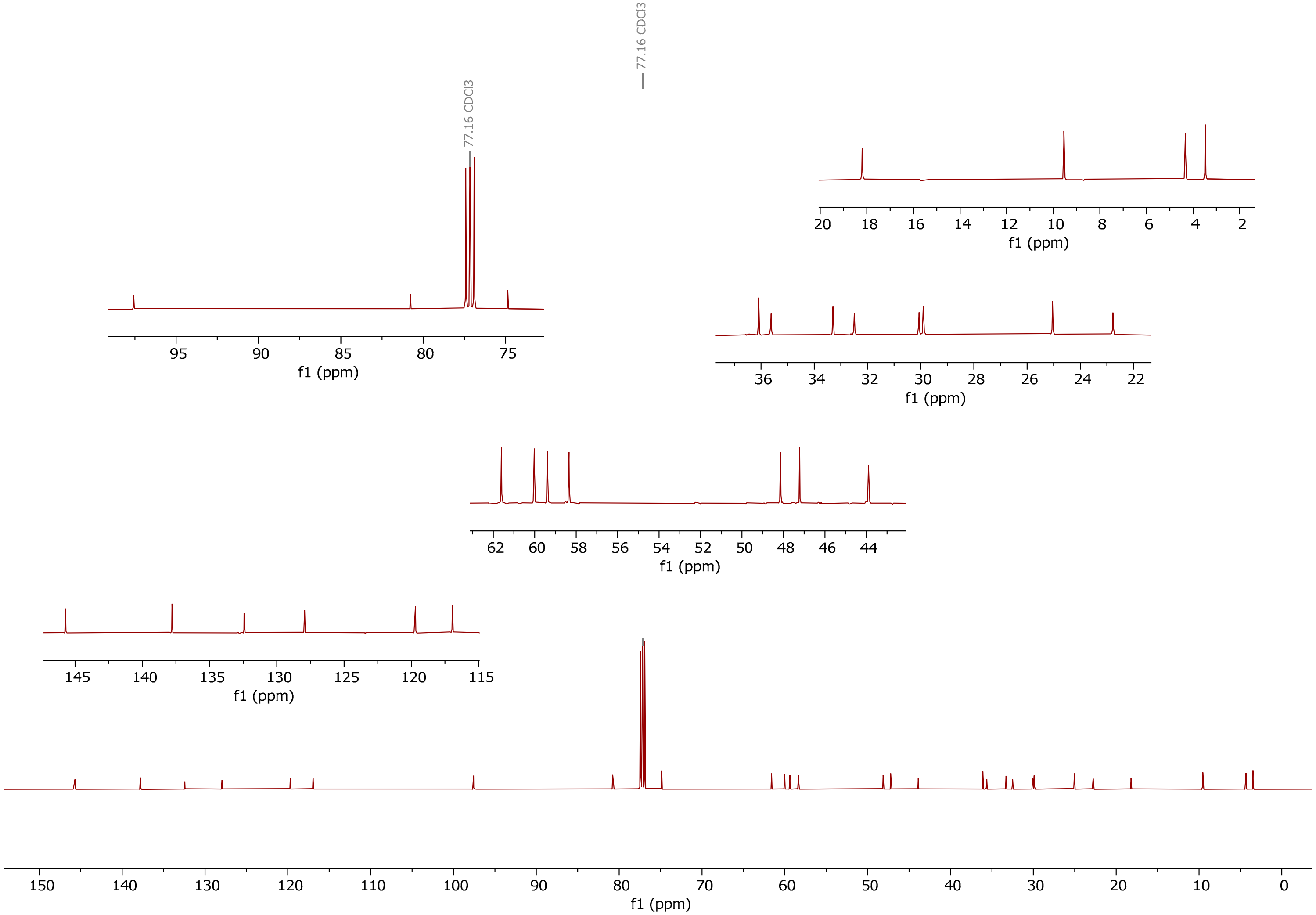
77.16 CDCl3
77.16 CDCl3
95
90
85
80
75
f1 (ppm)
20
18
16
14
12
10
8
6
4
2
f1 (ppm)
36
34
32
30
28
26
24
22
f1 (ppm)
62
60
58
56
54
52
50
48
46
44
f1 (ppm)
145
140
135
130
125
120
115
f1 (ppm)
150
140
130
120
110
100
90
80
70
60
50
40
30
20
10
0
f1 (ppm)

Figure S39 ^{1}H NMR spectrum of 6-*O*-(4-hydroxybutyl)-6-*O*-desmethyl-diprenorphine (**29c**, HB-DPN) in $CDCl_3$

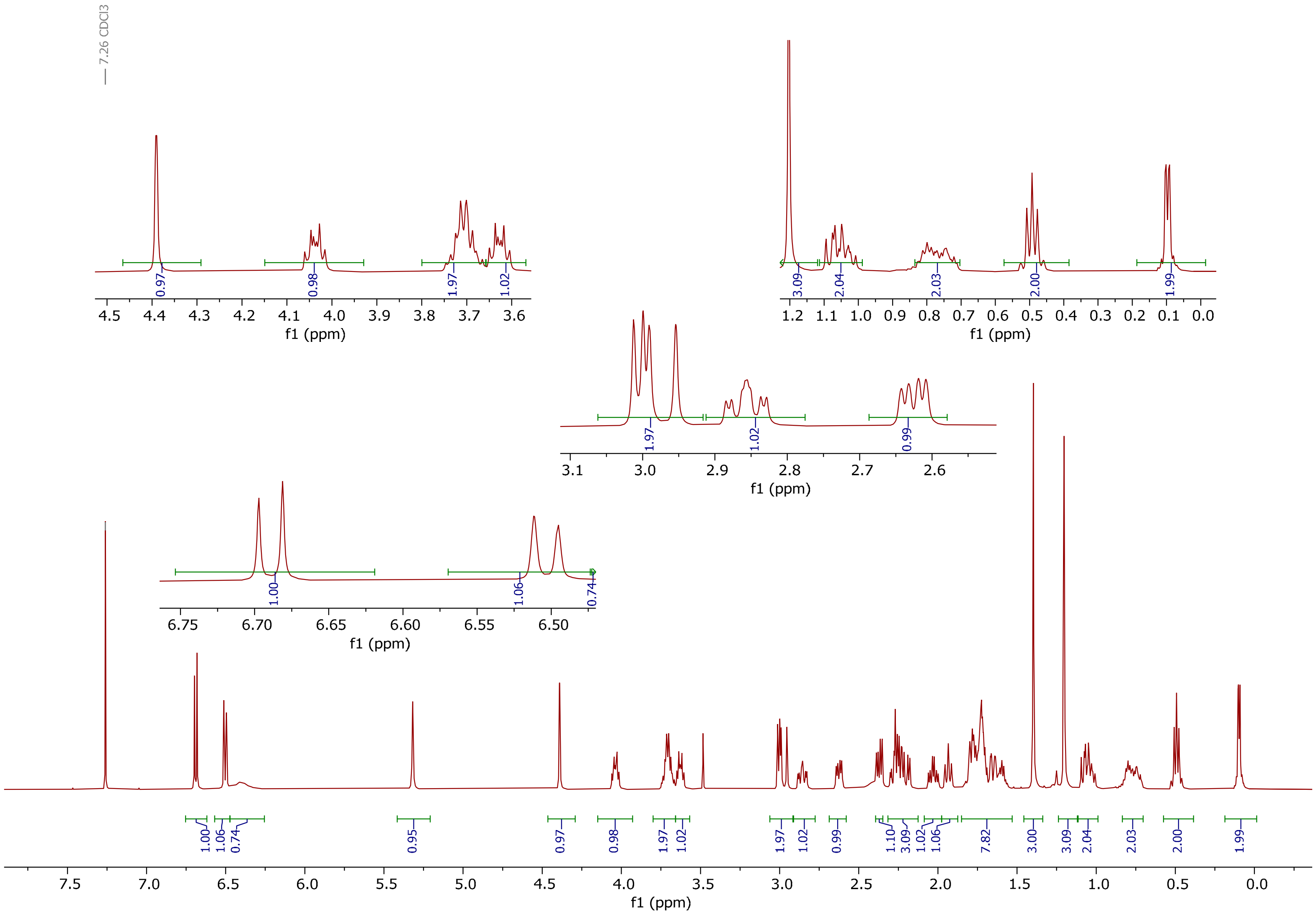

Figure S40 ^{13}C NMR spectrum of 6-*O*-(4-hydroxybutyl)-6-*O*-desmethyl-diprenorphine (**29c**, HB-DPN) in CDCl$_3$

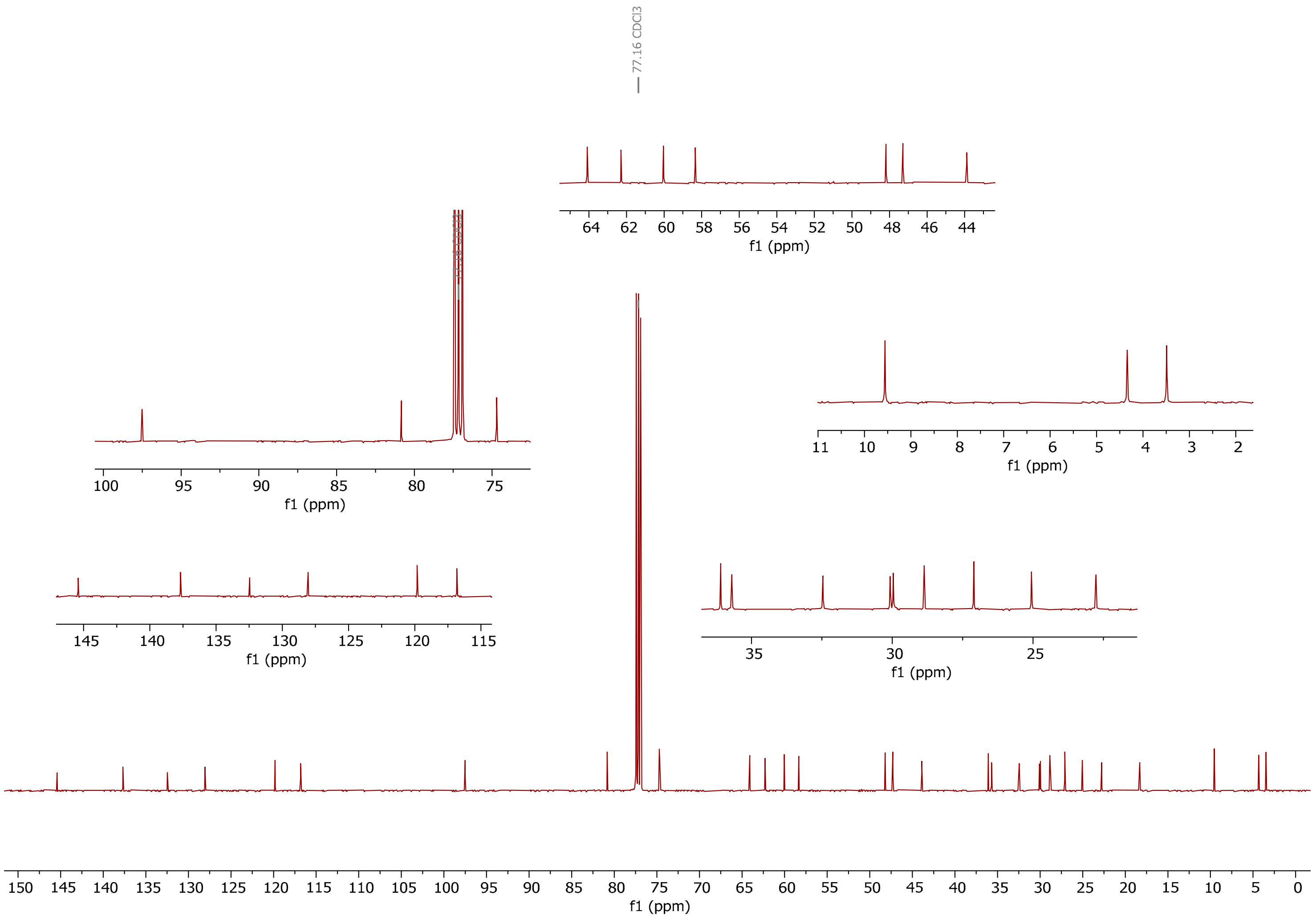

Figure S41 ^{1}H NMR spectrum of 6-*O*-(5-hydroxypentyl)-6-*O*-desmethyl-diprenorphine (**29d**, HPe-DPN) in $CDCl_3$

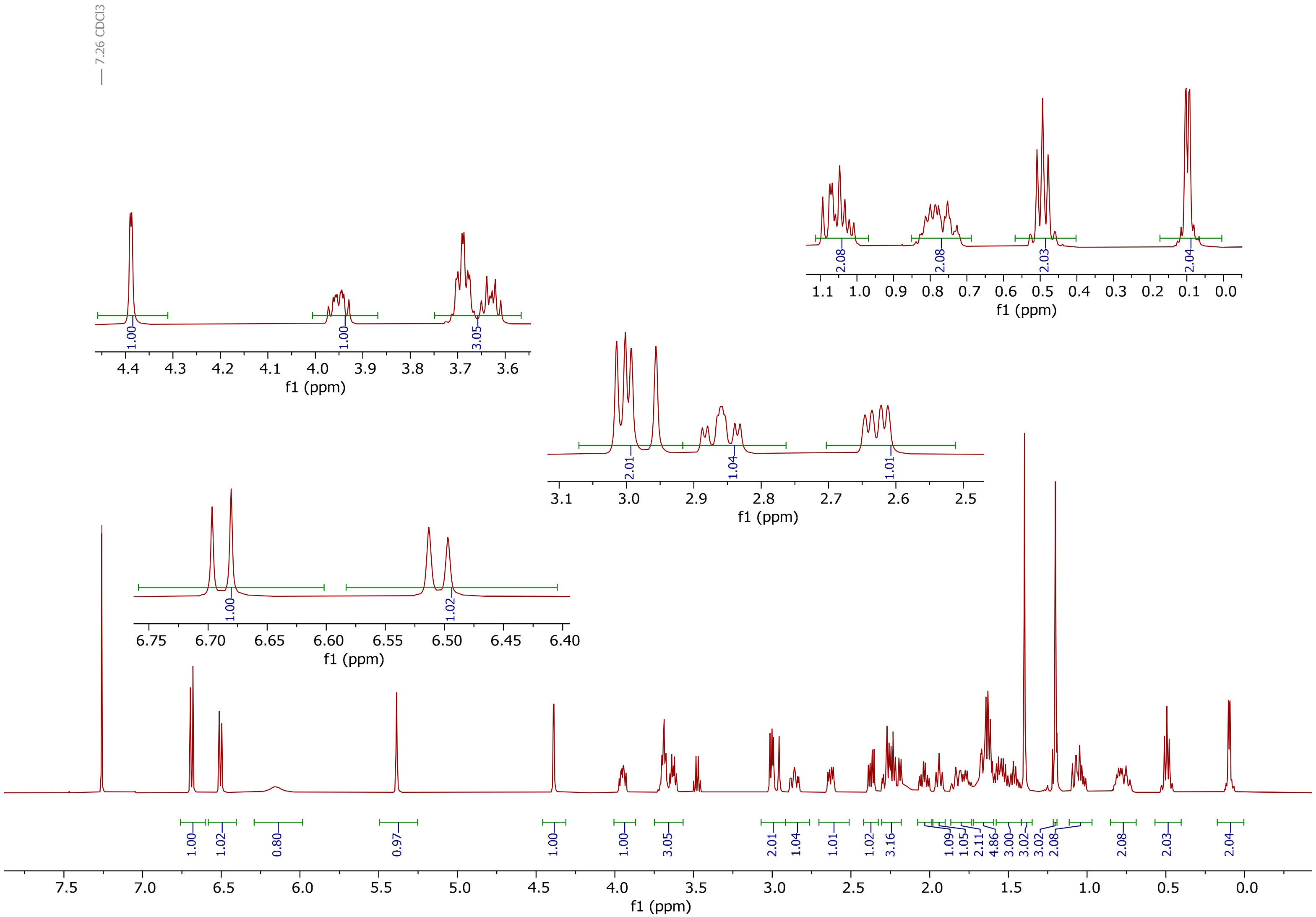

Figure S42 ^{13}C NMR spectrum of 6-*O*-(5-hydroxypentyl)-6-*O*-desmethyl-diprenorphine (**29d**, HPe-DPN) in $CDCl_3$

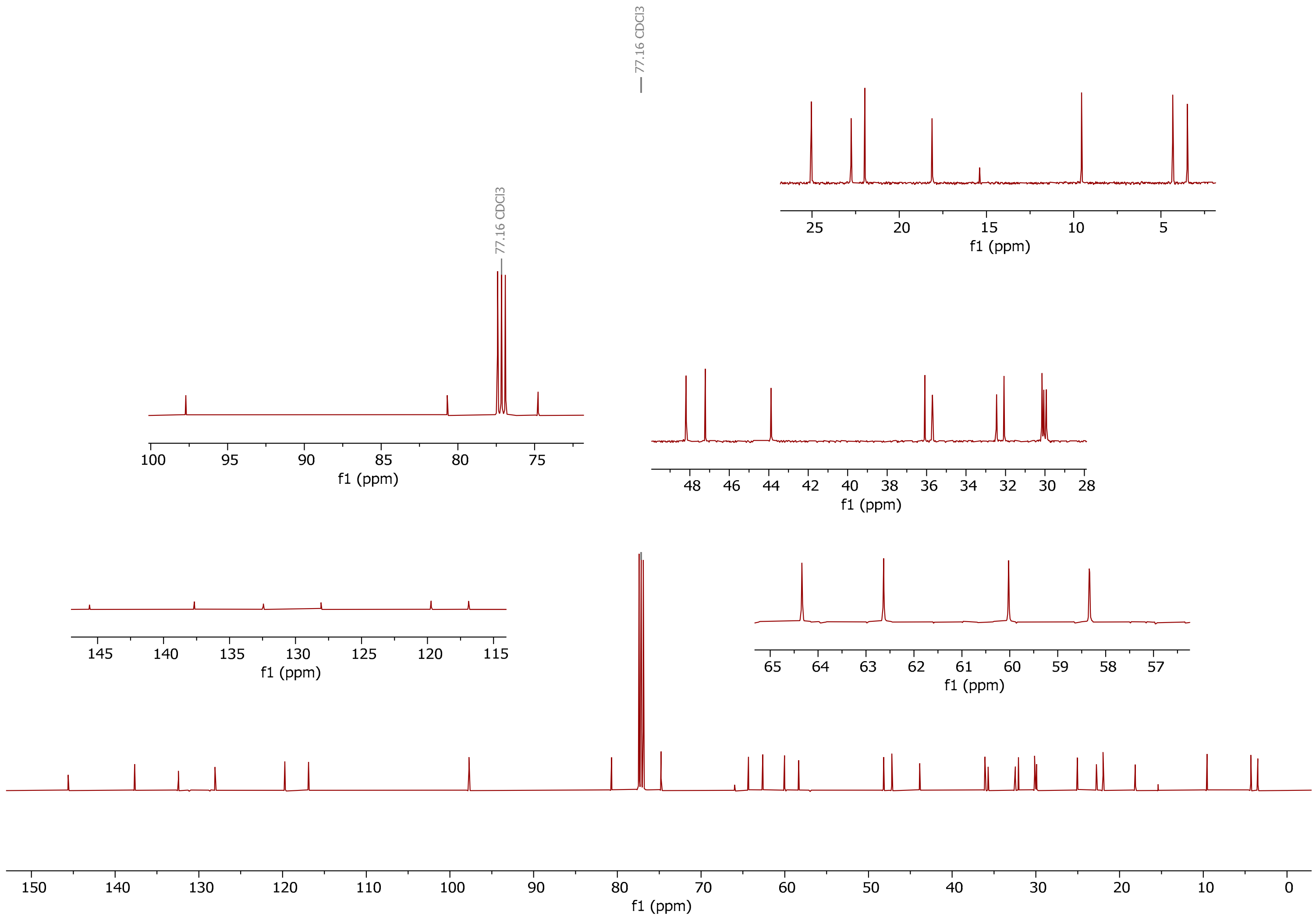
77.16 CDCl3
77.16 CDCl3
f1 (ppm)
f1 (ppm)
f1 (ppm)
f1 (ppm)
f1 (ppm)
f1 (ppm)

Figure S43 ^{1}H NMR spectrum of 3-fluoropropyl tosylate (**33a**, FPOTos) in $CDCl_3$

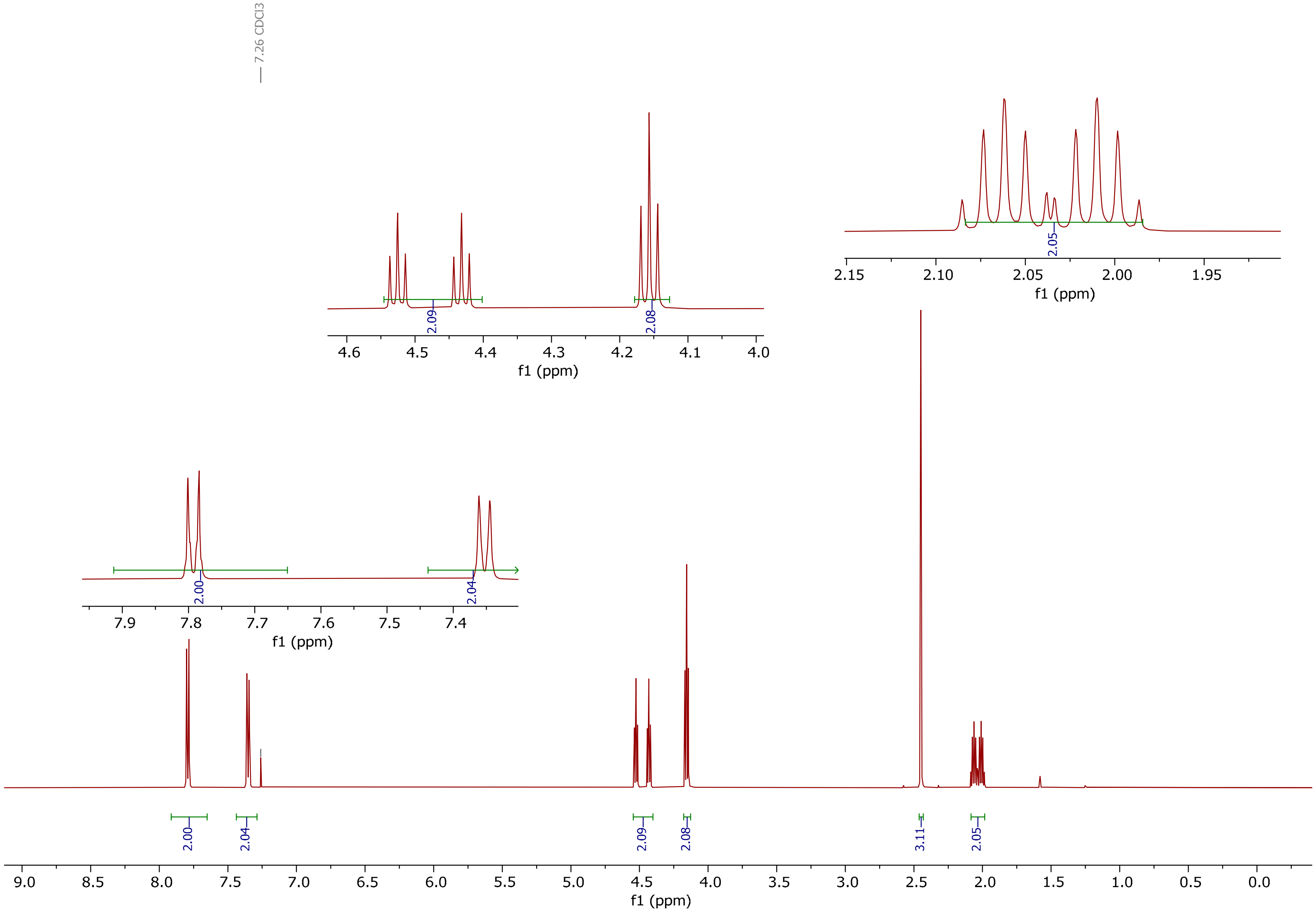

Figure S44 ^{13}C NMR spectrum of 3-fluoropropyl tosylate (**33a**, FPOTos) in $CDCl_3$

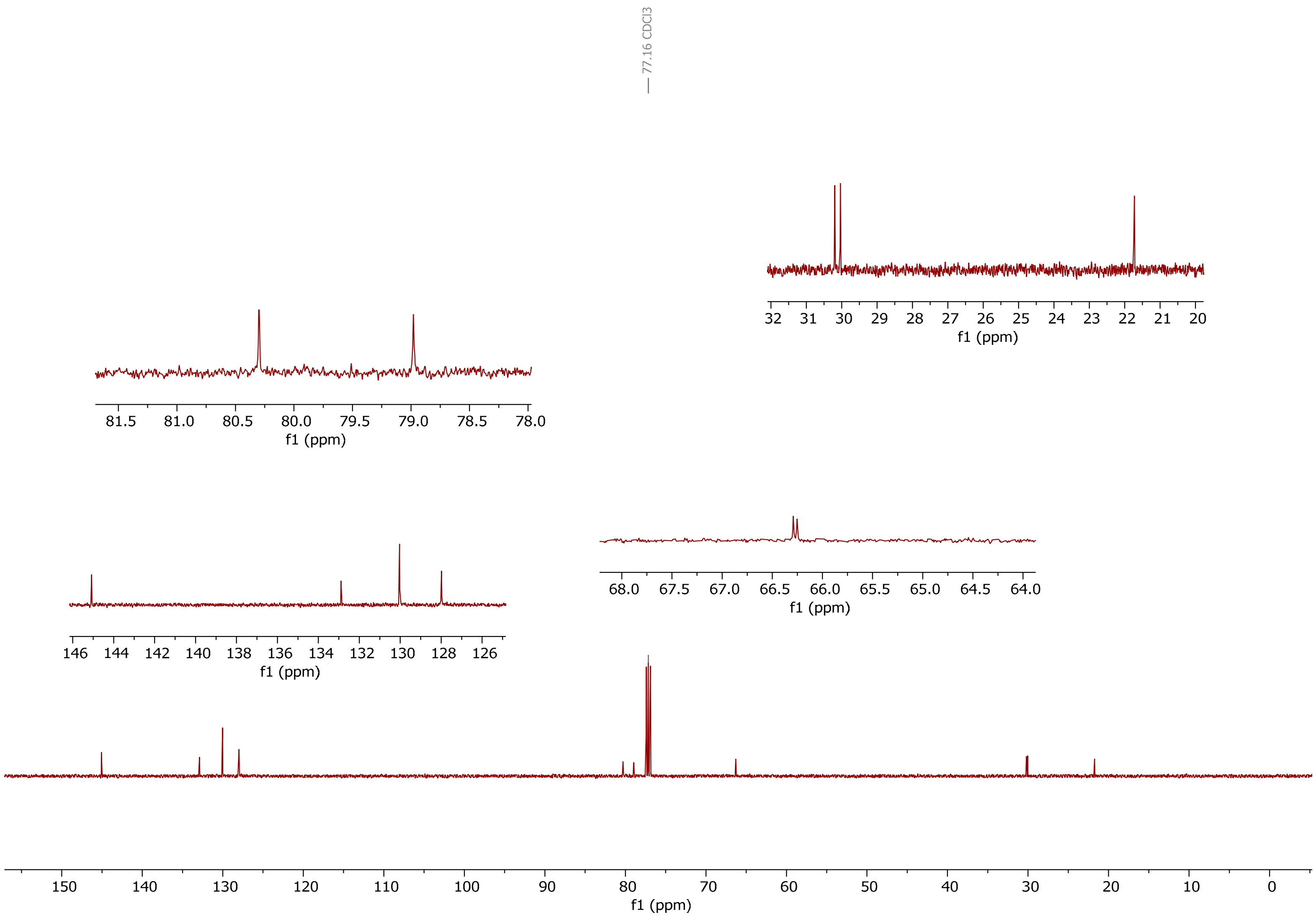

Figure S45 ^{19}F NMR spectrum of 3-fluoropropyl tosylate (**33a**, FPOTos) in $CDCl_3$

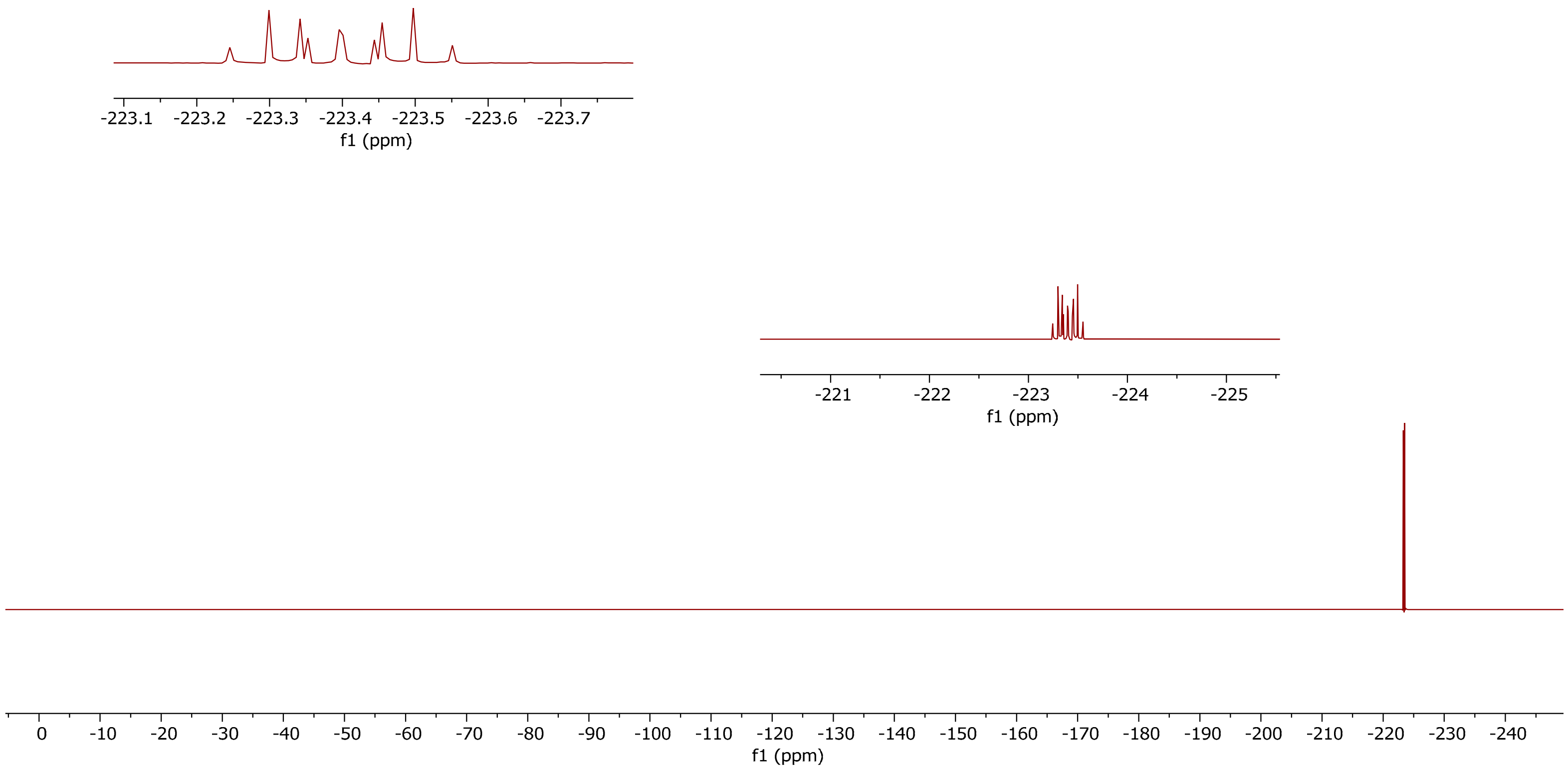

Figure S46 ^{1}H NMR spectrum of 4-fluorobutyl tosylate (**33b**, FBOTos) in $CDCl_3$

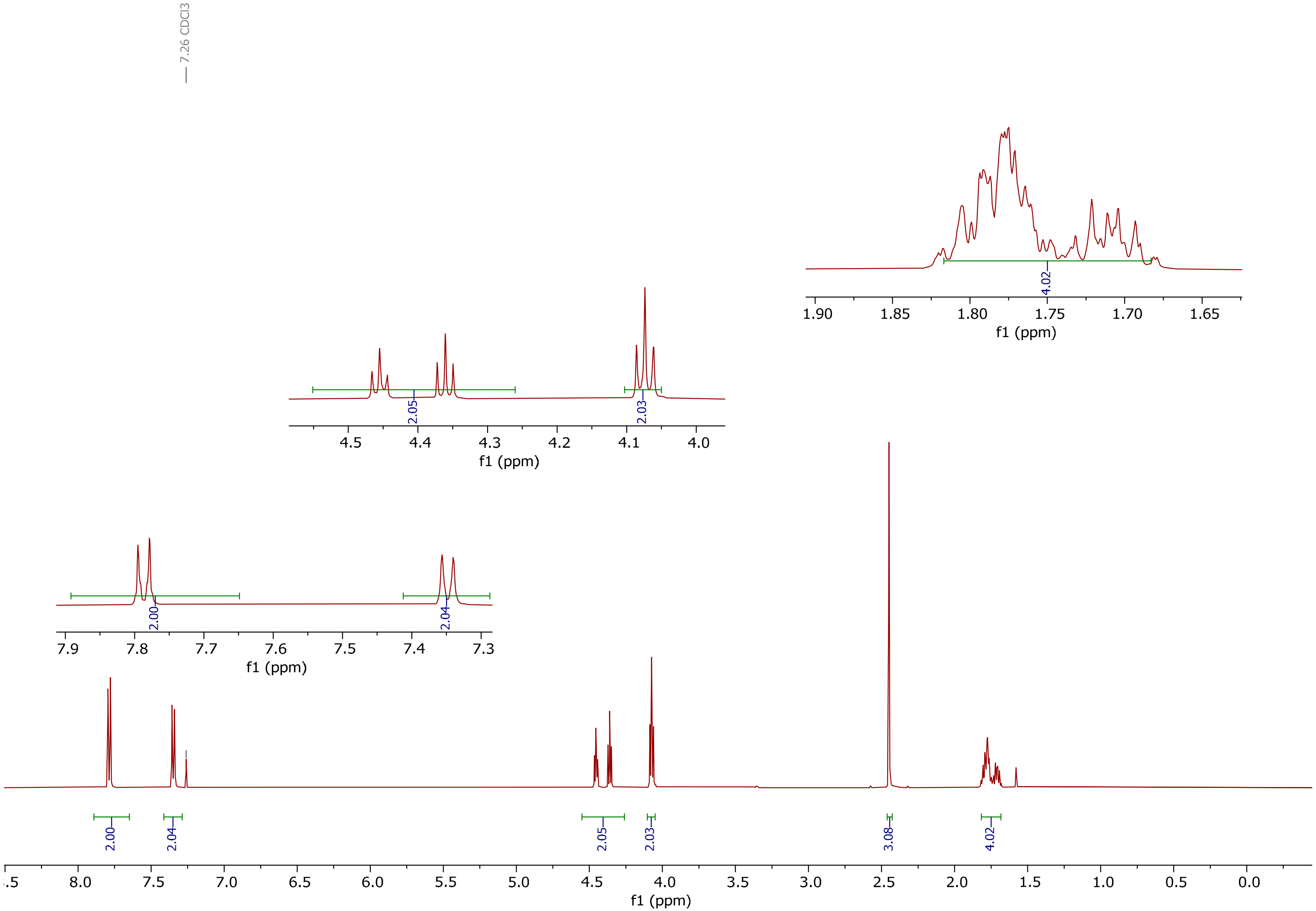

Figure S47 ^{13}C NMR spectrum of 4-fluorobutyl tosylate (**33b**, FBOTos) in $CDCl_3$

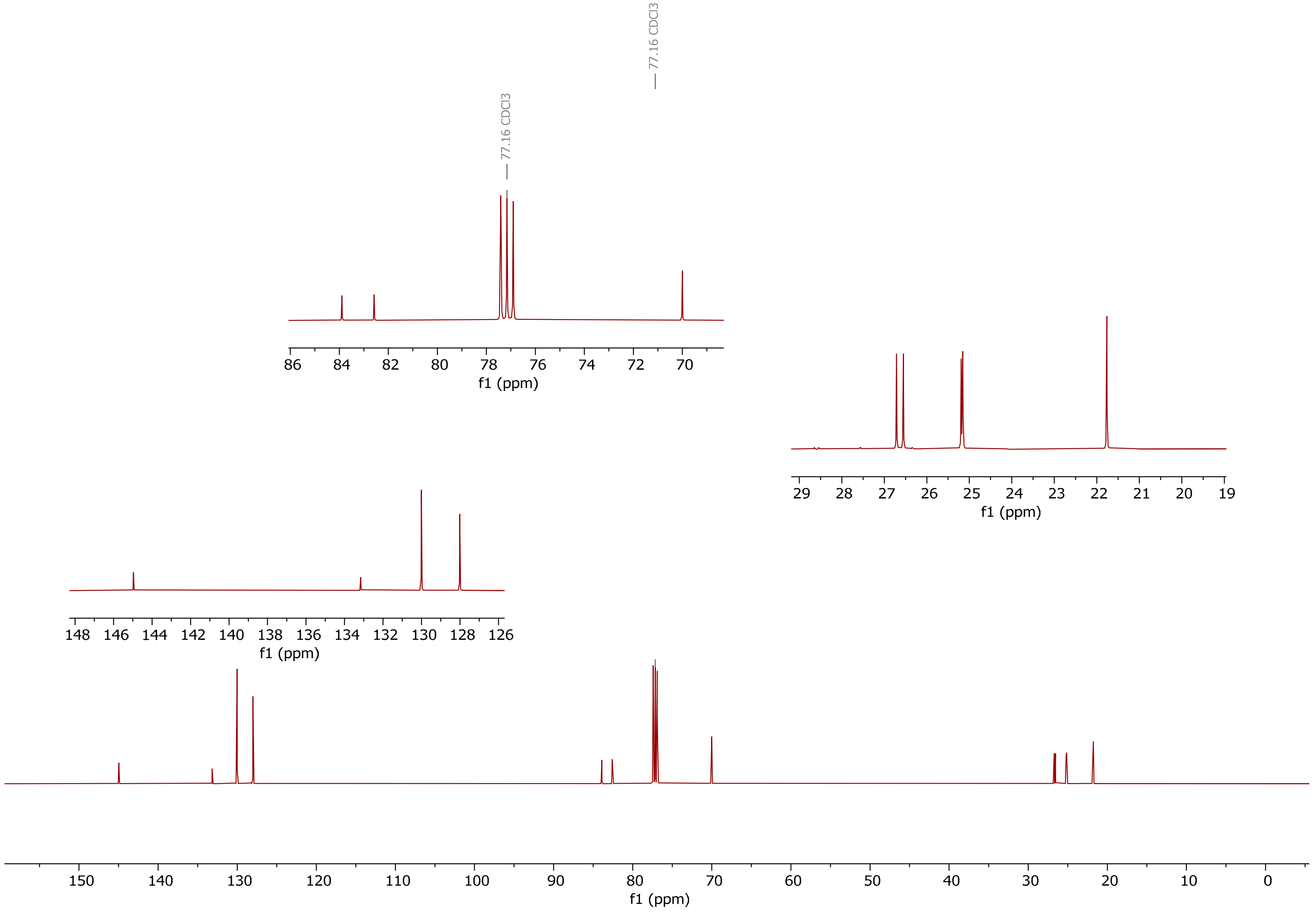

Figure S48 ^{19}F NMR spectrum of 4-fluorobutyl tosylate (**33b**, FBOTos) in $CDCl_3$

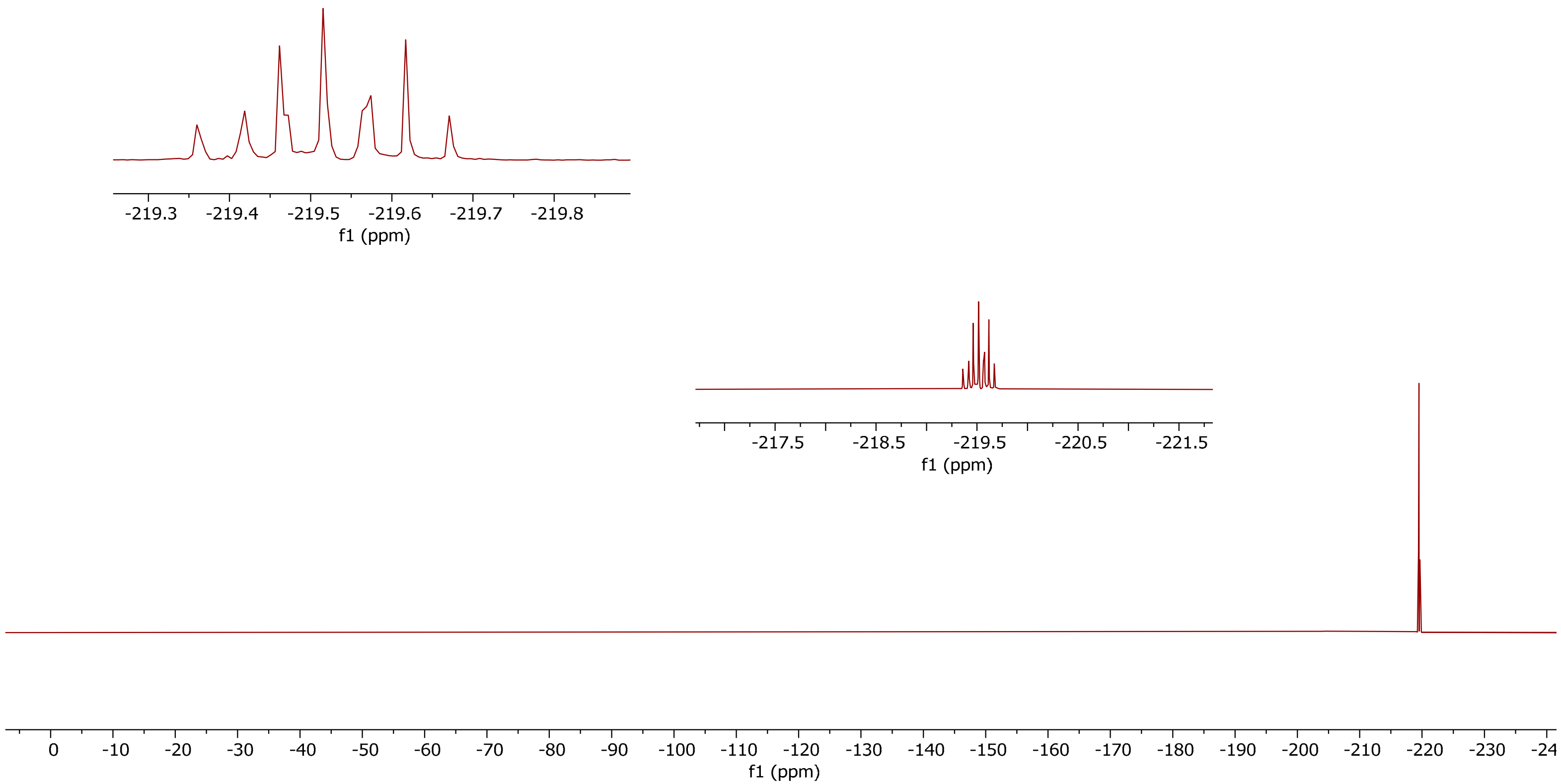

Figure S49 ^{1}H NMR spectrum of 5-fluoropentyl tosylate (**33c**, FPeOTos) in $CDCl_3$

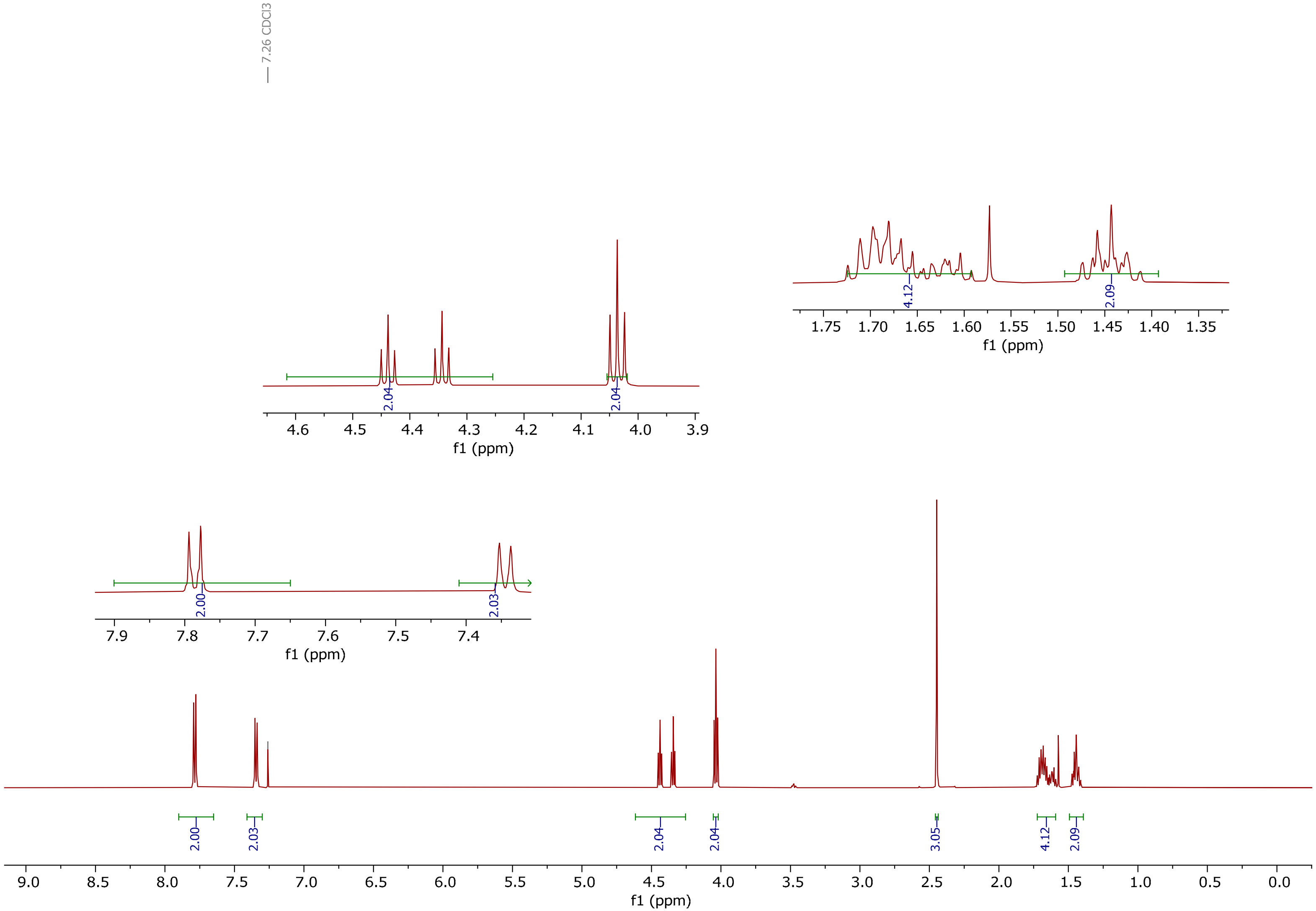

Figure S50 ^{13}C NMR spectrum of 5-fluoropentyl tosylate (**33c**, FPeOTos) in $CDCl_3$

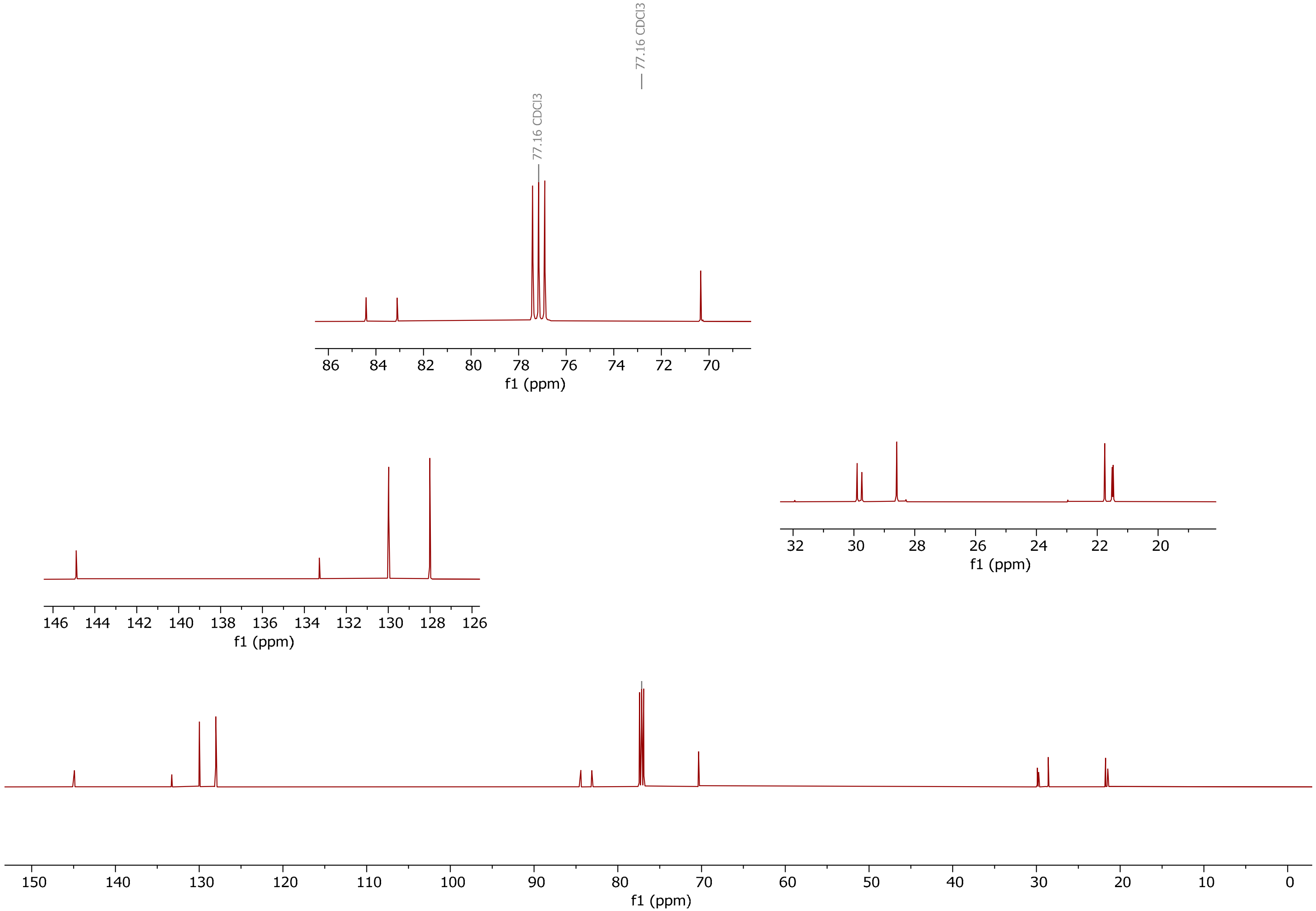

Figure S51 ^{19}F NMR spectrum of 5-fluoropentyl tosylate (**33c**, FPeOTos) in $CDCl_3$

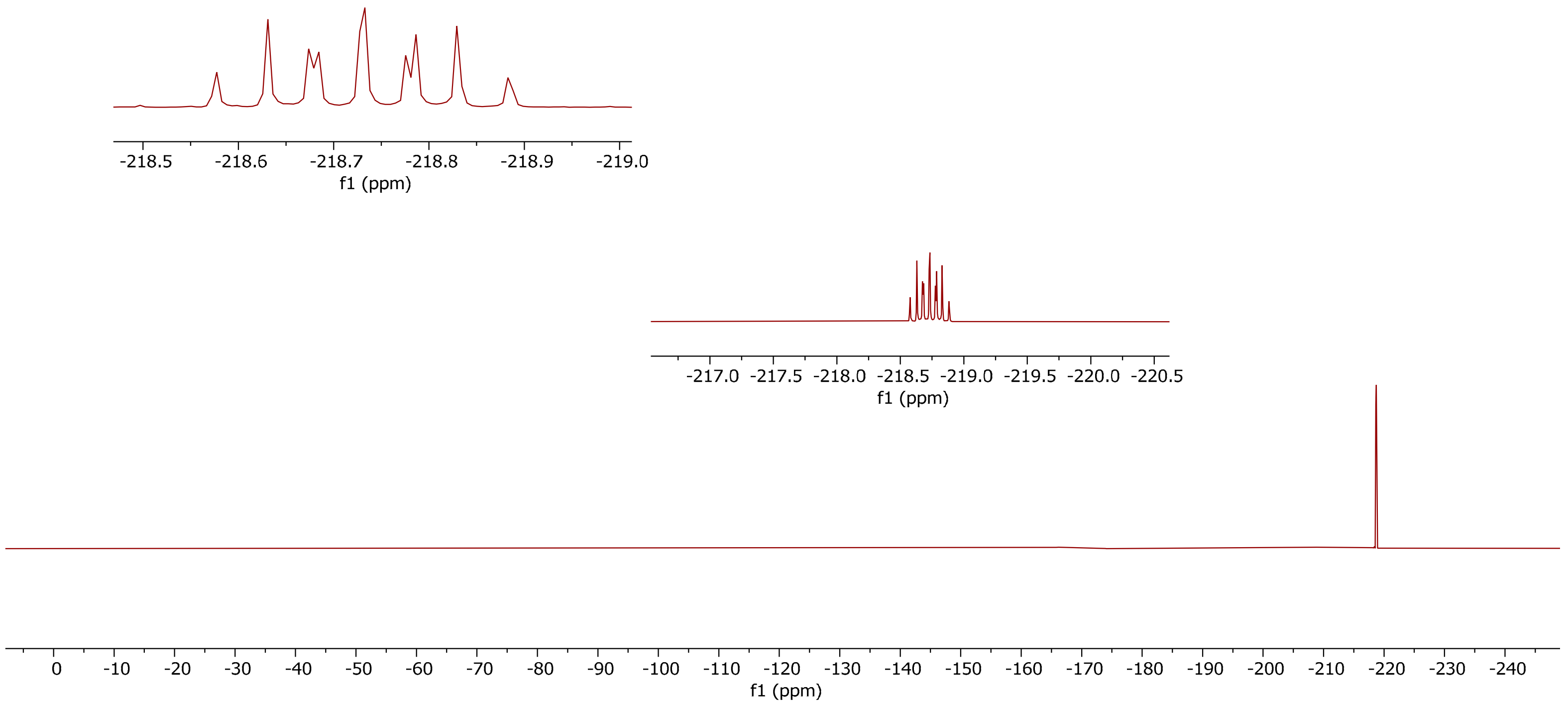

Figure S52 ^{1}H NMR spectrum of (3-bromopropoxy)-*tert*-butyldiphenylsilane (**34a**, $Br(CH_2)_3OTBDPS$) in $CDCl_3$

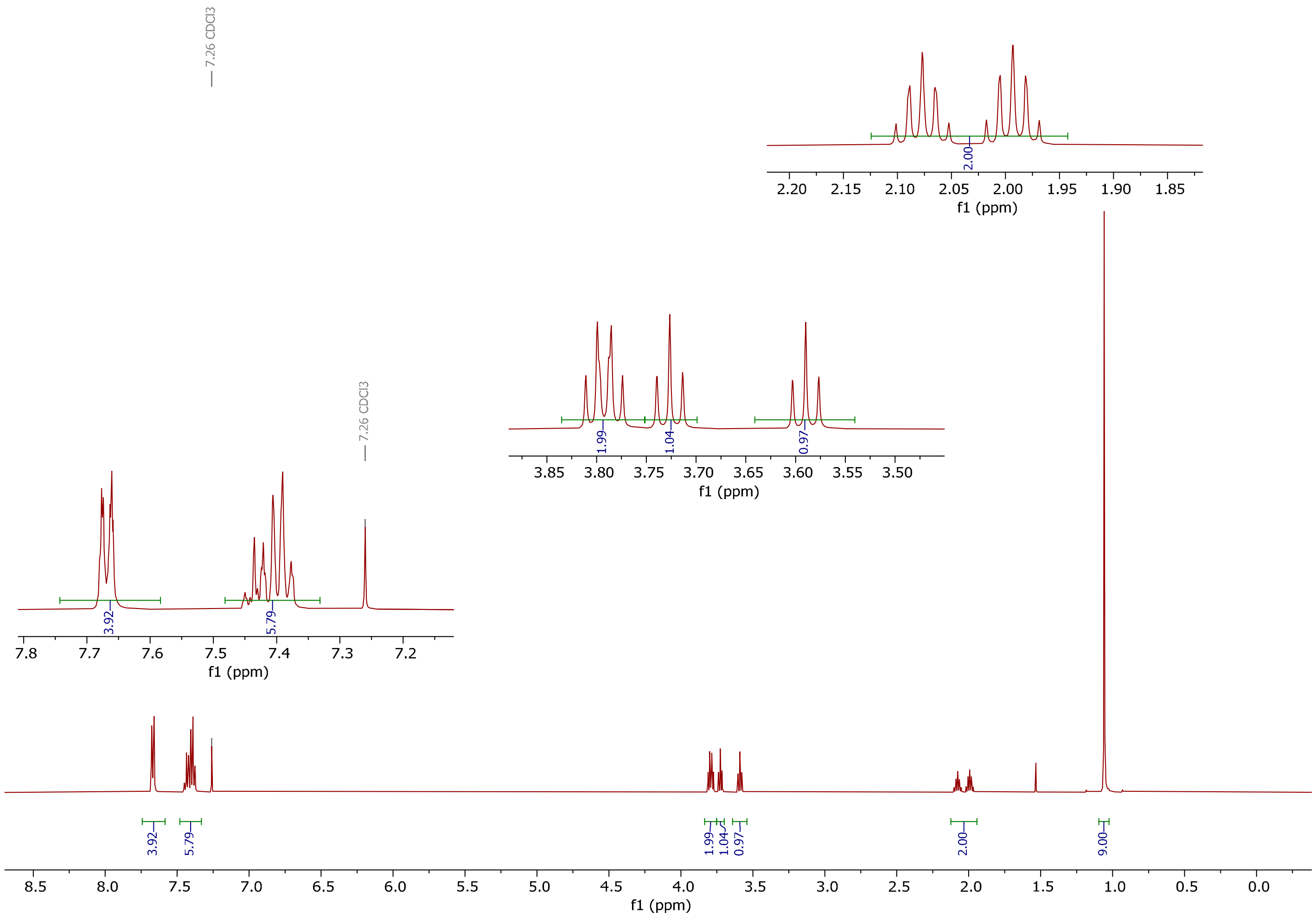

Figure S53 ^{13}C NMR spectrum of (3-bromopropoxy)-*tert*-butyldiphenylsilane (**34a**, $Br(CH_2)_3OTBDPS$) in $CDCl_3$

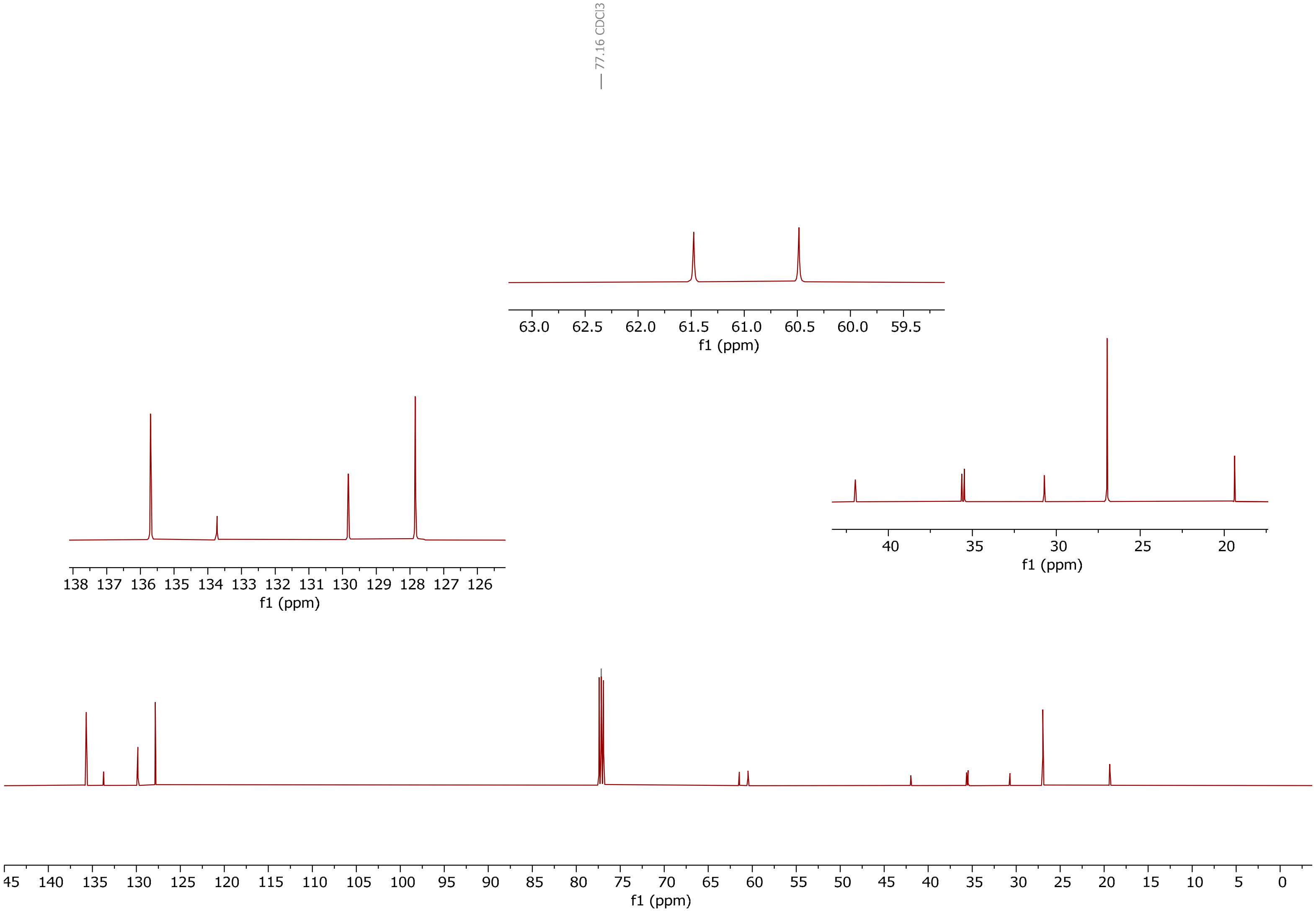

Figure S54 ^{1}H NMR spectrum of (4-bromobutoxy)-*tert*-butyldiphenylsilane (**34b**, $Br(CH_2)_4OTBDPS$) in $CDCl_3$

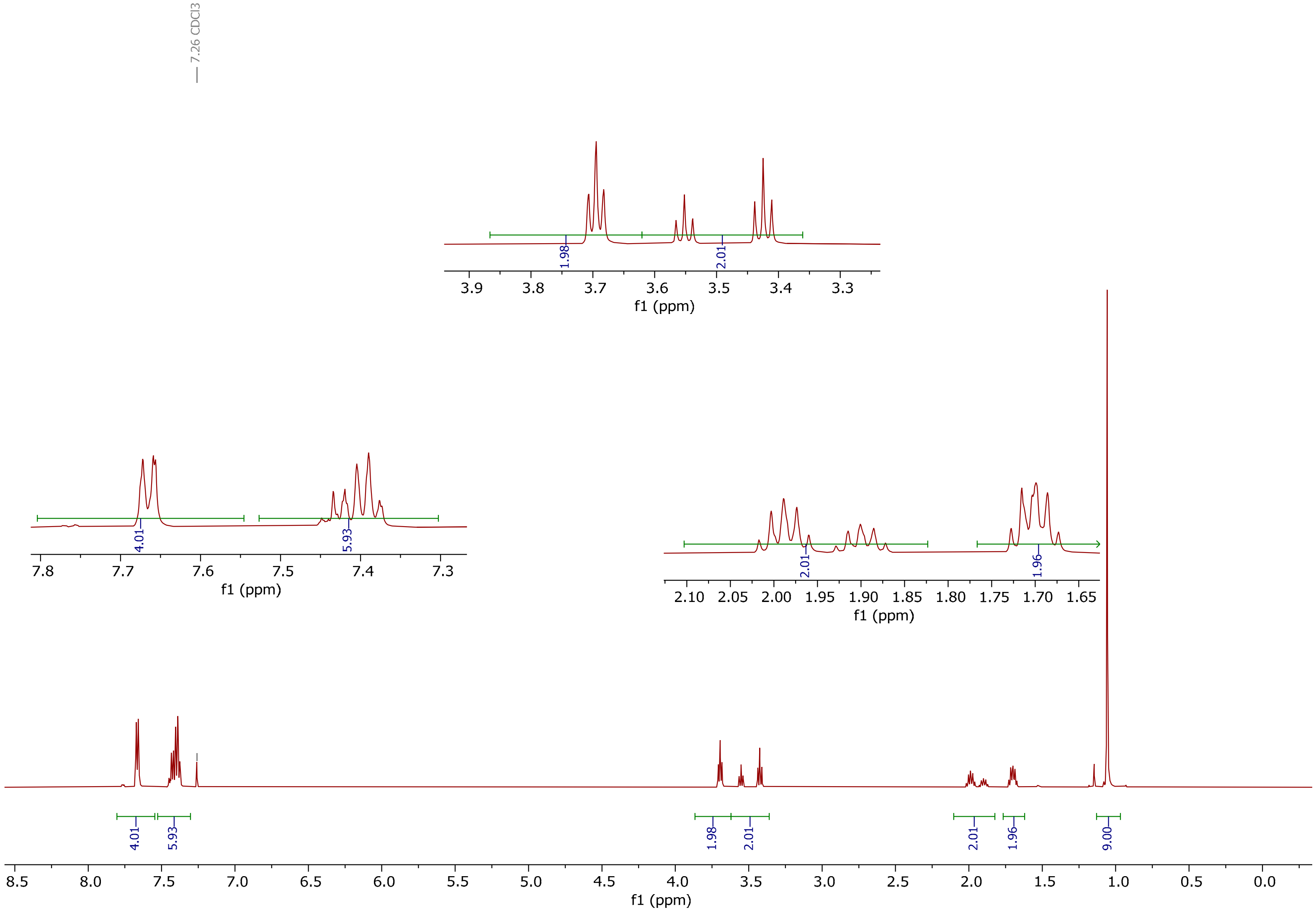

Figure S55 ^{1}H NMR spectrum of (4-bromobutoxy)-*tert*-butyldiphenylsilane (**34b**, Br(CH$_2$)$_4$OTBDPS) in CDCl$_3$

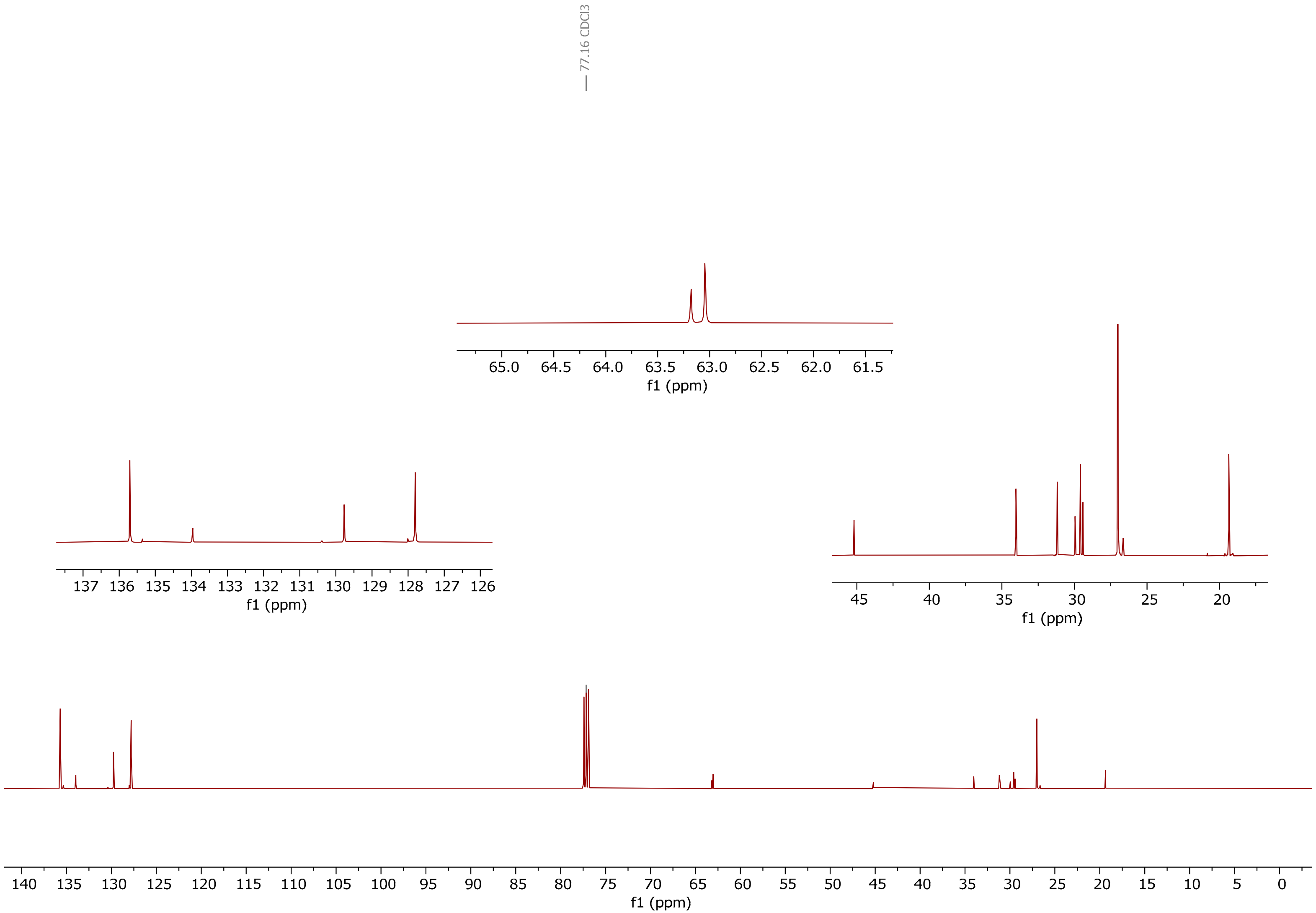

Figure S56 ^{1}H NMR spectrum of (5-bromopentoxy)-*tert*-butyldiphenylsilane (**34c**, Br$(CH_2)_5$OTBDPS) in $CDCl_3$

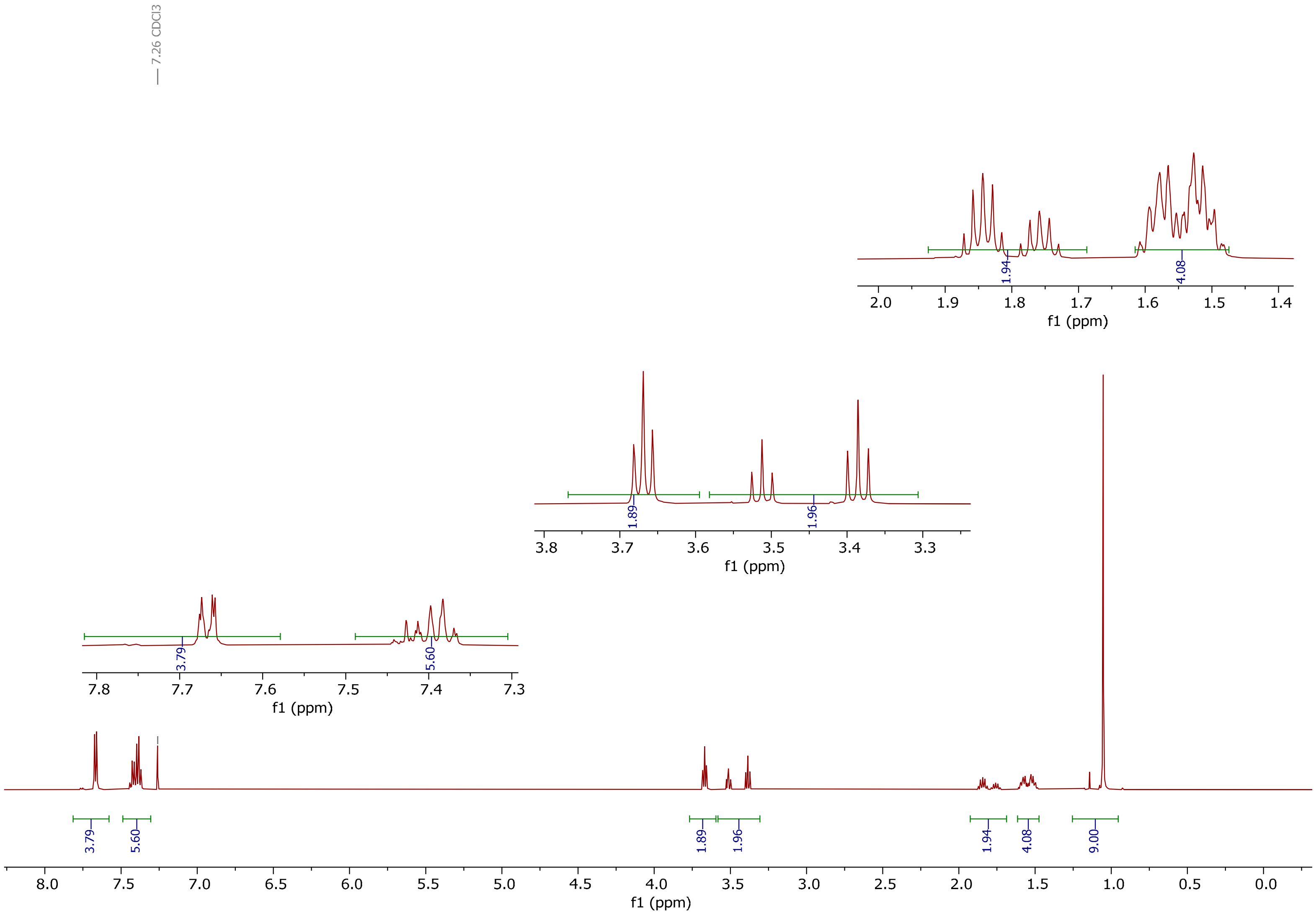

Figure S57 ^{13}C NMR spectrum of (5-bromopentoxy)-*tert*-butyldiphenylsilane (**34c**, $Br(CH_2)_5OTBDPS$) in $CDCl_3$

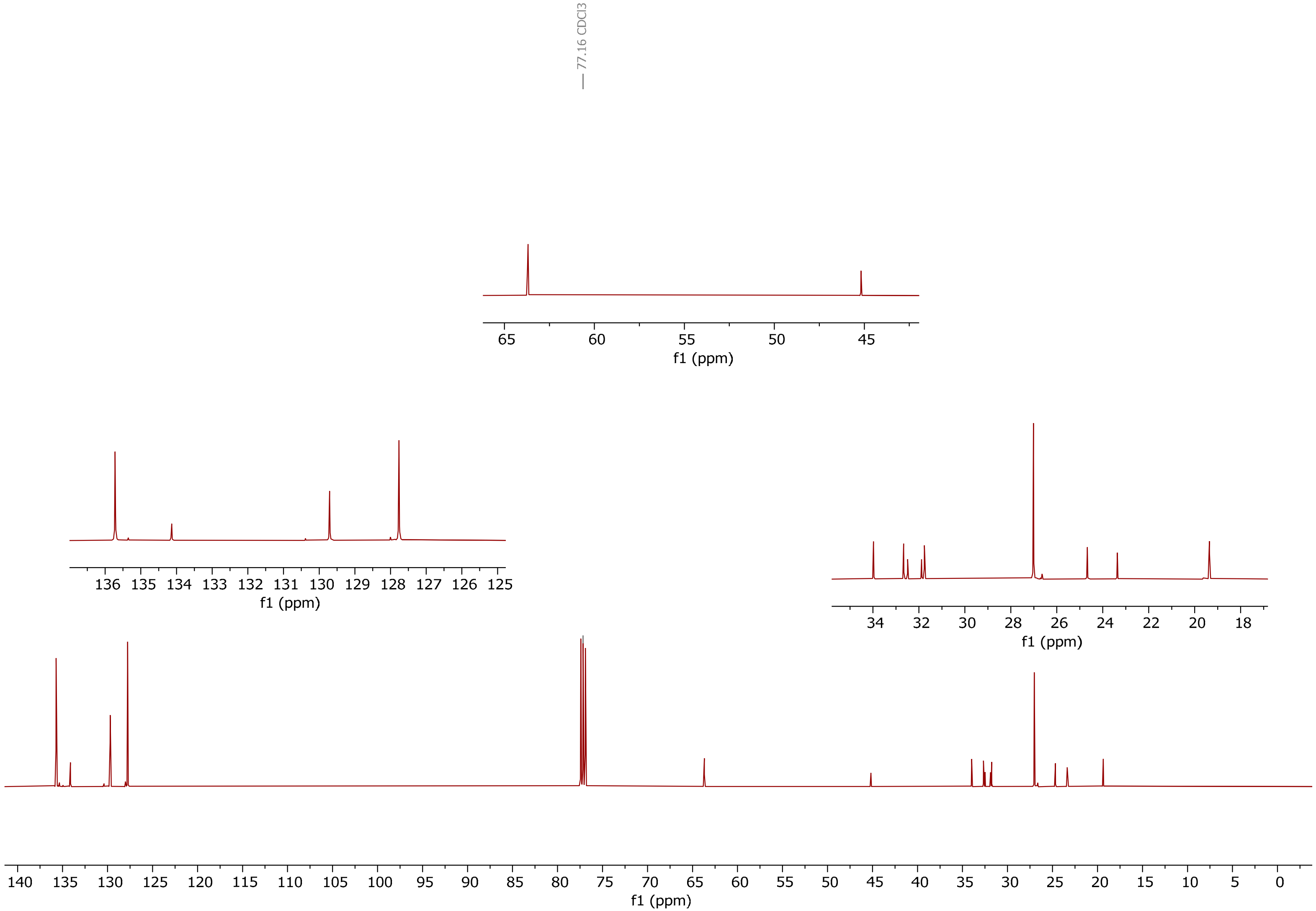

Table S3. High resolution mass spectra measurements

**Name**	**Formula**	**Error** [mDa]	**Error** [ppm]	**Theoretical MW**	**Calculated MW**	**Detected m/z**	**RT** [min]	**DBE**
TP-TDDPN (**26b**)	$C_{54}H_{59}N\ O_7S$	1.87	2.16	865.4012	865.4031	866.4104	9.74	26
TB-TDDPN (**26c**)	$C_{55}H_{61}NO_7S$	1.29	1.47	879.4169	879.4182	880.4255	9.93	26
TPe-TDDPN (**26d**)	$C_{56}H_{63}NO_7S$	0.00	0.83	893.4325	893.4333	894.4405	10.12	26
FP-DPN (**28b**)	$C_{28}H_{38}FNO_4$	0.81	1.71	471.2785	471.2793	472.2866	6.02	11
FB-DPN (**28c**)	$C_{29}H_{40}FNO_4$	0.89	1.84	485.2941	485.2950	486.3023	6.42	11
FPe-DPN (**28d**)	$C_{30}H_{42}FNO_4$	0.92	1.83	499.3098	499.3107	500.3180	6.71	11
HP-DPN (**29b**)	$C_{28}H_{39}NO_5$	0.59	1.26	469.2828	469.2834	470.2907	5.23	10
HB-DPN (**29c**)	$C_{29}H_{41}NO_5$	0.78	1.61	483.2985	483.2993	484.3065	5.49	10
HPe-DPN (**29d**)	$C_{30}H_4NO_5$	0.71	1.42	497.3141	497.3148	498.3221	5.82	10

Name – Custom name

Formula – Assigned elemental composition

Error [mDa] – Difference between measured and theoretical molecular weight of assigned annotation in Da

Error [ppm] - Difference between measured and theoretical molecular weight of assigned annotation in ppm

Theoretical MW – Theoretical molecular weight of assigned annotation

Calcualted MW – Neutral mass in Da retrieved from the measured left most isotopes of related compounds per file

Detected m/z - m/z value of the leftmost isotopic peak of most common adduct ion for this compound across measurements (area-weight average)

RT – Retention Time in min

DBE - Double Bond Equivalent

Figure S58 High Resolution Mass Spectrum of 6-*O*-(3-tosyloxypropyl)-6-*O*-desmethyl-3-*O*-trityl-diprenorphine (**26b**, TP-TDDPN)

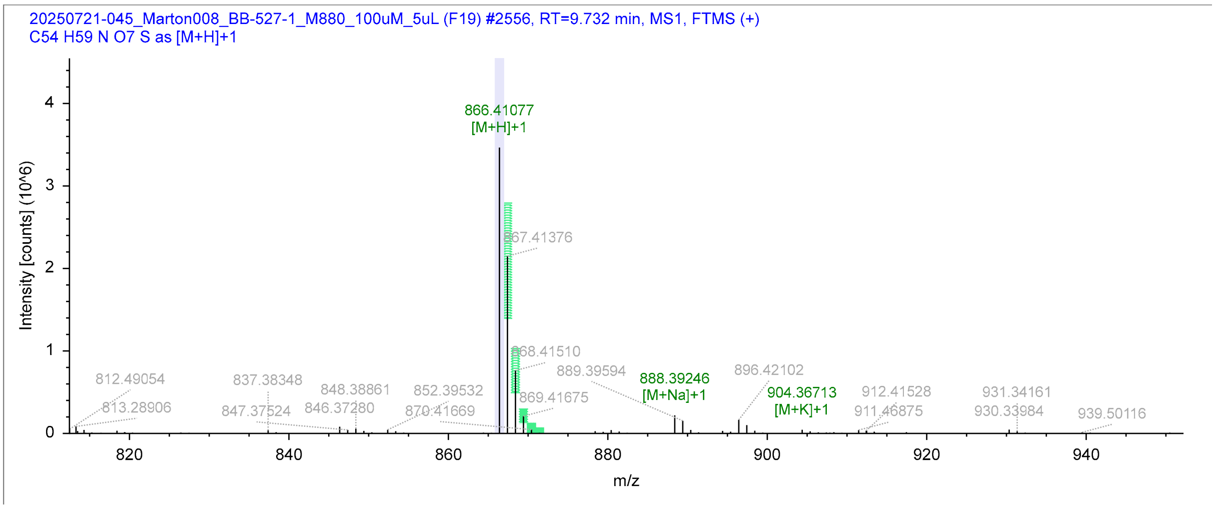

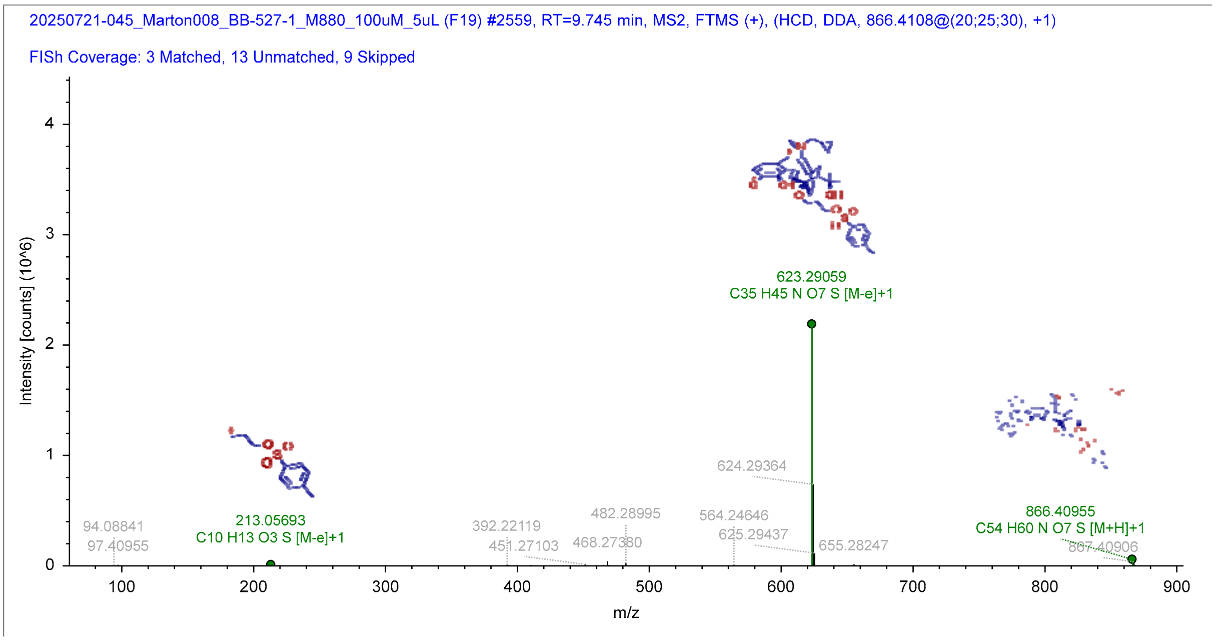

Figure S59 High Resolution Mass Spectrum of 6-*O*-(4-tosyloxybutyl)-6-*O*-desmethyl-3-*O*-trityl-diprenorphine (**26c**, TB-TDDPN)

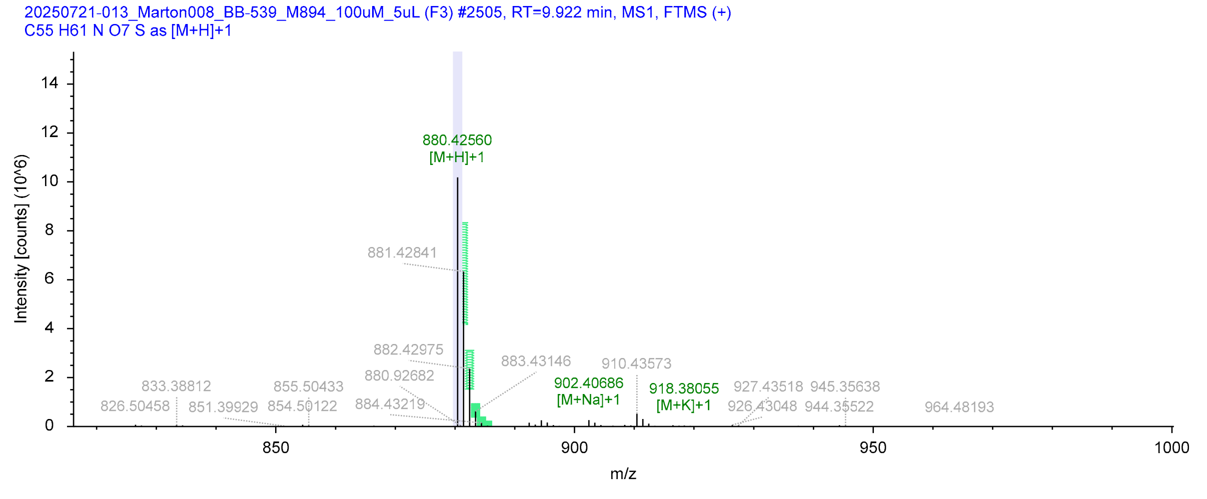

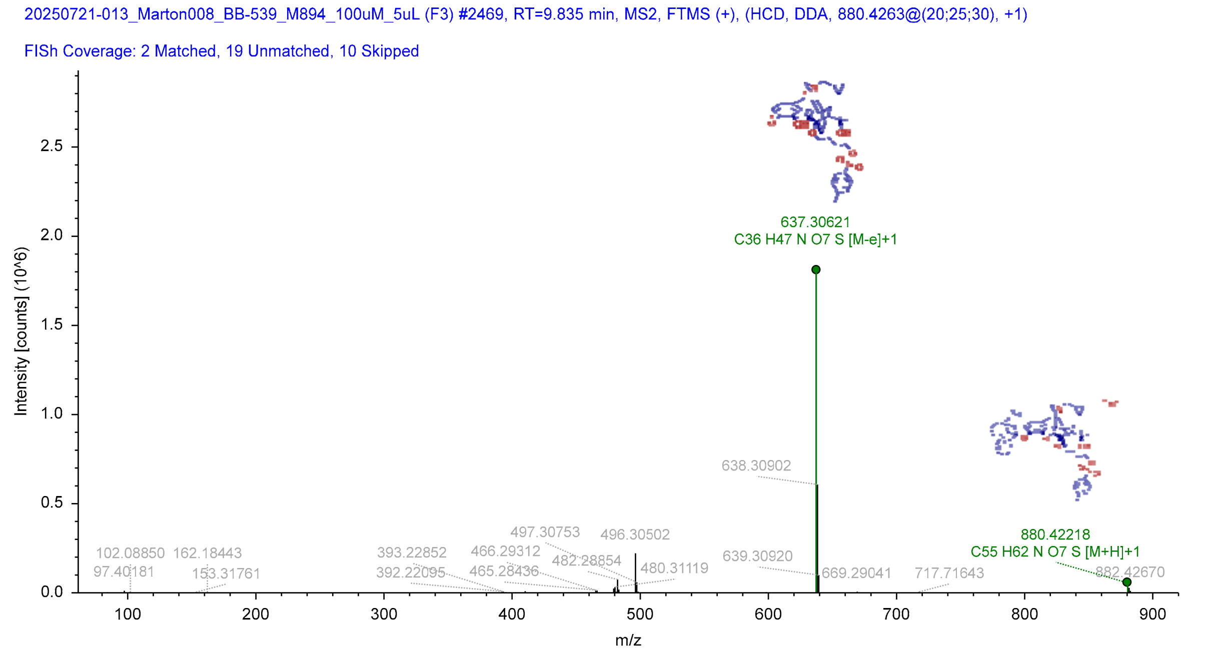

Figure S60 High Resolution Mass Spectrum of 6-*O*-(5-tosyloxypentyl)-6-*O*-desmethyl-3-*O*-trityl-diprenorphine (**26d**, TPe-TDDPN)

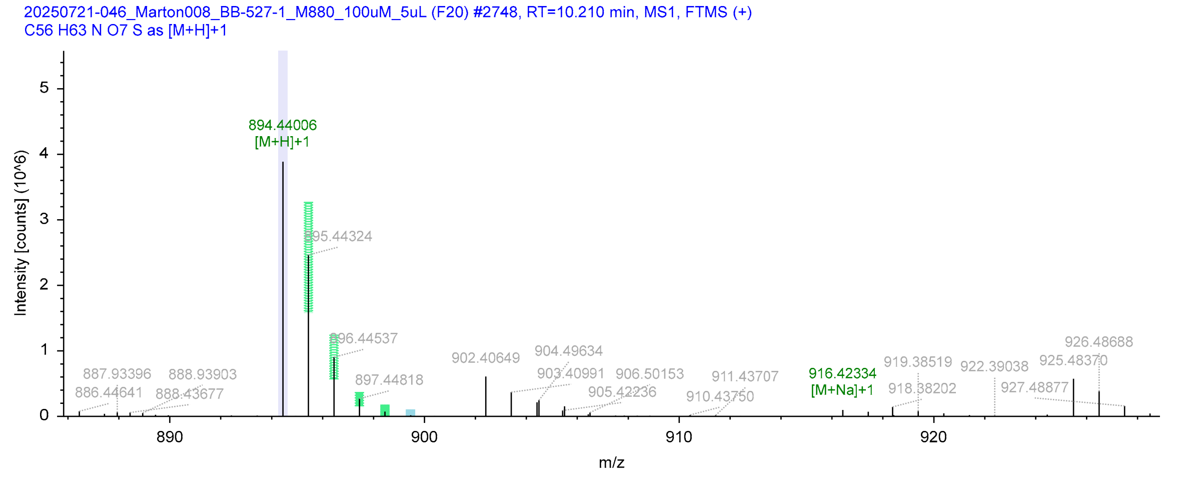

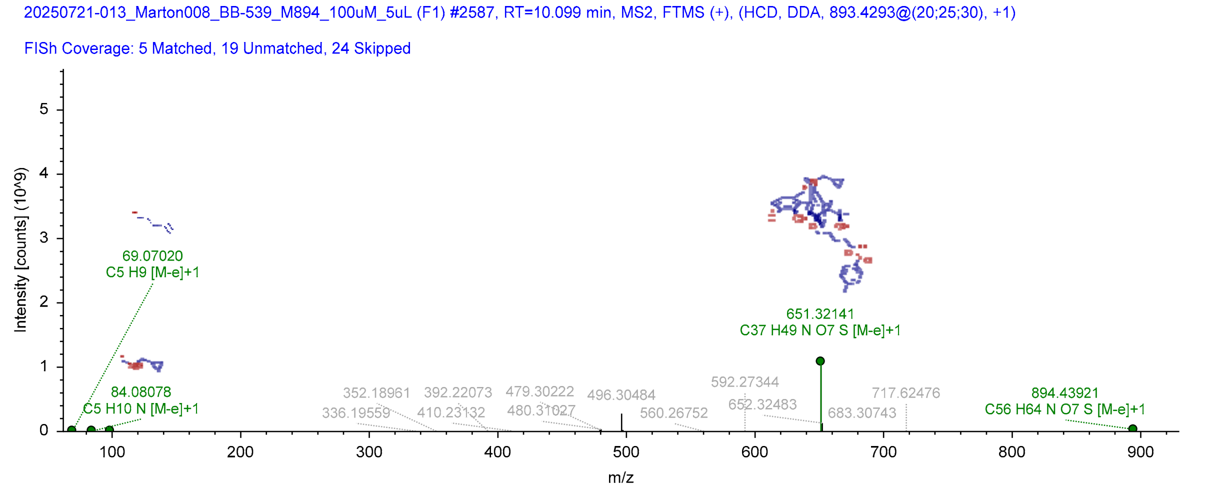

Figure S61 High Resolution Mass Spectrum of 6-*O*-(3-fluoropropyl)-6-*O*-desmethyl-diprenorphine (**28b**, FP-DPN)

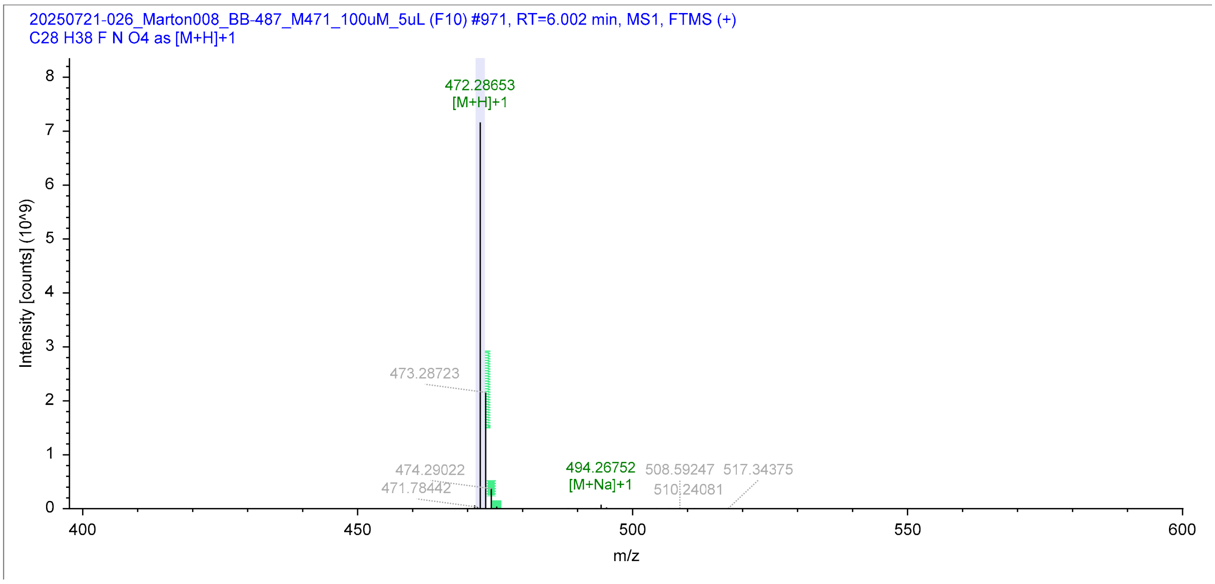

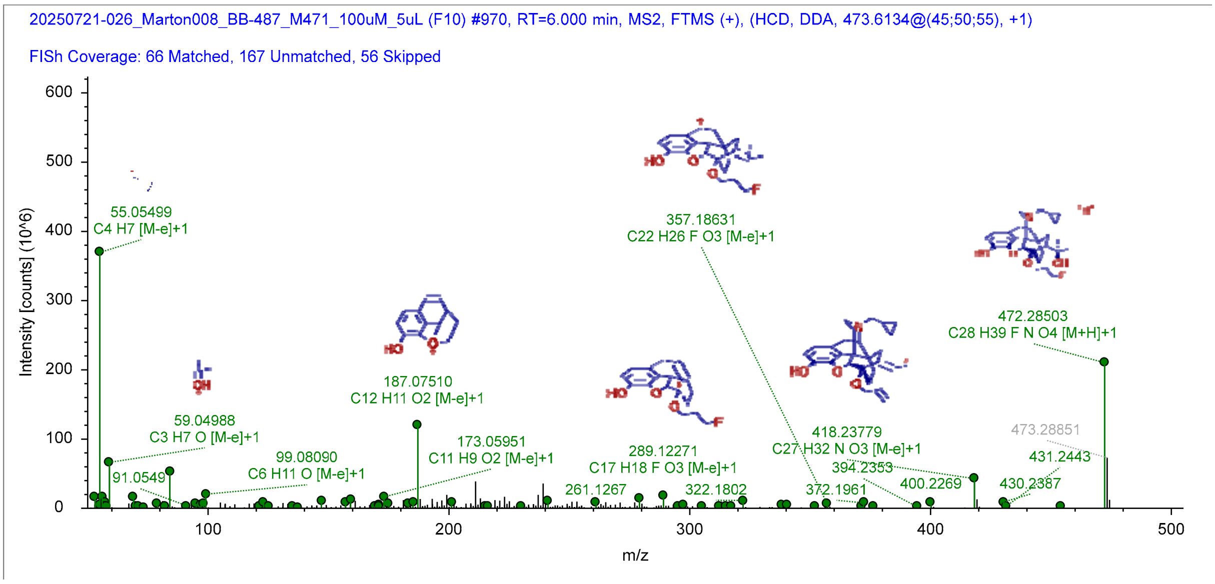

Figure S62 High Resolution Mass Spectrum of 6-*O*-(4-fluorobutyl)-6-*O*-desmethyl-diprenorphine (**28c**, FB-DPN)

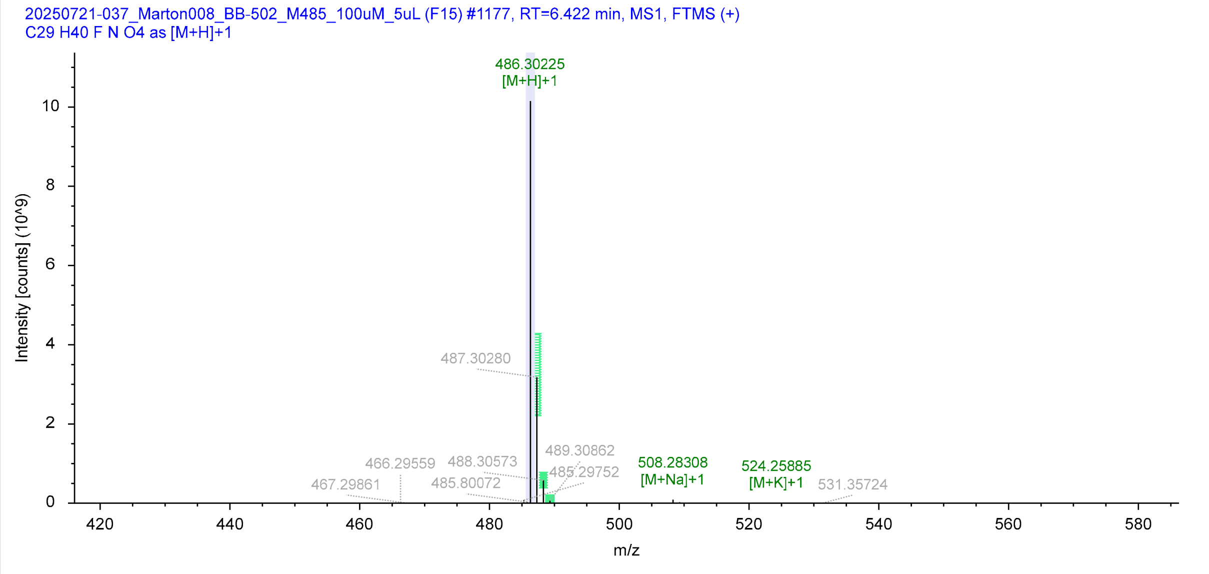

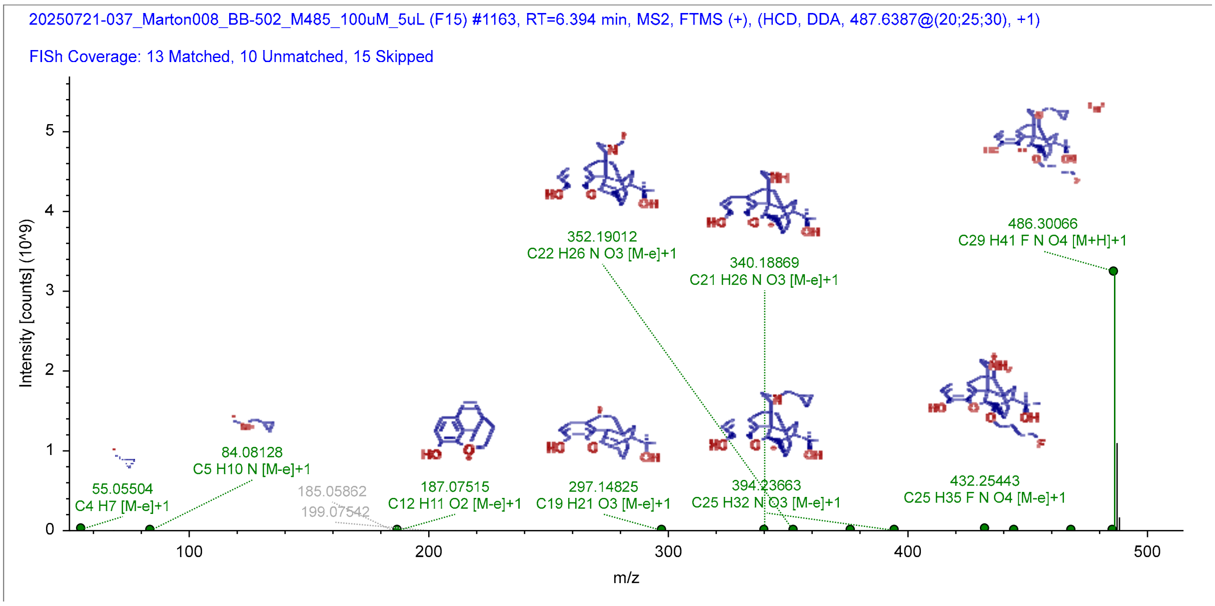

Figure S63 High Resolution Mass Spectrum of 6-*O*-(5-fluoropentyl)-6-*O*-desmethyl-diprenorphine (**28d**, FPe-DPN)

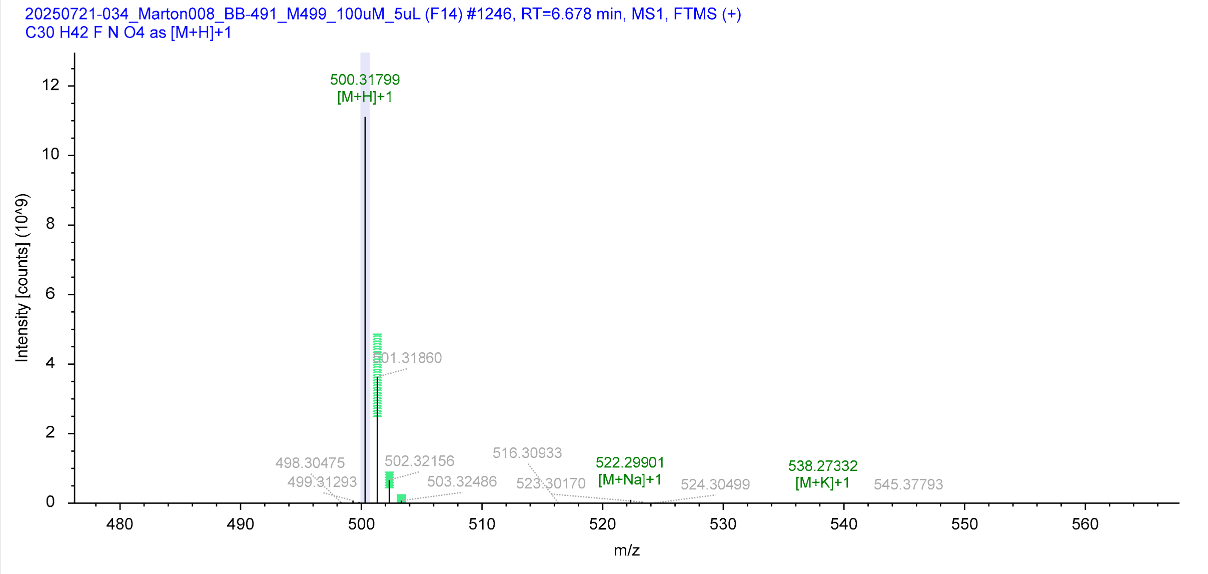

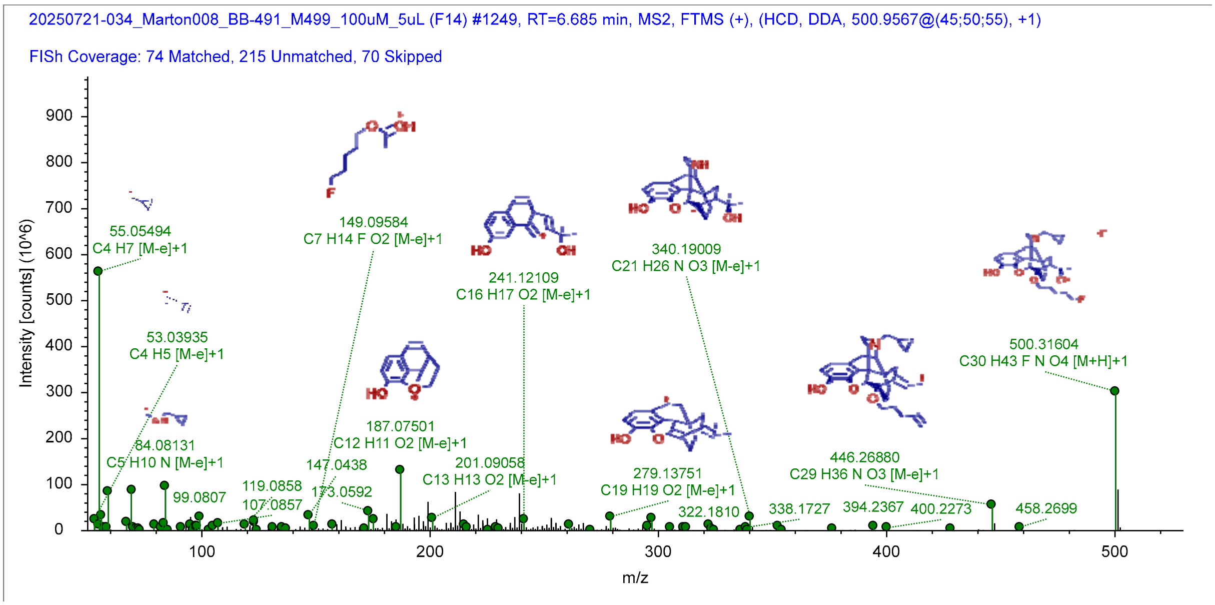

Figure S64 High Resolution Mass Spectrum of 6-*O*-(3-hydroxypropyl)-6-*O*-desmethyl-diprenorphine (**29b**, HP-DPN)

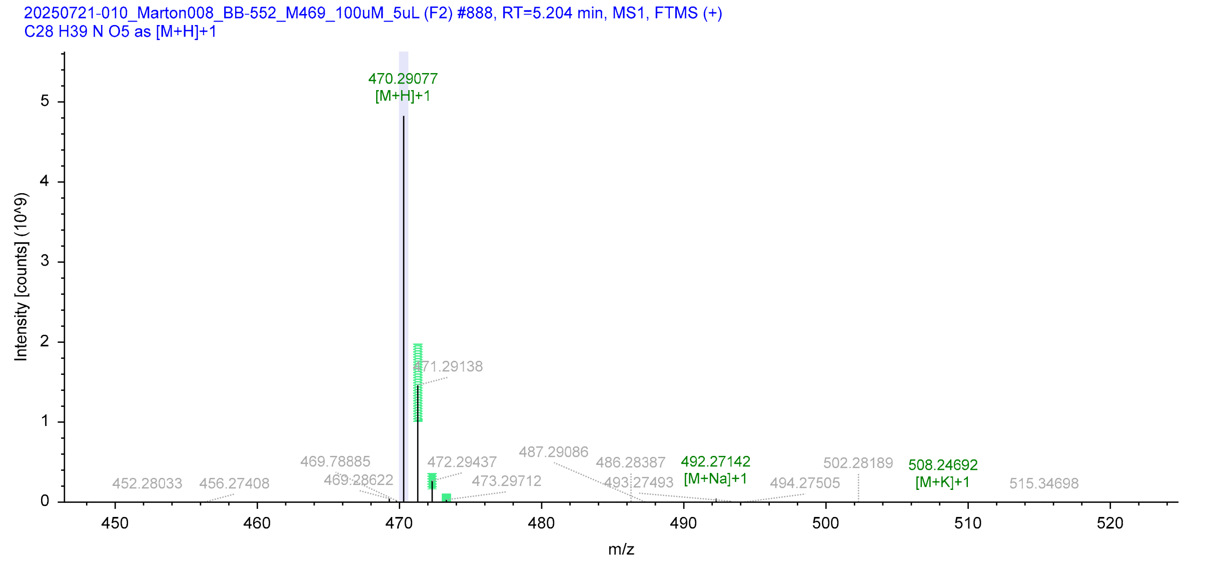

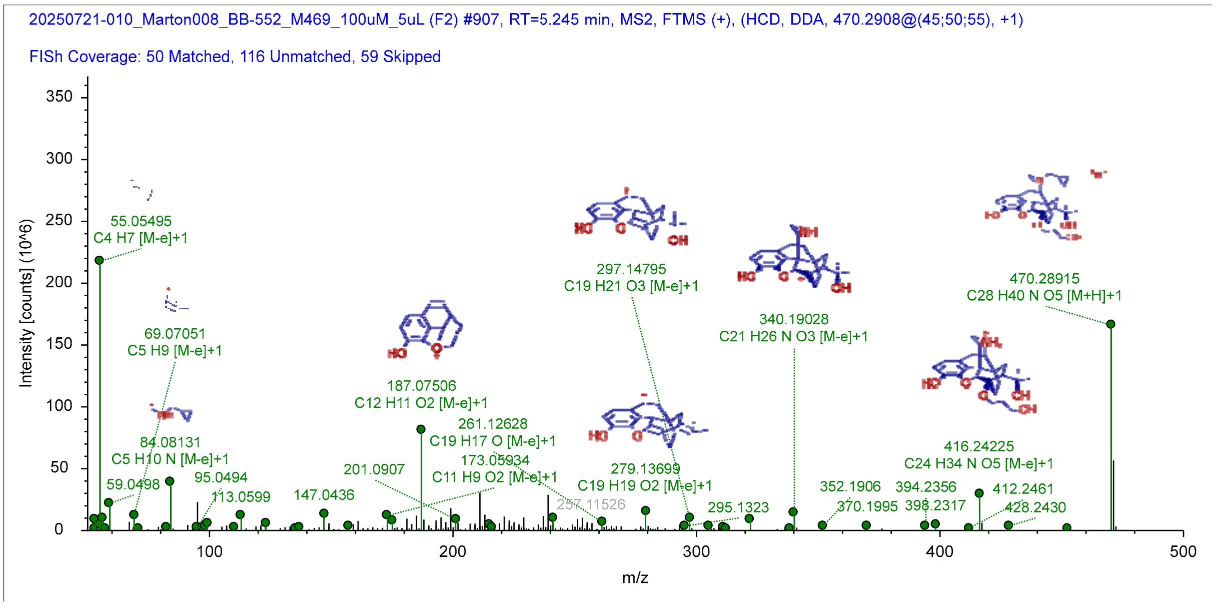

Figure S65 High Resolution Mass Spectrum of 6-*O*-(4-hydroxybutyl)-6-*O*-desmethyl-diprenorphine (**29c**, HB-DPN)

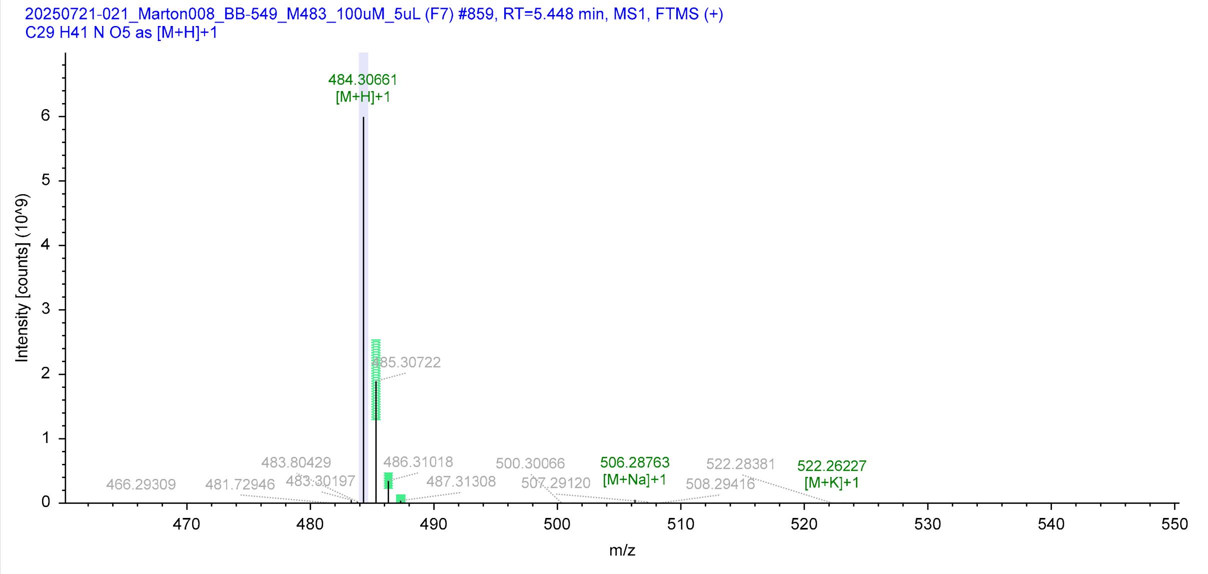

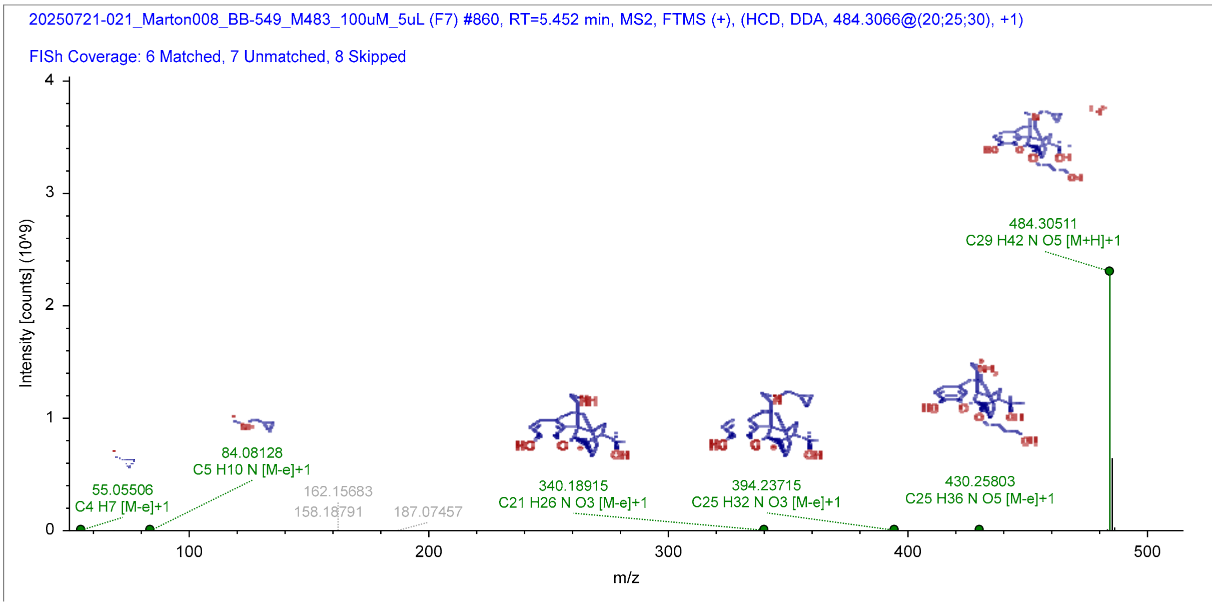

Figure S66 High Resolution Mass Spectrum of 6-*O*-(5-hydroxypentyl)-6-*O*-desmethyl-diprenorphine (**29d**, HPe-DPN)

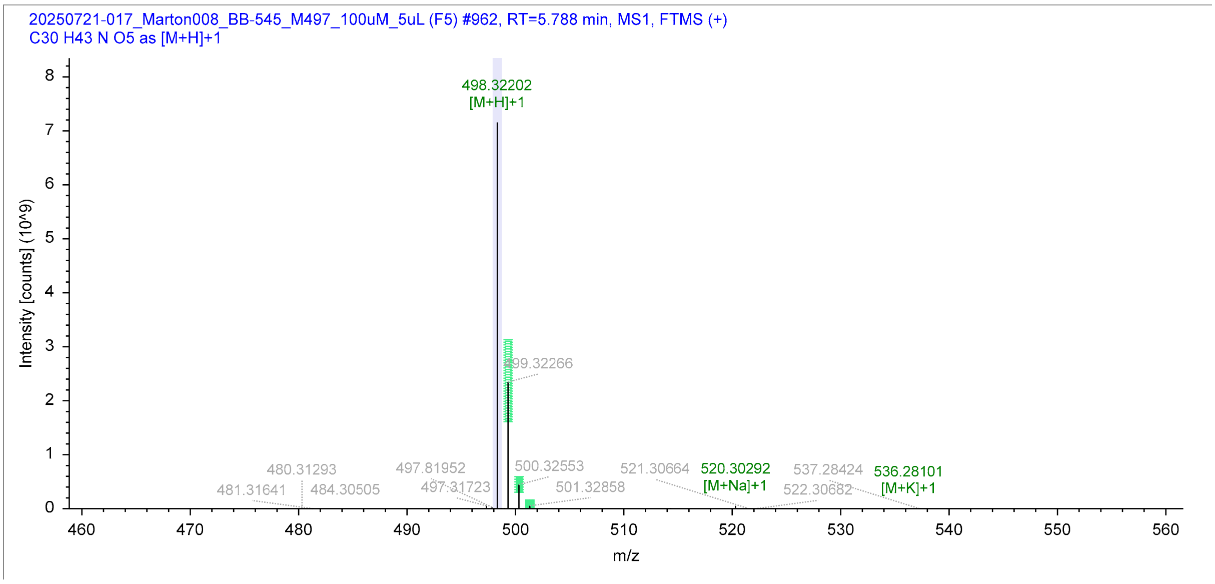

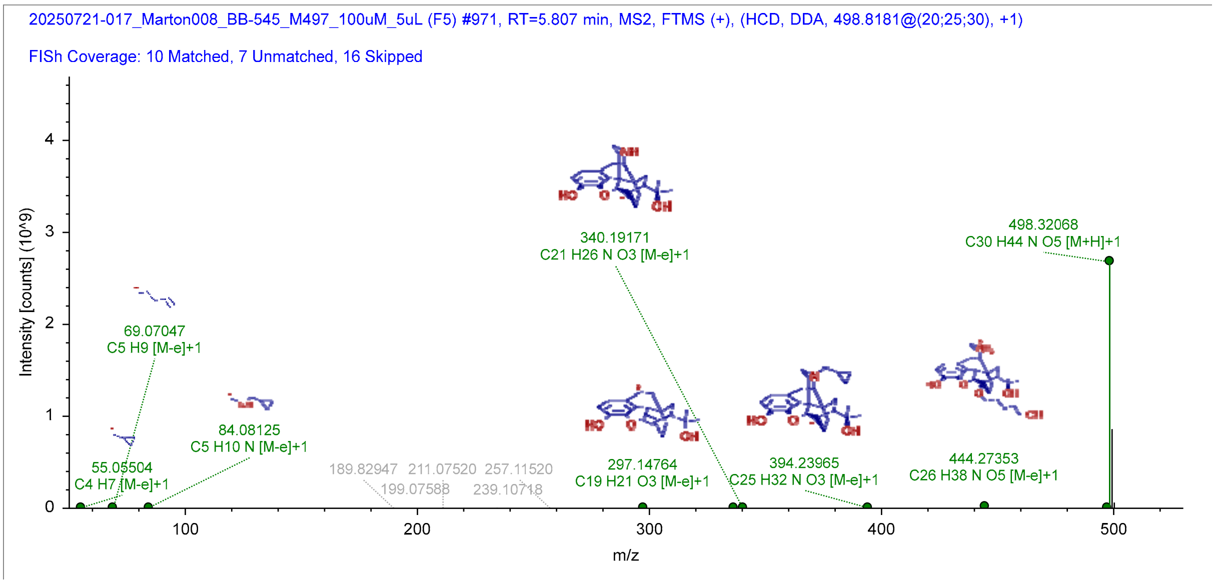

Supplement: Supplementary file 1 [file ijms-26-09427-s001.zip › ijms-3878741-supplementary.pdf]
